# IPr# – highly hindered, broadly applicable N-heterocyclic carbenes†

**DOI:** 10.1039/d1sc02619d

**Published:** 2021-07-02

**Authors:** Qun Zhao, Guangrong Meng, Guangchen Li, Carol Flach, Richard Mendelsohn, Roger Lalancette, Roman Szostak, Michal Szostak

**Affiliations:** Department of Chemistry, Rutgers University 73 Warren Street Newark NJ 07102 USA michal.szostak@rutgers.edu; Department of Chemistry, Wroclaw University F. Joliot-Curie 14 Wroclaw 50-383 Poland

## Abstract

IPr (IPr = 1,3-bis(2,6-diisopropylphenyl)imidazol-2-ylidene) represents the most important NHC (NHC = N-heterocyclic carbene) ligand throughout the field of homogeneous catalysis. Herein, we report the synthesis, catalytic activity, and full structural and electronic characterization of novel, sterically-bulky, easily-accessible NHC ligands based on the hash peralkylation concept, including IPr#, Np# and BIAN-IPr#. The new ligands have been commercialized in collaboration with Millipore Sigma: IPr#HCl, 915653; Np#HCl; 915912; BIAN-IPr#HCl, 916420, enabling broad access of the academic and industrial researchers to new ligands for reaction optimization and screening. In particular, the synthesis of IPr# hinges upon cost-effective, modular alkylation of aniline, an industrial chemical that is available in bulk. The generality of this approach in ligand design is demonstrated through facile synthesis of BIAN-IPr# and Np#, two ligands that differ in steric properties and N-wingtip arrangement. The broad activity in various cross-coupling reactions in an array of N–C, O–C, C–Cl, C–Br, C–S and C–H bond cross-couplings is demonstrated. The evaluation of steric, electron-donating and π-accepting properties as well as coordination chemistry to Au(i), Rh(i) and Pd(ii) is presented. Given the tremendous importance of NHC ligands in homogenous catalysis, we expect that this new class of NHCs will find rapid and widespread application.

## Abstract 2

We report novel, sterically-bulky, easily-accessible NHC ligands based on the hash peralkylation concept. The new ligands have been commercialized in collaboration with Millipore Sigma: IPr#HCl, 915653; Np#HCl; 915912; BIAN-IPr#HCl, 916420.

## Graphical abstract

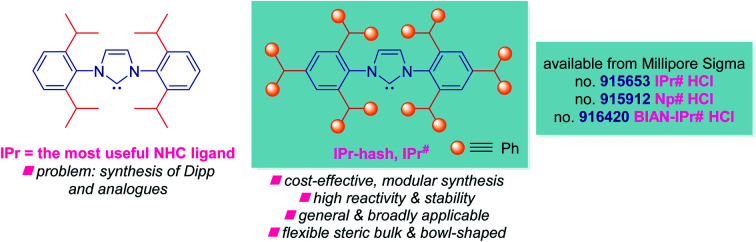

## Introduction

N-Heterocyclic carbenes (NHCs) have emerged as tremendously valuable ligands in homogeneous catalysis.^1,2^ The broad application of NHC ligands is a consequence of strong σ-donation of the carbene center^3^ and variable steric bulk of wingtip groups^4^ that are often not easily attainable using other classes of ligands, including phosphines. Furthermore, through exploiting the flexible steric bulk of the wingtips, NHC ligands allow for kinetic stabilization of metals and intermediates at unusual oxidation states,^5^ while their well-defined topology has found widespread application in fine-tuning of reactivity at the metal center.^6^

**Fig. 1:**
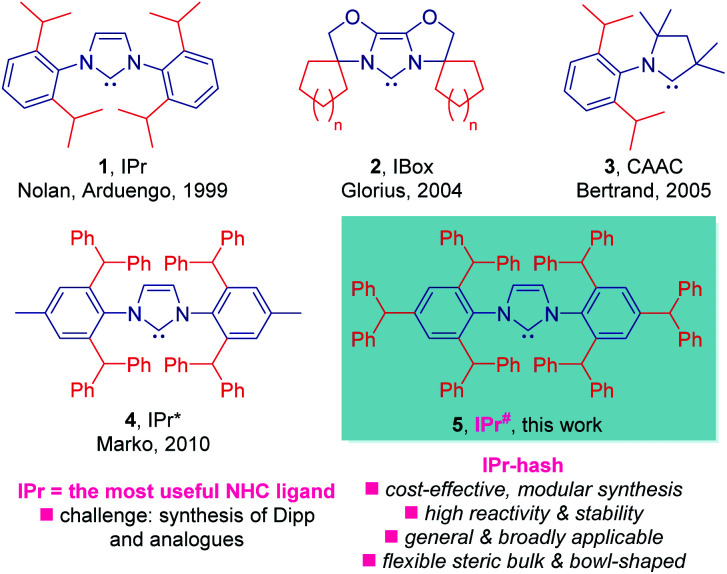
State-of-the-art of sterically-demanding N-heterocyclic carbenes in catalysis. Note that IPr-hash ligands are commercially available from Millipore Sigma (IPr#HCl, 915653; Np#HCl; 915912; BIAN-IPr#HCl, 916420).

In this context, by far the most important NHC ligand in the field of homogeneous catalysis is bulky IPr (Fig. 1, **1**) reported by Nolan and Arduengo in 1999.^1,2^ While its synthesis from Dipp (Dipp = 2,6-diisopropylaniline) is facile,^7^ the problem lies in the preparation of Dipp precursor. The preparation of Dipp is severely limited by challenges in controlling the alkylation selectivity, and the most common industrial route exploits lengthy and inflexible route through phenol alkylation.^8^

Taking inspiration from elegant studies in NHC design by Nolan,^9^ Glorius,^10^ Bertrand,^11^ Marko^12^ and others^13^ (Fig. 1), as well as following our interest in NHC catalysis^14^ and the synthesis of N-containing molecules,^15^ we sought to expand the concept of sterically-hindered, easily accessible NHC ligands. We considered that a bulky version of IPr, wherein the amine is prepared directly *via* modular alkylation of aniline, an industrial chemical that is available in bulk^16^ in a cost-effective manner should be accessible. Further, we hoped to demonstrate the generality of this approach through facile synthesis of NHC analogues with varying steric and electronic properties of the NHC topology as well as generality of the ligand design in cross-couplings by numerous bond breaking events.

## Results and discussion

**Scheme 1:**
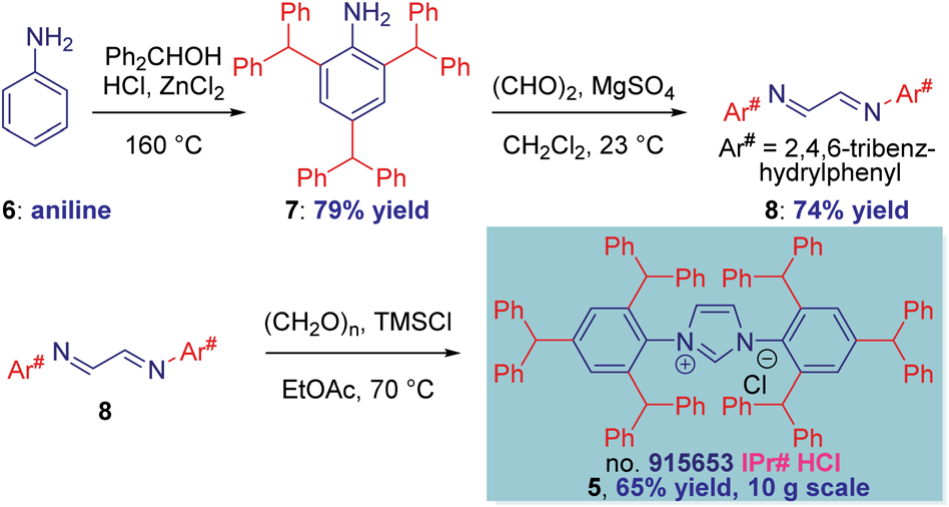
Synthesis of IPr# and the Diimine Precursors. Conditions: (a) **6** (1.0 equiv.), Ph_2_CHOH (3.5 equiv.), ZnCl_2_ (0.5 equiv.), HCl (36%, aq, 1.0 equiv.), 160 °C. (b) **7** (1.0 equiv.), (CHO)_2_ (40%, aq, 0.5 equiv.), MgSO_4_ (2.5 equiv.), 23 °C. (c) **8** (1.0 equiv.), (CH_2_O)_*n*_ (1.1 equiv.), TMSCl (1.1 equiv.), EtOH, 70 °C.

The synthesis of IPr# (Scheme 1, **5**) was selected as our starting point. It should be noted that the direct three-fold peralkylation of aniline is significantly more challenging than the alkylation of *para*-blocked toluidine as a consequence of N-/C-alkyl migration.^17^ After experimentation, we were pleased to find that 2,4,6-tribenzhydrylaniline **7** could be prepared in 79% yield (93 g, 200 mmol scale) by adding HCl (1.0 equiv.) to a solution of aniline (1.0 equiv.), diphenylmethanol (3.5 equiv.) and ZnCl_2_ (0.5 equiv.) at 160 °C.

Routinely, 70–75% yields were obtained on 10–20 mmol scale. This represents a significant improvement over the previous Friedel–Crafts method involving addition of a mixture of ZnCl_2_ and HCl at 160 °C,^12^ which led to irreproducible results. We note that alkylation of aniline at *para* position occurs last; the N-alkylation products were not observed. The synthesis of diimine **8** was smoothly effected by reacting glyoxal (1.0 equiv.) with amine **7** (2.0 equiv.) and MgSO_4_ (2.5 equiv.) (70 g, 160 mmol scale). We found that the reaction was slower than in the synthesis of IPr*,^12^ suggesting more pronounced steric character. The optimum results were obtained by monitoring the reaction progress by ^1^H NMR, and driving the reaction to completion by slightly elevating the temperature (40 °C) or adding slight excess of glyoxal, if needed. The diimine was formed as exclusively the *s*-trans isomer. The cyclization step occurred smoothly upon exposing the diimine (1.0 equiv.) and paraformaldehyde (1.1 equiv.) to TMSCl (1.1 equiv.) in EtOAc at 70 °C (25 g, 40 mmol scale).^18^ We found that this procedure allows for much milder cyclization to **5** than the HCl/ZnCl_2_ combination,^12^ which again proved problematic for large-scale runs and was riddled with retro-Friedel–Crafts products. The TMSCl procedure could be conveniently followed by color change from yellow to light grey, indicating completion of the reaction.

Crucially, the entire sequence **6** → **5** is highly practical: (1) the three steps do not require purification of the intermediates, (2) the final product is obtained after facile work-up (filtration), (3) the procedure uses industrial chemicals available in bulk, (4) the sequence is routinely performed within two days.

**Scheme 2:**
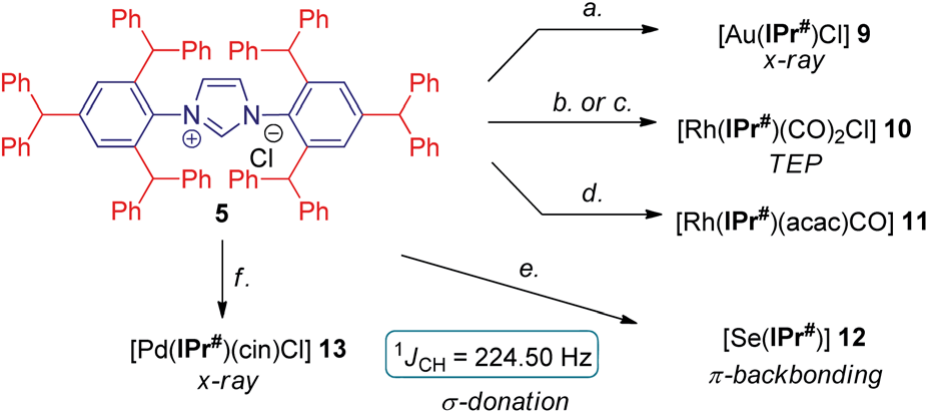
Synthesis of IPr# complexes. Conditions: (a) AuCl·Me_2_S (1.0 equiv.), K_2_CO_3_ (6.0 equiv.), acetone, 60 °C, 2 h, 90%. (b) KHMDS (1.8 equiv.), [Rh(CO)_2_Cl]_2_ (0.5 equiv.), toluene, 23 °C, 15 h, 88%. (c) [Rh(cod)Cl]_2_ (0.5 equiv.), K_2_CO_3_ (2.0 equiv.), acetone, 60 °C, 8 h, 71%, then CO, CH_2_Cl_2_, 23 °C, 15 h, 90%. (d) KO^*t*^Bu (2.0 equiv.), [Rh(acac)(CO)_2_] (1.0 equiv.), THF, 23 °C, 15 h, 93%. (e) Se (3.0 equiv.), NaHMDS (1.2 equiv.), THF, −78 to 23 °C, 15 h, 95%. (f) KO^*t*^Bu (1.1 equiv.), [Pd(cin)Cl]_2_ (0.45 equiv.), THF, 23 °C, 15 h, 89%.

**Fig. 2:**
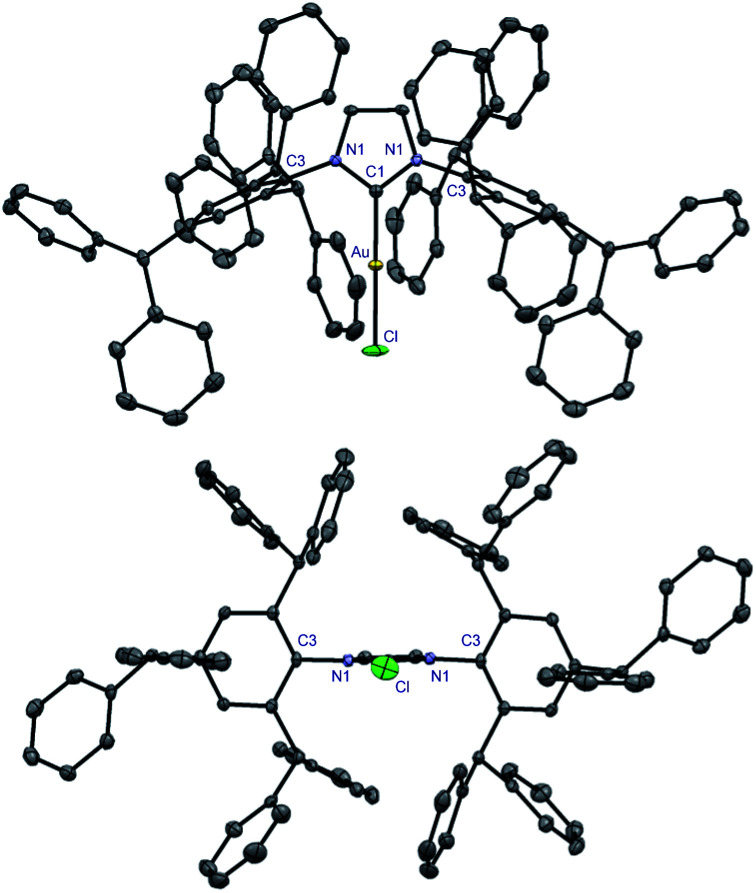
X-ray crystal structure of complex **9**. Two views: front (top); side (bottom). Hydrogen atoms have been omitted for clarity. Selected bond lengths [Å] and angles [°]: Au–C1, 1.972; Au–Cl, 2.2768; C1–N1, 1.356; C3–N1, 1.442(2); C1–Au–Cl, 180.0; N1–C1–N1, 104.9; C3–N1–C1, 122.7; N1–C1–Au, 127.5. Note the symmetry across the Cl–Au–C1 axis in **9**. CCDC 2077050.†

**Fig. 3:**
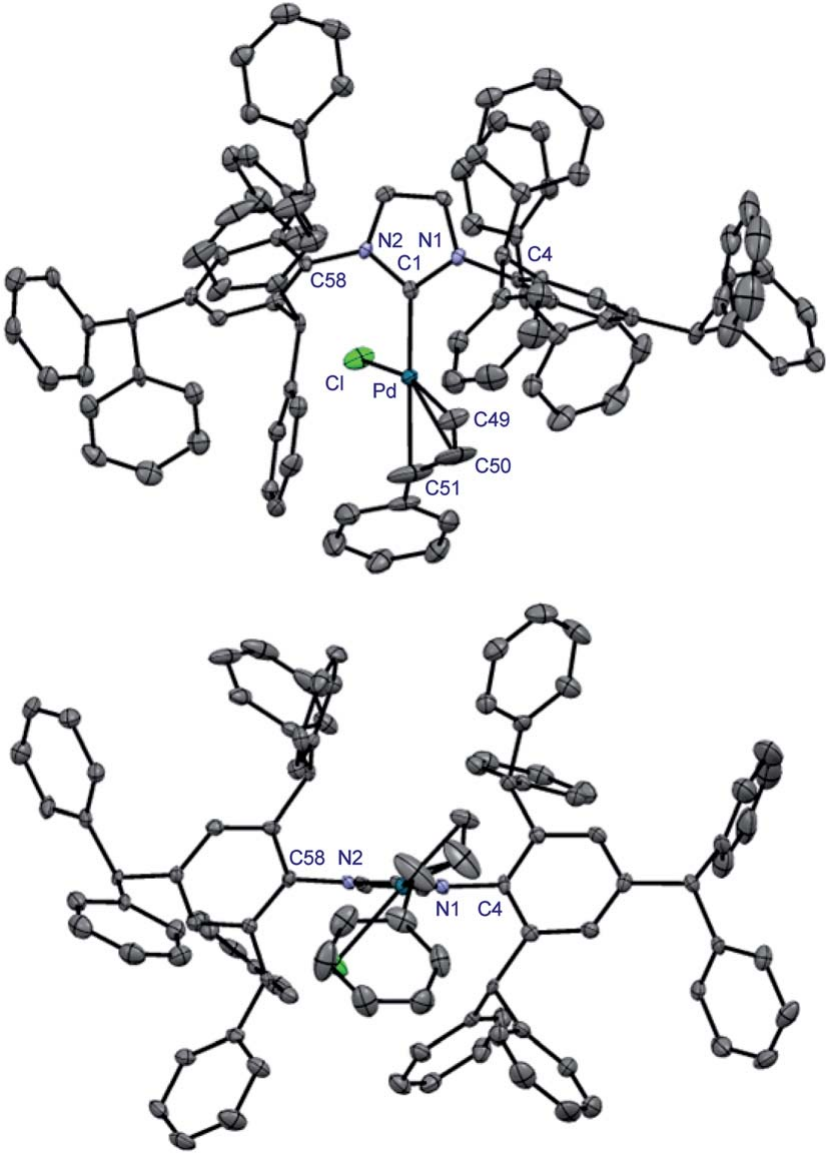
X-ray crystal structure of complex **13**. Two views: front (top); side (bottom). Hydrogen atoms have been omitted for clarity. Selected bond lengths [Å] and angles [°]: Pd–C1, 2.044(4); Pd–Cl, 2.374(1); Pd–C49, 2.121(4); Pd–C50, 2.130(6); Pd–C51, 2.210(7); C1–N1, 1.364(5); C1–N2, 1.368(4); C4–N1, 1.452(4); C58–N2, 1.440(5); C1–Pd–C49, 103.2(2); C1–Pd–C50, 138.5(2); C1–Pd–C51, 169.4(2); C49–Pd–C51, 67.0(2); C1–Pd–Cl, 93.7(1); N1–C1–N2, 103.3(3); C4–N1–C1, 124.9(3); C58–N2–C1, 124.7(3). CCDC 2077053.†

With multigram access to IPr# secured, we next comprehensively evaluated steric and electronic properties of this novel NHC ligand. As shown in Scheme 2, the gold complex [Au(IPr#)Cl] (**9**) was prepared using the general method,^19^ while Rh(i) complexes, [Rh(IPr#)(CO)_2_Cl] (**10**) and [Rh(IPr#)(acac)CO] (**11**) were prepared after generating the free carbene *in situ* by deprotonation of IPr#HCl with a slight excess of either KHMDS or KO^*t*^Bu. The Rh(i) complex (**10**) could be prepared either directly by using rhodium dicarbonyl chloro dimer [{Rh(CO)_2_(μ-Cl)}_2_] (path b) or in a mild two-step procedure *via* [Rh(IPr#)(cod)Cl]^20^ and the reaction with carbon monoxide (path c). We have also generated the selenium adduct [Se(IPr#)] (**12**) by adding the free carbene generated *in situ* to excess of selenium.^21^ The Pd(ii) complex [Pd(IPr#)(cin)Cl] (**13**) was prepared by generating the free carbene *in situ* and coordinating to the palladium cinnamyl dimer [{Pd(cin)(μ-Cl)}_2_].^22^ Importantly, all complexes were found to be stable to air and moisture. Complexes **9** and **13** were fully characterized by X-ray crystallography (Fig. 2 and 3, *vide infra*).^23^

Extensive studies by Cavallo *et al.* demonstrated the % buried volume (%*V*_bur_) and steric maps of model [Au(NHC)Cl] complexes as the best indication of quantifying the steric impact of NHC ligands.^4,24^ In our case, [Au(IPr#)Cl] is linear (C–Au–Cl, 180.0°; C–Au, 1.972 Å), making it a good model for evaluating %*V*_bur_. Thus, with the (%*V*_bur_) of 54.4%, [Au(IPr#)Cl] represents one of the bulkiest NHC ligands prepared to date (Table 1).^1,2,4^ This value compares well with the (%*V*_bur_) of 50.4% determined for [Au(IPr*)Cl] (C–Au–Cl, 178.3°; C–Au, 1.987 Å),^19^ indicating a subtle but important effect of the *para*-diphenylmethyl substitution on the steric properties of the ligand.

**Table 1:** Summary of steric and electronic parameters

NHC	%*V*_bur_ [Au]	TEP [cm^−1^]	*δ*(^77^Se) [ppm]	^1^*J*_CH_ [Hz]
IPr#	54.4	2051.8	108	224.50
IPr	45.4	2051.5	90	223.70
IPr*	50.4	2052.7	106	224.99
CAAC^Cy^	51.0^a^	2048.6	492^b^	188.53

a Menthyl instead of cyclohexyl.

b Me_2_ instead of cyclohexyl.

**Fig. 4:**
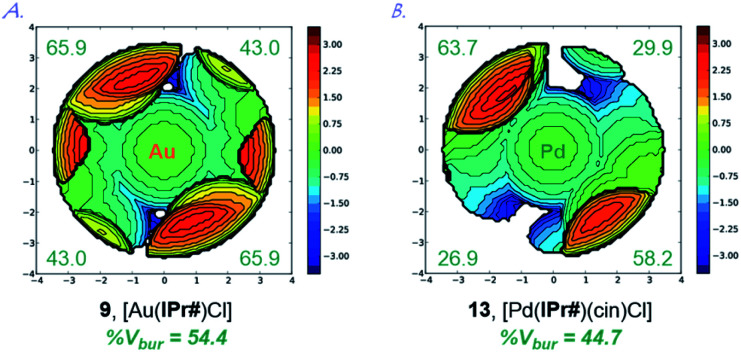
Topographical steric maps of [Au(IPr#)Cl] (**9**) and [Pd(IPr#)(cin)Cl] (**13**) showing %*V*_bur_ per quadrant.

A graphical representation of the steric mapping of the metal center in [Au(IPr#)Cl] is shown in Fig. 4A (*vide infra*).

The Tolman electronic parameter (TEP) allows to evaluate electronic properties of NHC ligands.^3^ Thus, the CO stretching frequencies of [Rh(IPr#)(CO)_2_Cl] are *ν*_sym_ = 2079.5 cm^−1^ and *ν*_asym_ = 1999.5 cm^−1^ (CH_2_Cl_2_, 0.20 M),^25^ respectively, which corresponds to a TEP of 2051.8 cm^−1^ as a combined measure of the electronic properties of the ligand. These values match well with the IPr ligand (TEP of 2051.5 cm^−1^), IPr* (TEP of 2052.7 cm^−1^) and CAAC^Cy^ (TEP of 2048.6 cm^−1^) and indicate one of the most donating 5-membered NHCs prepared to date.

Likewise, the use of selenourea adducts permits to evaluate π-backbonding from the ^77^Se NMR spectra.^21^ The *δ*_Se_ value of 108.11 ppm for [Se(IPr#)] (CDCl_3_) suggests that the expanded substitution leads to slightly higher π-accepting properties than IPr (*δ*_Se_ = 90 ppm), and IPr* (*δ*_Se_ = 106 ppm) and much lower than CAAC^Me2^ (*δ*_Se_ = 492 ppm), as expected from the C-substitution.

Furthermore, one-bond CH *J* coupling constants obtained from ^13^C satellites of the ^1^H NMR spectrum^26^ provide good indication of σ-donating properties of an NHC ligand. The value of 224.50 Hz for IPr#HCl (CDCl_3_) is consistent with this ligand being as strongly σ-donating as IPr (^1^*J*_CH_ = 223.70 Hz), but at the same time significantly more sterically-demanding and flexible. The chemical shift of the iminium proton in IPr#HCl was found at 12.6 ppm (CDCl_3_), which is significantly downfield compared with other imidazolium salts.^27^

The synthesis of [Rh(IPr#)(acac)CO] demonstrates that the extremely bulky IPr# is able to accommodate asymmetrical, κ^2^-O,O-bound ligands like acac to the metal center. Presumably, this leads to steric adjustment of the ligand topology to fit the Rh coordination plane.

This property is confirmed through the synthesis and full crystallographic characterization of [Pd(IPr#)(cin)Cl] (Fig. 3). Following the earlier reports,^9,13*a*^ Pd(ii) allyl-type complex (**13**) was selected as a model well-defined, air- and moisture-stable Pd(ii)–NHC precatalyst to evaluate the performance of IPr# in cross-coupling reactions. The X-ray crystallographic analysis revealed the (%*V*_bur_) of 44.7% with 63.7%, 29.9%, 58.2%, 26.9% for each quadrant (Fig. 4B). The values can be compared with the (%*V*_bur_) of 54.4% for the linear [Au(IPr#)Cl] with 65.9%, 43.0%, 65.9%, 43.0% for each quadrant (Fig. 4A). Thus, the IPr# ligand is capable of both (1) adjusting the steric environment, and (2) asymmetrical twisting around the metal center, which furnishes important in catalysis differentiated quadrant distribution arising from the very bulky yet flexible ligand topology. The C–Pd, Pd–Cl, and Pd–C(Ph) bond lengths of 2.044 Å, 2.374 Å, and 2.210 Å in 13 are in the range for Pd(ii)-allyl type complexes ([Pd(IPr)(cin)Cl], C–Pd, 2.027 Å; Pd–Cl, 2.357 Å; Pd–C(Ph), 2.245 Å).

It is further worth noting that diphenylmethyl substituents of the IPr# wingtips extend beyond the metal center in both **9** and **13**, which might influence both (1) substrate approach, and (2) coordination of active intermediates formed during the catalysis. We hypothesize that the addition of a *para*-substituent has steric effect due to proximity to the metal center, while two additional effects are increase of donation and inhibition of N-wingtip rotation around the N–Ar axis.

**Scheme 3:**
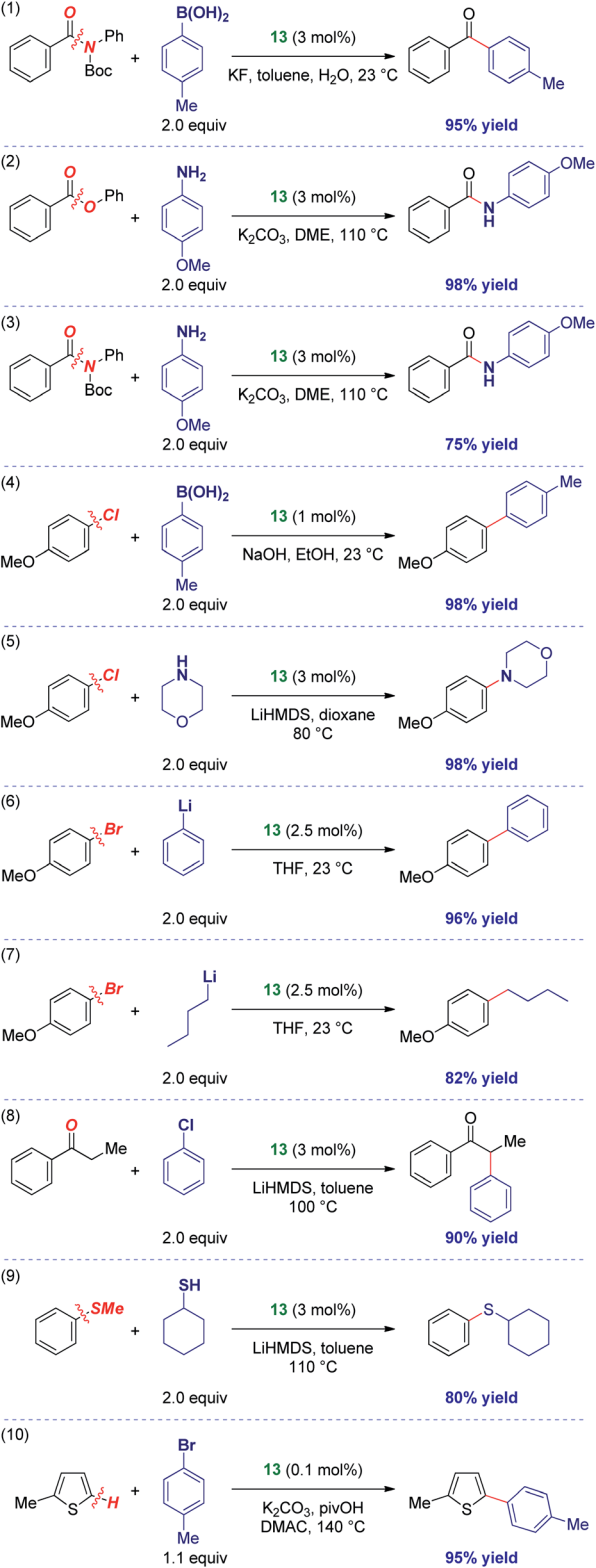
Activity of [Pd(IPr#)(cin)Cl] **13** in cross-coupling reactions. See ESI† for details.

The activity of IPr# was evaluated in various palladium-catalyzed cross-couplings (Scheme 3). As stated above, [Pd(IPr#)(cin)Cl] was selected due to the success of well-defined Pd(ii)–NHCs supported by allyl-type throw-away ligands and the potential to tune the catalyst activity by allyl modifications.^9,13*a*^ The results outlined in Scheme 3 indicate very high degree of generality of IPr#. As such, amide N–C(O) Suzuki cross-coupling (entry 1),^14^ ester C–O amidation (entry 2),^28^ amide N–C(O) transamidation (entry 3),^29^ C–Cl Suzuki cross-coupling (entry 4),^30^ C–Cl Buchwald–Hartwig amination (entry 5),^31^ C–Br Feringa coupling using both aryl- (entry 6)^32^ and challenging alkyllithium possessing β-hydrogens (entry 7),^33^ C–Cl ketone α-arylation (entry 8),^34^ C–S sulfur metathesis (entry 9)^35^ and C–H activation (entry 10)^36^ all proceeded in good to excellent yields. More importantly, these results demonstrate that the IPr# ligand is effective in an array of N–C, O–C, C–Cl, C–Br, C–S and C–H bond cross-couplings with various organometallics (B, Li, enolate, amine, sulfide) across some of the most broadly employed cross-couplings in industrial and academic settings.^1,2,6*a*–*c*^ In view of its general reactivity across numerous bonds, IPr# might offer a significant potential for catalytic reaction development.

**Scheme 4:**
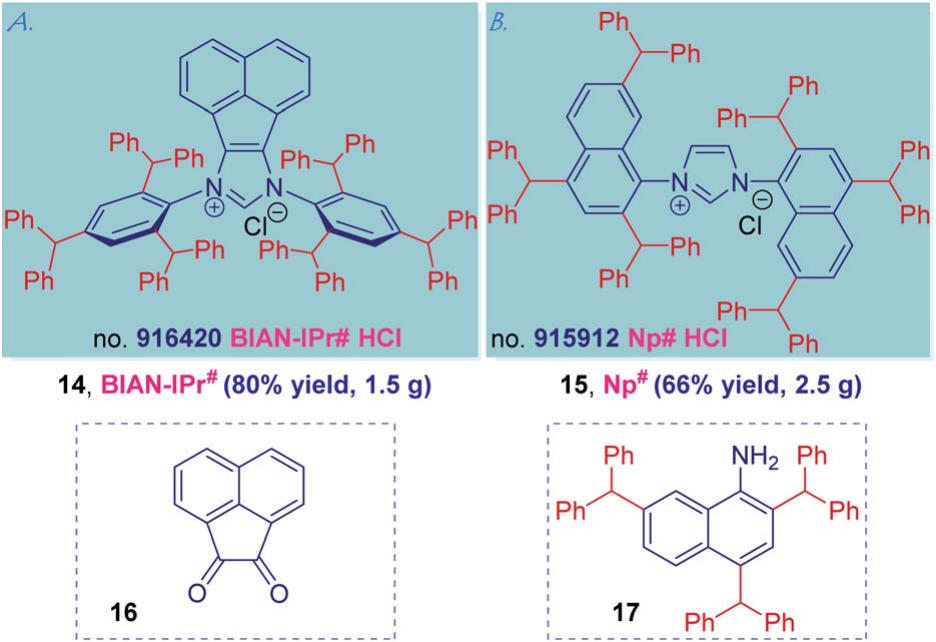
Generalization of IPr# NHCs: (a) BIAN-IPr# and (b) Np#.

Generality and two further applications of the IPr# ligand design peralkylation concept are presented in Scheme 4. We selected the synthesis of BIAN-IPr# (**14**) and Np# (**15**) as two representative examples to showcase the synthetic potential of the “hash” NHC modular framework. Thus, BIAN (BIAN = bis(imino)acenaphthalene) ligands have emerged as powerful ligands in catalysis because of the structural rigidity of C–H bonds bringing the wingtip substituents closer to the metal center as well as redox-active properties, and strong σ-donating character of the carbene center.^37^ With the access to 2,4,6-tribenzhydrylaniline **7** in hand, the synthesis of BIAN-IPr# (**14**) proceeded uneventfully readily furnishing 1.5 gram of 14 using acenaphthoquinone (**16**) as the NHC precursor (see ESI†).

In the similar vein, the *C*_2_-symmetric imidazolin-2-ylidenes bearing substituted naphthyl N-wingtips reported by Dorta have emerged as the most active NHC ancillary ligands in Pd-catalyzed cross-coupling and Ru-metathesis;^38^ however, the synthesis of 2,7-substituted naphthyl wingtips has been a major limitation. Applying the concept described herein, the synthesis of Np# (**15**) exploiting the facile synthesis of 2,4,7-tribenzhydrylnaphthalen-1-amine (**17**) by Friedel–Crafts alkylation proceeded uneventfully and furnished 2.5 g of this sterically-differentiated NHC ligand (see ESI†). Thus, the use of “hash” concept permits a modular, rapid and cost-effective ligand assembly that might be applicable to both (1) various carbene classes (*cf.* BIAN-IPr#), and (2) diverse amines (*cf.* Np#). It is further worth noting that BIAN-IPr# is the first member of the intriguing family of BIAN ligands^2*m*^ that has been commercialized (collaboration with Millipore Sigma, no. 916420, BIAN-IPr#HCl).^39,40^ Further applications of this concept are underway in our laboratory.

**Fig. 5:**
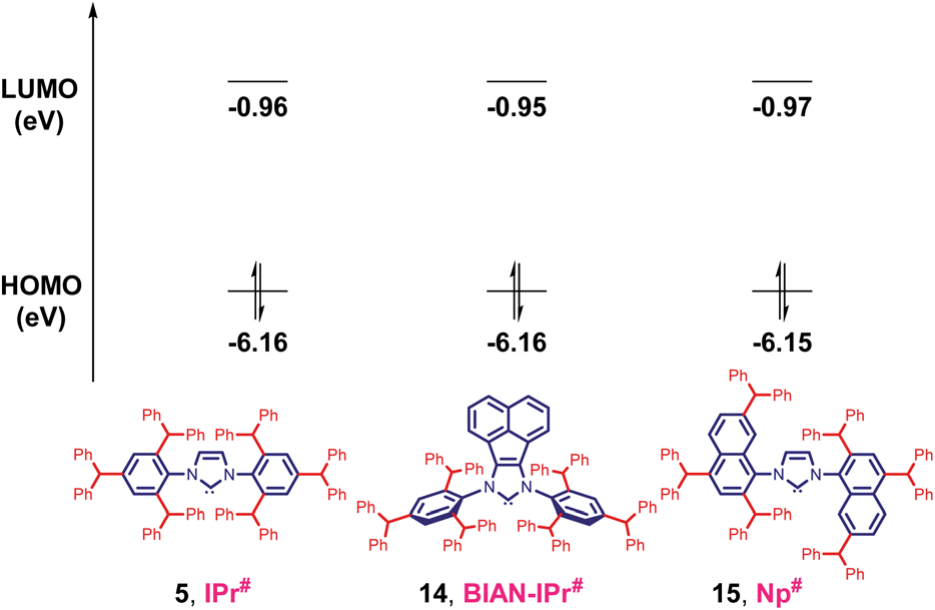
HOMO and LUMO energy levels (eV) calculated at B3LYP 6-311++g(d,p). See ESI† for details.

To further assess the effect of substitution on electronic properties of **5**, HOMO and LUMO energy levels of IPr# and other NHCs were determined at the B3LYP 6-311++g(d,p) level (Table 2, Fig. 5 and ESI†).^5*a*–*c*,11^ Computation of HOMO and LUMO provides the most accurate estimation of nucleophilicity (more σ-donating, higher HOMO) and electrophilicity (more π-accepting, lower LUMO) of NHCs, however, the values for comparison must be available at the same level of theory.

**Table 2:** HOMO and LUMO energy levels (eV) of IPr# calculated at the B3LYP 6-311++g(d,p) level^a^

NHC	HOMO [eV]	LUMO [eV]
IPr#	−6.16	−0.96
BIAN-IPr#	−6.16	−0.95
Np#	−6.15^b^	−0.97^b^
IPr*	−6.12	−0.90
IPr	−6.01	−0.48
IMes	−5.90	−0.33

a See ESI for details.

b *rac*-Np# (*C*_2_-symmetric), *meso*-Np# (*C*_S_-symmetric), −6.13 eV and −0.94 eV. *rac*-Np# is more stable than *meso*-Np# by 0.56 kcal mol^−1^ calculated at the B3LYP 6-311++g(d,p) level.

The HOMO of IPr# (−6.16 eV) is comparable with IPr (−6.01 eV), which is a routine model for σ-donating NHCs. The more hindered acenaphthene ring of BIAN-IPr# results in a similar strongly nucleophilic (σ-donating) (HOMO−1, in-plane σ-orbital, −6.16 eV), and electrophilic (π-accepting) (LUMO+1 due to required symmetry, −0.95 eV) ligand. Replacement of the N-phenyl ring with N-naphthyl results in a *C*_2_-symmetric Np# ligand similar to classic NHCs (HOMO+2, in-plane σ-orbital, −6.15 eV), with similar electrophilicity (LUMO−2 due to required symmetry, −0.97 eV). Thus, it is evident that the strong σ-donation in combination with the differentiated steric impact renders the class of IPr# ligands well-suited for homogeneous catalysis.

## Conclusions

In summary, we have developed a new class of extremely sterically-bulky, easily prepared NHC ligands by peralkylation concept. The parent ligand, IPr#, is readily accessible in three simple synthetic steps, exploiting the cost-effective, modular peralkylation of aniline, an industrial chemical that is available in bulk. Evaluation of structural and electronic properties has provided insight into the steric impact, σ-donation and π-accepting properties. The parent IPr# is one of the largest yet flexible NHC ligands developed to date, while at the same time one of the strongest σ-donors. Crucially, the potential to utilize the IPr# ligand in various Pd-catalyzed cross-coupling reactions by numerous bond breaking events has been demonstrated. The facile preparation of sterically-differentiated analogues BIAN-IPr# and Np# has been described, and highlights the capacity of this peralkylation design platform. The modularity of this approach makes is attractive for future development of NHC ligands. Given the tremendous importance of NHC ligands and the commercial availability of the developed ligands Millipore Sigma (IPr#HCl, 915653; Np#HCl; 915912; BIAN-IPr#HCl, 916420), we expect that this new class of NHCs will find rapid and widespread application.^39,40^

## Data availability

The datasets supporting this article have been uploaded as part of the supplementary material. Crystallographic data for 9 and 13 have been deposited at the CCDC under 2077050 and 2077053 and can be obtained from https://www.ccdc.cam.ac.uk/structures/.

## Author contributions

Q. Z., G. M., G. L. and M. S. conceived the work. Q. Z., G. M., G. Z. performed the synthetic and characterization experiments and analyzed the data. C. F. and R. M. contributed to the IR studies. R. L. performed crystallographic studies. R. S. performed computational studies. M. S. supervised the project and wrote the manuscript.

## Conflicts of interest

The authors declare the following competing financial interest(s): Rutgers University has filed patent(s) on ligands and precatalysts described in this manuscript (US 62/958565, Jan 8, 2020).

## Supplementary Material

SC-012-D1SC02619D-s001

SC-012-D1SC02619D-s002
