# Supplementary material for: IPr# – highly hindered, broadly applicable N-heterocyclic carbenes
Source: Chem Sci. 2021 Jul 2;12(31):10583–9. doi: 10.1039/d1sc02619d (PMC8356752; doi:10.1039/d1sc02619d)
Supplement: SC-012-D1SC02619D-s001 [file SC-012-D1SC02619D-s001.pdf]

Electronic Supplementary Material (ESI) for Chemical Science.
This journal is © The Royal Society of Chemistry 2021

Supporting Information

IPr# – Highly Hindered, Broadly Applicable N-Heterocyclic Carbenes

Qun Zhao,[†] Guangrong Meng,[†] Guangchen Li,[†] Carol Flach,[†] Richard Mendelsohn,[†] Roger Lalancette,[†] Roman Szostak,[‡] and Michal Szostak*[,†]

[†]*Department of Chemistry, Rutgers University, 73 Warren Street, Newark, NJ 07102, USA*
[‡]*Department of Chemistry, Wroclaw University, F. Joliot-Curie 14, Wroclaw 50-383, Poland*

michal.szostak@rutgers.edu

Table of Contents S1

List of Known Compounds/General Methods S2
Experimental Procedures and Characterization Data S3
Crystallographic Studies S20
Computational Methods S26
^{1}H and ^{13}C NMR Spectra S31
Cartesian Coordinates with Zero-Point Energies and Thermal Corrections S54

Corresponding Author:
Prof. Dr. M. Szostak
Department of Chemistry, Rutgers University
73 Warren Street, Newark, NJ 07102, United States
E-mail: michal.szostak@rutgers.edu

List of Known Compounds/General Methods

All starting materials reported in the manuscript have been previously described in literature and prepared by the method reported previously unless stated otherwise. All experiments were performed using standard Schlenk techniques under nitrogen or argon unless stated otherwise. All solvents were purchased at the highest commercial grade and used as received or after purification by passing through activated alumina columns or distillation from sodium/benzophenone under nitrogen. All solvents were deoxygenated prior to use. All other chemicals were purchased at the highest commercial grade and used as received. Reaction glassware was oven-dried at 140 °C for at least 24 h or flame-dried prior to use, allowed to cool under vacuum and purged with argon (three cycles). All products were identified using ^{1}H NMR analysis and comparison with authentic samples. GC and/or GC/MS analysis was used for volatile products. All yields refer to yields determined by ^{1}H NMR and/or GC or GC/MS using an internal standard (optimization) and isolated yields (preparative runs) unless stated otherwise. ^{1}H NMR and ^{13}C NMR spectra were recorded in $CDCl_3$ on Bruker spectrometers at 500 (^{1}H NMR) and 125 MHz (^{13}C NMR). All shifts are reported in parts per million (ppm) relative to residual $CHCl_3$ peak (7.26 and 77.2 ppm, ^{1}H NMR and ^{13}C NMR, respectively). All coupling constants (J) are reported in hertz (Hz). Abbreviations are: s, singlet; d, doublet; t, triplet; q, quartet; brs, broad singlet. GC-MS chromatography was performed using Agilent HP6890 GC System and Agilent 5973A inert XL EI/CI MSD using helium as the carrier gas at a flow rate of 1 mL/min and an initial oven temperature of 50 °C. The injector temperature was 250 °C. The detector temperature was 250 °C. For runs with the initial oven temperature of 50 °C, temperature was increased with a 10 °C/min ramp after 50 °C hold for 3 min to a final temperature of 220 °C, then hold at 220 °C for 15 min (splitless mode of injection, total run time of 22.0 min). High-resolution mass spectra (HRMS) were measured on a 7T Bruker Daltonics FT-MS instrument. All flash chromatography was performed using silica gel, 60 Å, 300 mesh. TLC analysis was carried out on glass plates coated with silica gel 60 F254, 0.2 mm thickness. The plates were visualized using a 254 nm UV lamp or aqueous potassium permanganate. ^{1}H NMR and ^{13}C NMR data are given for all compounds in the Supporting Experimental for characterization purposes. ^{1}H NMR, ^{13}C NMR, and HRMS data are given for all new compounds. All products have been previously reported, unless stated otherwise.

Experimental Procedures and Characterization Data

1. General Procedure for the Synthesis of IPr#HCl.

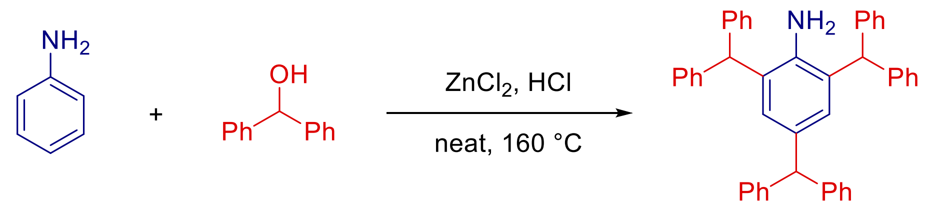

6 7

2,4,6-Tribenzhydrylaniline (7). An oven-dried 250 mL round-bottomed flask equipped with a stir bar was charged with aniline (18.6 g, 200 mmol, 1.0 equiv), diphenylmethanol (128.8 g, 700 mmol, 3.5 equiv) and anhydrous $ZnCl_2$ (13.6 g, 100 mmol, 0.5 equiv). The mixture was heated to 160 °C and melted together. Then, HCl (aq., 36%, 17 mL, 1.0 equiv) was added dropwise into the melt, and the reaction was heated at 160 °C until the mixture solidified. The reaction was cooled down to room temperature, the solid was dissolved in CH_2Cl_2 and washed sequentially with saturated NH_4Cl solution and brine. After drying over anhydrous Na_2SO_4, silica gel (50 g) was added to the solution and the mixture was filtered. Evaporation of the solvent yielded a white solid which was washed with a minimum of EtOAc to give 2,4,6-tribenzhydrylaniline as a white powder. Yield 79% (93 g) (20 mmol scale: 75%, 10 mmol scale: 72%, 5 mmol scale: 70%, 1 mmol scale: 65%). The resulting product was used directly in the next step without further purification. An analytical sample was purified for characterization purposes: ^1H NMR (500 MHz, $CDCl_3$) δ 7.26-7.05 (m, 18 H), 7.02 (d, J = 7.2 Hz, 8 H), 6.88 (d, J = 6.9 Hz, 4 H), 6.36 (s, 2 H), 5.41 (s, 2 H), 5.16 (s, 1 H), 3.36 (brs, 2 H). ^{13}C NMR (126 MHz, $CDCl_3$) δ 144.76, 142.51, 140.28, 132.40, 129.71, 129.35, 129.05, 128.87, 128.38, 127.88, 126.49, 125.66, 56.07, 52.39.

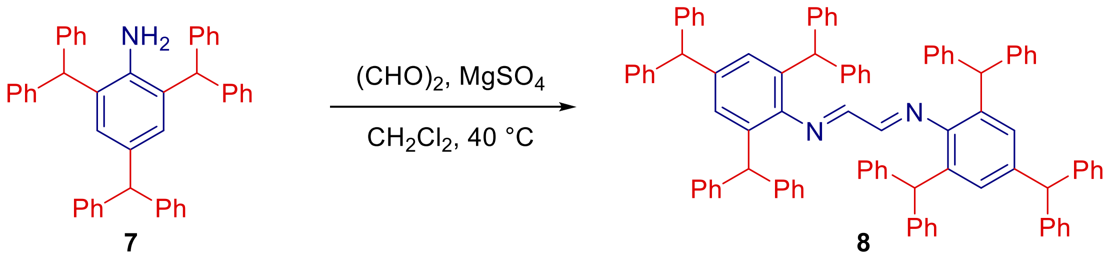

7 8

(1*E*,2*E*)-*N*[1],*N*[2]-*bis*-(2,4,6-Tribenzhydrylphenyl)ethane-1,2-diimine (8). An oven-dried 250 mL round-bottomed flask equipped with a stir bar was charged with 2,4,6-tribenzhydrylaniline (93 g,

157 mmol, 2.0 equiv), anhydrous $MgSO_4$ (47 g, 393 mmol, 5.0 equiv) and CH_2Cl_2 (200 mL). Glyoxal (aq., 40%, 8.7 ml, 0.5 equiv) and five drops of formic acid were added. The mixture was stirred at 40 °C until the reaction was judged as completed (monitored by ^{1}H NMR, typically 6-8 h). The reaction mixture was then filtered and washed with CH_2Cl_2 until the filter cake was white. The solution was collected and concentrated to give the crude solid which was further washed with a minimum of EtOAc to afford the product diimine **2** as a bright yellow powder. Yield 74% (70 g) (20 mmol scale: 80%, 10 mmol scale: 78%, 5 mmol scale: 75%, 1 mmol scale: 70%). *E*:*Z* > 95:5. The resulting product was used directly in the next step without further purification. An analytical sample was purified for characterization purposes: ^{1}H NMR (500 MHz, $CDCl_3$) δ 7.29-7.15 (m, 38 H), 6.98-6.92 (m, 24 H), 6.69 (s, 4 H), 5.32 (s, 2 H), 5.26 (s, 4 H). ^{13}C NMR (125 MHz, $CDCl_3$) δ 163.80, 147.21, 143.95, 143.62, 139.36, 131.93, 129.78, 129.32, 129.10, 128.15, 128.04, 126.20, 125.99, 56.05, 51.08.

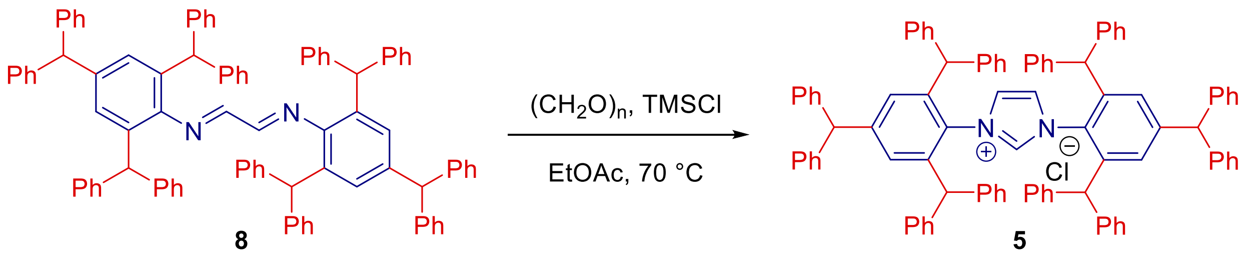

1,3-*bis*-(2,4,6-Tribenzhydrylphenyl)-1*H*-imidazol-3-ium (IPr#HCl, 5). An oven-dried 250 mL round-bottomed flask equipped with a stir bar was charged with diimine **8** (48 g, 40.0 mmol, 1.0 equiv), paraformaldehyde (1.32 g, 44.0 mmol, 1.1 equiv) and EtOAc (200 mL). The reaction mixture was stirred at 70 °C and TMSCl (4.80 g, 44.0 mmol, 1.1 equiv) was added. The resulting reaction mixture was stirred at 70 °C until the color changed from yellow to light grey (typically, 3 h). The reaction mixture was cooled down to room temperature, filtered and washed with Et_2O until the filter cake become white. The title IPr# salt was obtained after drying under vacuum as white solid in 65% yield (32.6 g, 26.0 mmol) (20 mmol scale: 66%, 10 mmol scale: 62%, 5 mmol scale: 60%, 1 mmol scale: 55%). ^{1}H NMR (500 MHz, $CDCl_3$) δ 12.84 (brs, 1H), 7.26-7.00 (m, 42 H), 6.92 (d, J = 7.8 Hz, 8 H), 6.73 (s, 4 H), 6.70 (d, J = 7.8 Hz, 8 H), 5.63 (s, 2 H), 5.35 (s, 2 H), 5.29 (s, 4 H), 2.17 (brs, 2 H). ^{13}C NMR (125 MHz, $CDCl_3$) δ 146.96, 142.53, 142.08, 141.61, 140.41, 131.39, 130.34, 129.64, 129.07, 128.96, 128.51, 128.41, 128.24, 126.83, 126.59, 126.27, 123.38, 56.04, 51.24. HRMS calcd for $C_{93}H_{73}N_2$ (M^+ – Cl) 1217.5723, found 1217.5736.

2. General Procedure for the Synthesis of [Au(IPr#)Cl].

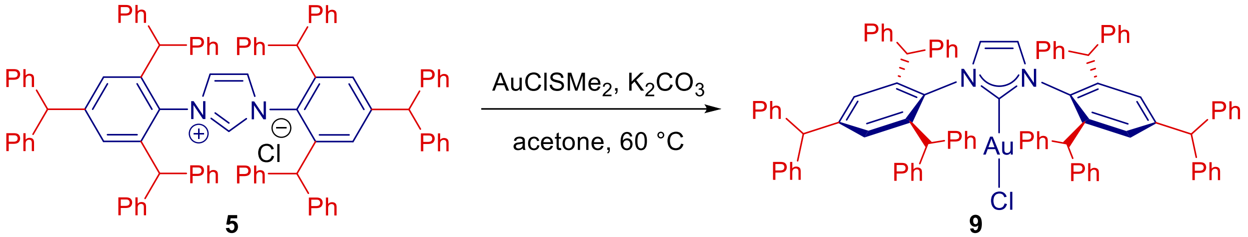

[Au(IPr#)Cl] (9). An oven-dried flask equipped with a stir bar was charged with IPr#HCl (125 mg, 0.10 mmol, 1.0 equiv), $AuClSMe_2$ (30 mg, 0.10 mmol, 1.0 equiv) and finely powdered K_2CO_3 (83 mg, 0.60 mmol, 6.0 equiv). The reaction mixture was placed under a positive pressure of argon and subjected to three evacuation/backfilling cycles under high vacuum. Acetone (2.0 ml, 0.05 M) was added and the reaction mixture was stirred at 60 °C for 16 h. After the indicated time, the reaction mixture was diluted with CH_2Cl_2 (10 mL) and filtered. The solution was collected and concentrated. The title product was obtained by trituration from hexanes as a white solid. Yield 90% (131 mg). ^1H NMR (500 MHz, $CDCl_3$) δ 7.21 (dd, J = 8.2, 6.4 Hz, 8 H), 7.18-7.12 (m, 16 H), 7.10-7.07 (m, 12 H), 6.98-6.88 (m, 16 H), 6.80 (s, 4 H), 6.80-6.77 (m, 8 H), 5.89 (s, 2 H), 5.41 (s, 2 H), 5.26 (s, 4 H). ^{13}C NMR (125 MHz, $CDCl_3$) δ 145.85, 142.96, 142.80, 142.02, 140.89, 134.16, 130.82, 129.42, 129.25, 129.22, 128.40, 128.36, 128.31, 126.59, 126.50, 126.34, 123.08, 56.16, 51.26. HRMS calcd for $C_{93}H_{72}AuClN_2K$ (M^+ + K) 1489.4740, found 1489.4760.

3. General Procedure for the Synthesis of [Rh(IPr#)(CO)2Cl]

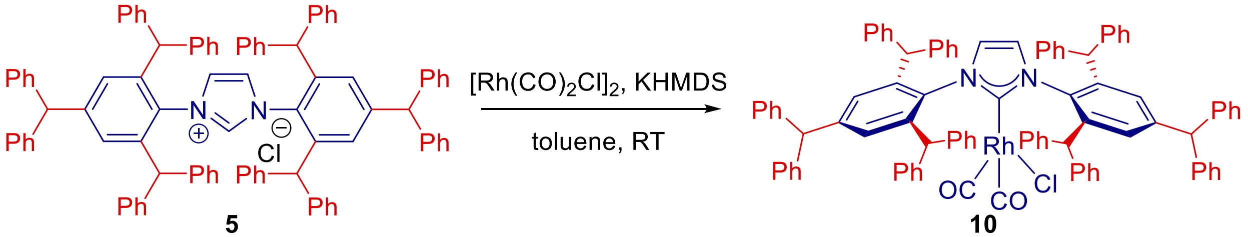

[Rh(IPr#)(CO)2Cl] (10). An oven-dried flask equipped with a stir bar was charged with IPr#HCl (125 mg, 0.10 mmol, 2.0 equiv), placed under a positive pressure of argon and subjected to three evacuation/backfilling cycles under high vacuum. Toluene (2.0 mL, 0.05 M) and KHDMS (0.50 M in toluene, 0.36 mL, 0.18 mmol, 3.6 equiv) were added and the solution was stirred at room temperature for 30 min. After the indicated time, the reaction mixture was then transferred via

cannula under argon into an oven-dried flask equipped with a stir bar containing $[Rh(CO)_2Cl]_2$ (19 mg, 0.05 mmol, 1.0 equiv) in toluene (2.0 mL). The resulting reaction mixture was was stirred at room temperature for 16 h. The reaction mixture was diluted with CH_2Cl_2 and filtered. The solution was collected and concentrated. The title product was obtained by trituration from hexanes as a white solid. Yield 88% (124 mg). ^{1}H NMR (500 MHz, $CDCl_3$) δ 7.16 (m, 32 H), 7.07 (m, 12 H), 6.95 (d, J = 7.5 Hz, 8 H), 6.78 (s, 4 H), 6.72 (d, J = 7.3 Hz, 8 H), 5.86 (s, 4 H), 5.39 (s, 2 H), 5.18 (s, 2 H). ^{13}C NMR (125 MHz, $CDCl_3$) δ 185.22, 184.79, 182.08, 181.50, 178.59, 178.22, 144.44, 144.23, 143.27, 142.77, 141.16, 135.32, 130.95, 130.21, 129.18, 129.09, 128.30, 128.18, 128.10, 127.91, 127.62, 126.29, 126.27, 126.20, 123.38, 56.17, 51.74. IR ν_{CO} (CH_2Cl_2, cm^{-1}): 2079.5 (vs), 1999.5 (vs). HRMS calcd for $C_{95}H_{72}ClN_2O_2RhK$ (M^+ + K) 1450.4002, found 1450.4003. In an alternative procedure, an oven-dried flask equipped with a stir bar was charged with IPr$^{\#}$HCl (125 mg, 0.10 mmol, 2.0 equiv), K_2CO_3 (55 mg, 0.20 mmol, 4.0 equiv), and $[Rh(COD)Cl]_2$ (25 mg, 0.05 mmol, 1.0 equiv), placed under a positive pressure of argon and subjected to three evacuation/backfilling cycles under high vacuum. Acetone (5.0 mL, 0.02 M) was added, and the resulting reaction mixture was stirred at 60 °C for 8 h. The reaction mixture was diluted with CH_2Cl_2 and filtered. The solution was collected and concentrated. The product was obtained by trituration from hexanes as a white solid. [Rh(IPr$^{\#}$)(cod)Cl]: Yield 71% (98 mg). ^{1}H NMR (500 MHz, $CDCl_3$) δ 7.40 (s, 4 H), 7.23-7.01 (m, 46 H), 6.92 (d, J = 32.4 Hz, 8 H), 6.68 (d, J = 30.4 Hz, 8 H), 5.46 (s, 2 H), 5.19 (s, 2 H), 4.99 (s, 2 H), 4.84 (s, 2 H), 3.56 (d, J = 4.9 Hz, 2 H), 2.03 (m, 2 H), 1.77-1.70 (m, 2 H), 1.66 (t, J = 7.9 Hz, 2 H), 1.51 (t, J = 6.4 Hz, 2 H). ^{13}C NMR (125 MHz, $CDCl_3$) δ 143.82, 143.51, 136.60, 130.64, 129.87, 129.66, 129.13, 128.22, 127.95, 127.58, 126.37, 126.23, 125.77, 123.58, 96.67, 68.80, 68.69, 56.36, 51.57, 50.89, 32.40, 28.49. HRMS calcd for $C_{101}H_{84}N_2Rh$ (M^+ – Cl) 1429.5796, found 1429.5767. An oven-dried flask equipped with a stir bar was charged with [Rh(IPr$^{\#}$)(cod)Cl] (69 mg, 0.05 mmol, 1.0 equiv), and CH_2Cl_2 (1.0 mL, 0.05 M) under an argon atmosphere. The solution was bubbled with CO (1 atm) and stirred at room temperature for 16 h. The resulting reaction mixture concentrated under vacuum. The title product [Rh(IPr$^{\#}$)$(CO)_2$Cl] was obtained by trituration from hexanes as a white solid. Yield 90% (63 mg). Spectroscopic properties matched those described above.

4. General Procedure for the Synthesis of [Rh(IPr[#])(acac)CO]

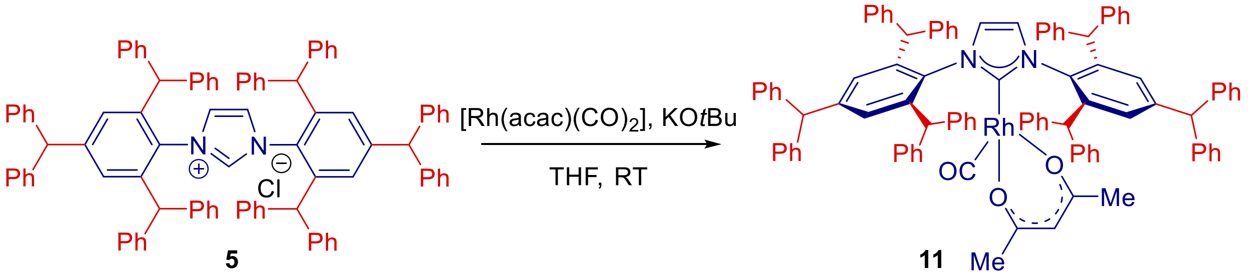

[Rh(IPr[#])(acac)CO] (11). An oven-dried flask equipped with a stir bar was charged with IPr[#]HCl (125 mg, 0.10 mmol, 1.0 equiv), KO*t*Bu (22 mg, 0.20 mmol, 2.0 equiv), and [Rh(acac)Cl_2] (26 mg, 0.10 mmol, 1.0 equiv), placed under a positive pressure of argon and subjected to three evacuation/backfilling cycles under high vacuum. THF (4.0 mL, 0.025 M) was added, and the resulting reaction mixture was stirred at room temperature for 16 h. The reaction mixture was diluted with CH_2Cl_2 and filtered. The solution was collected and concentrated. The product was obtained by trituration from hexanes as a white solid. Yield 93% (135 mg). ^{1}H NMR (500 MHz, $CDCl_3$) δ 7.20-7.14 (m, 12 H), 7.05 (d, J = 16.4 Hz, 32 H), 6.96 (d, J = 7.3 Hz, 8 H), 6.78 (s, 4 H), 6.71 (d, J = 7.5 Hz, 8 H), 6.14 (s, 4 H), 5.37 (s, 2 H), 5.31 (s, 2 H), 5.22 (s, 1 H), 2.13 (s, 3 H), 0.60 (s, 3 H). ^{13}C NMR (125 MHz, $CDCl_3$) δ 191.73, 186.26, 178.93, 143.74, 143.71, 143.43, 143.39, 141.27, 137.01, 131.09, 130.07, 129.31, 129.19, 128.14, 127.76, 126.16, 126.08, 125.93, 123.71, 100.67, 56.20, 51.36, 27.59, 25.63. HRMS calcd for $C_{99}H_{79}N_2O_3Rh$ (M^+) 1447.5174, found 1447.5169.

5. General Procedure for the Synthesis of [Se(IPr[#])]

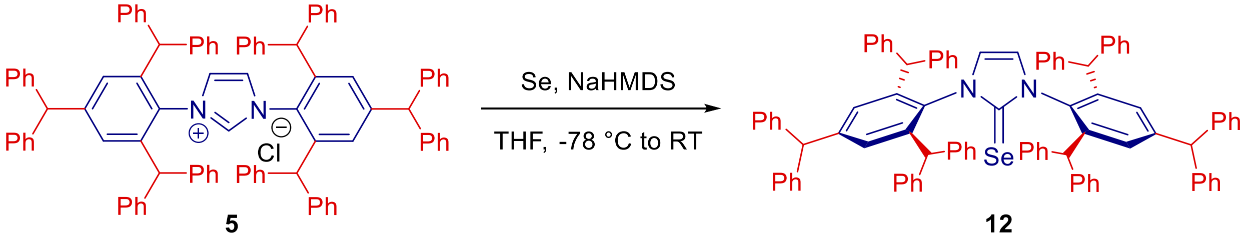

[Se(IPr[#])] (12). An oven-dried flask equipped with a stir bar was charged with IPr[#]HCl (125 mg, 0.10 mmol, 1.0 equiv) and selenium (24 mg, 0.30 mmol, 3.0 equiv), placed under a positive pressure of argon and subjected to three evacuation/backfilling cycles under high vacuum. THF (1.0 mL, 0.10 M) and NaHMDS (1.0 M in THF, 0.12 mL, 0.12 mmol, 1.2 equiv) were added at -78 °C, the resulting mixture was stirred for 30 min at -78 °C and then at room temperature for 16

h. The reaction mixture was diluted with CH_2Cl_2 and filtered. The solution was collected and concentrated. The product was obtained by trituration from diethyl ether as a white solid. Yield 95% (123 mg). ^{1}H NMR (500 MHz, $CDCl_3$) δ 7.24-7.16 (m, 22 H), 7.10 (m, 22 H), 7.00-6.97 (m, 8 H), 6.83 (s, 4 H), 6.78-6.73 (m, 8 H), 5.50 (s, 2 H), 5.44 (s, 4 H), 5.40 (s, 2 H). ^{13}C NMR (125 MHz, $CDCl_3$) δ 160.55, 144.98, 143.32, 143.26, 142.67, 141.60, 134.27, 130.86, 129.80, 129.32, 128.24, 128.18, 128.08, 126.29, 121.08, 56.25, 51.76. ^{77}Se NMR (95 MHz, $CDCl_3$) δ 108.11. HRMS calcd for $C_{93}H_{73}N_2Se$ (M^+ + H) 1297.4937, found 1297.4943.

6. General Procedure for the Synthesis of [Pd(IPr#)(cin)Cl]

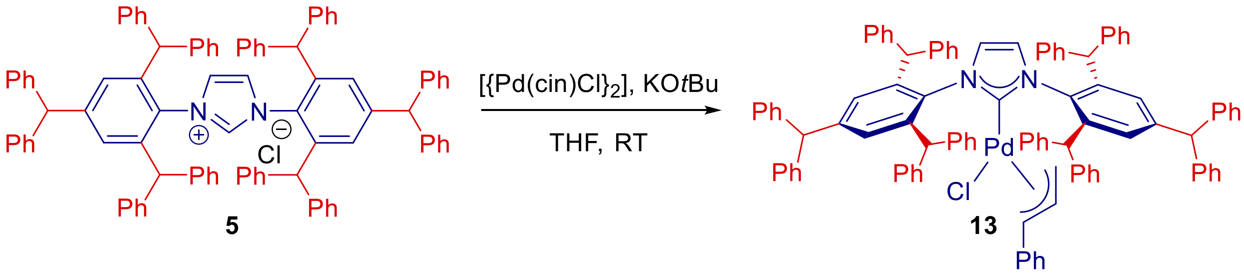

[Pd(IPr#)(cin)Cl] (13). An oven-dried flask equipped with a stir bar was charged with IPr#HCl (552 mg, 0.44 mmol, 2.2 equiv) and KO*t*Bu (56 mg, 0.48 mmol, 2.4 equiv), placed under a positive pressure of argon and subjected to three evacuation/backfilling cycles under high vacuum. THF (8.8 mL, 0.05 M) was added and the resulting reaction mixture was stirred at room temperature for 4 h. [{Pd(cin)Cl}$_2$] (103 mg, 0.20 mmol, 1.0 equiv) in THF (6.0 mL, 0.05 M) was added and the reaction mixture was stirred at room temperature for 16 h. The reaction mixture was diluted with CH_2Cl_2 and filtered. The solution was collected and concentrated. The product was obtained by trituration from diethyl ether/hexanes (1:10 v/vol) as a white solid. Yield 89% (265 mg). ^{1}H NMR (500 MHz, $CDCl_3$) δ 7.39 (m, 5 H), 7.14 (m, 44 H), 6.97 (t, J = 7.0 Hz, 8 H), 6.81 (t, J = 8.7 Hz, 8 H), 6.73 (d, J = 6.8 Hz, 4 H), 6.11 (s, 2 H), 5.64 (s, 2 H), 5.37 (d, J = 13.2 Hz, 4 H), 4.82 (m, 1 H), 4.50 (d, J = 12.9 Hz, 1 H), 2.54 (d, J = 6.8 Hz, 1 H), 1.20 (d, J = 11.6 Hz, 1 H). ^{13}C NMR (125 MHz, $CDCl_3$) δ 183.01, 144.49, 144.09, 143.35, 143.24, 143.16, 141.58, 140.51, 137.78, 136.17, 130.68, 130.57, 130.21, 129.20, 129.16, 129.12, 129.01, 128.55, 128.34, 128.30, 128.25, 128.07, 128.03, 127.58, 127.06, 126.24, 123.26, 108.88, 90.69, 56.27, 51.55, 47.41. HRMS calcd for $C_{102}H_{81}N_2Pd$ (M^+ – Cl) 1439.5434, found 1439.5406.

7. General Procedure for the Synthesis of BIAN-IPr#HCl.

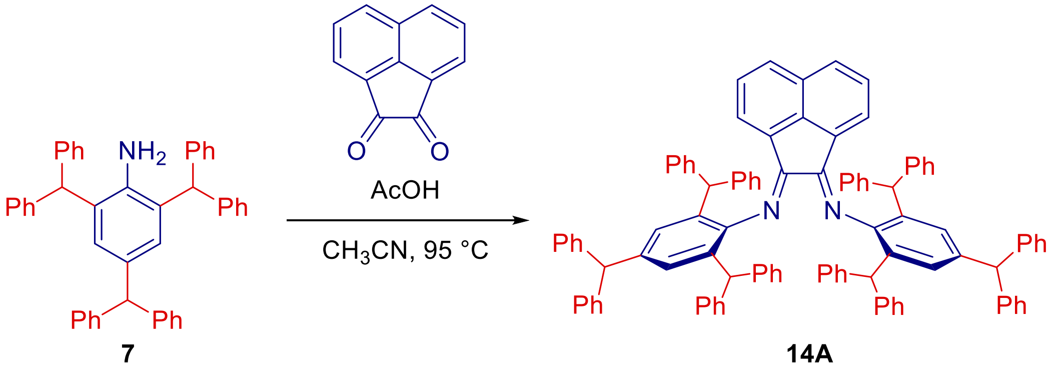

(1*E*,2*E*)-*N*[1],*N*[2]-*bis*-(2,4,6-Tribenzhydrylphenyl)acenaphthylene-1,2-diimine. An oven-dried 100 mL round-bottomed flask equipped with a stir bar was charged with acenaphthene-1,2-dione (0.546 g, 3.0 mmol) and acetonitrile (30 mL), and the resulting mixture was stirred at 95 °C for 30 min. After the indicated time, acetic acid (10 mL) and 2,4,6-tribenzhydrylaniline (3.5 g, 6.0 mmol, 2.0 equiv) were added. The reaction mixture was stirred at 95 °C (oil bath temperature) for 3 days. After the indicated time, the reaction was cooled to -20 °C, the resulting precipitate was filtered, washed with diethyl ether and dried under vacuum to afford the title product. Yield 50% (2.0 g). ^{1}H NMR (500 MHz, $CDCl_3$) δ 7.54 (d, *J* = 8.2 Hz, 2H), 7.25 (t, *J* = 7.3 Hz, 8H), 7.22-7.18 (m, 4H), 7.10-7.05 (m, 20H), 7.02-6.95 (m, 8H), 6.90-6.84 (m, 6H), 7.75-7.00 (m, 8H), 6.65-6.54 (m, 12H), 6.08 (d, *J* = 7.1 Hz, 2H), 5.67 (s, 4H), 5.47 (s, 2H). ^{13}C NMR (126 MHz, $CDCl_3$) δ 162.01, 147.14, 144.21, 143.60, 142.47, 139.70, 138.69, 131.44, 130.13, 129.61, 129.29, 129.26, 128.48, 128.13, 128.05, 127.92, 127.71, 126.50, 126.08, 125.89, 125.62, 124.12, 56.17, 51.42. HRMS calcd for $C_{102}H_{76}N_2$ (M^+ + H) 1330.6115, found 1330.6091.

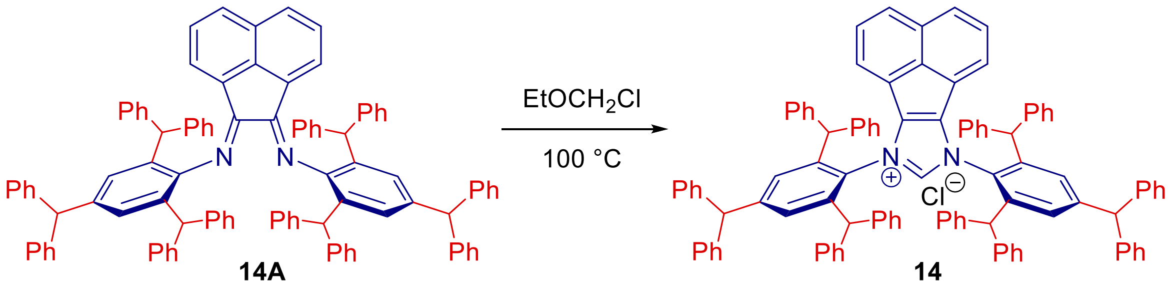

7,9-*bis*-(2,4,6-Tribenzhydrylphenyl)-7*H*-acenaphtho[1,2-*d*]imidazol-9-ium (BIAN-IPr#HCl, 14). An oven-dried round-bottomed flask equipped with a stir bar was charged with diimine **14A** (1.80 g, 1.35 mmol, 1.0 equiv) and chloromethyl ethyl ether (4.0 mL, excess, enough volume to

cover the solid) under an argon atmosphere. The resulting reaction mixture was stirred at 100 °C for 24 h. After the indicate time, the reaction was cooled down to room temperature. The reaction was diluted with diethyl ether and filtered. The product was obtained by trituration from diethyl ether as a white solid. Yield 80% (1.50 g). ^{1}H NMR (500 MHz, $CDCl_3$) δ 12.39 (s, 1H), 7.68 (d, J = 7.8 Hz, 2H), 7.2-6.94 (m, 46H), 6.78-6.62 (m, 12H), 6.54-6.45 (m, 8H), 6.22 (d, J = 6.1 Hz, 2H), 5.47 (s, 2H), 5.28 (s, 4H). ^{13}C NMR (125 MHz, $CDCl_3$) δ 147.38, 142.74, 141.46, 141.1, 140.69, 137.75, 131.43, 129.81, 129.81, 129.43, 129.31, 129.27, 128.91, 128.5, 128.47, 128.09, 126.84, 126.74, 126.60, 126.49, 122.98, 122.08, 56.31, 51.72. HRMS calcd for $C_{103}H_{77}N_2$ (M^+ – Cl) 1342.6115, found 1342.6118.

8. General Procedure for the Synthesis of Np#HCl.

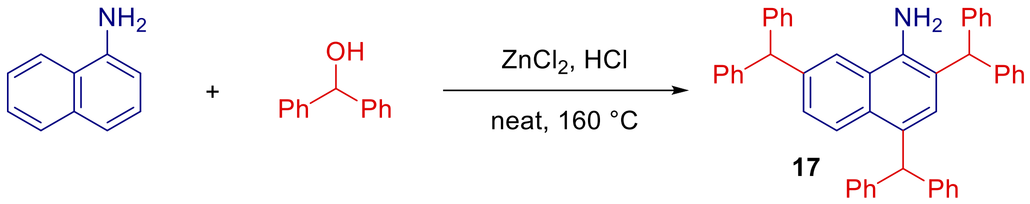

2,4,7-Tribenzhydrylnaphthalen-1-amine (17). An oven-dried 50 mL round-bottomed flask equipped with a stir bar was charged with naphthalen-1-amine (1.62 g, 11.3 mmol, 1.0 equiv), diphenylmethanol (7.3 g, 39.6 mmol, 3.5 equiv) anhydrous $ZnCl_2$ (775 mg, 5.7 mmol, 0.5 equiv). The mixture was heated to 160 °C and melted together. Then, HCl (aq., 36%, 0.96 mL, 1.0 equiv) was added dropwise into the melt, and the reaction was heated at 160 °C until the mixture solidified. The reaction was cooled down to room temperature, the solid was dissolved in CH_2Cl_2 and washed sequentially with saturated NH_4Cl solution and brine. After drying over anhydrous Na_2SO_4, silica gel (5 g) was added to the solution and the mixture was filtered. Evaporation of the solvent yielded a white solid which was washed with a minimum of EtOAc to give 2,4,7-tribenzhydrylnaphthalen-1-amine as a white powder. Yield 85% (6.15 g). ^{1}H NMR (500 MHz, $CDCl_3$) δ 7.90 (d, J = 8.6 Hz, 2 H), 7.51 (s, 1 H), 7.36-7.30 (m, 4 H), 7.29-7.13 (m, 19 H), 7.03 (d, J = 7.0 Hz, 4 H), 7.00 (d, J = 7.0 Hz, 4 H), 6.39 (s, 1 H), 6.10 (s, 1 H), 5.72 (s, 1 H), 5.61 (s, 1 H), 3.93 (brs, 2 H). ^{13}C NMR (126 MHz, $CDCl_3$) δ 143.99, 143.62, 142.38, 140.10, 137.74, 130.22, 130.00, 129.51, 129.37, 129.30, 129.23, 128.41, 128.30, 128.09, 127.66, 126.44, 126.36,

125.92, 124.92, 123.91, 122.29, 121.06, 57.05, 52.77, 52.42. HRMS calcd for $C_{49}H_{39}NK$ (M^+ + K) 680.2714, found 680.2741.

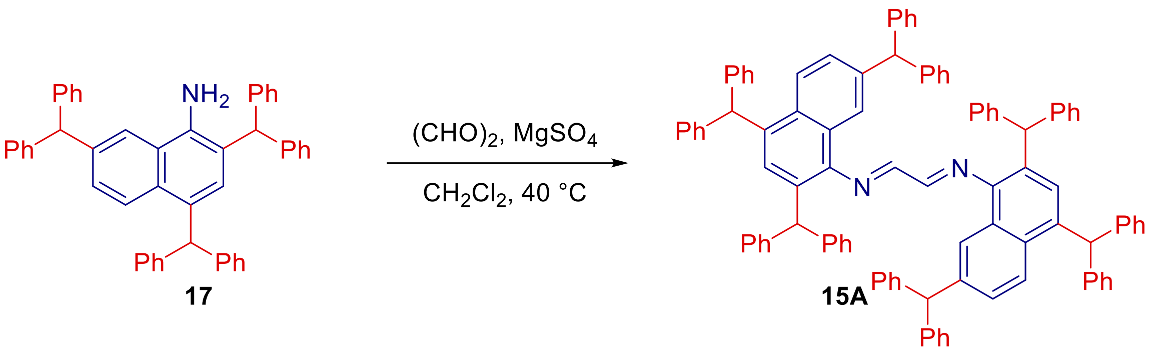

(1*E*,2*E*)-*N*[1],*N*[2]-*bis*-(2,4,7-Tribenzhydrylnaphthalen-1-yl)ethane-1,2-diimine (15A). An oven-dried 100 mL round-bottomed flask equipped with a stir bar was charged with 2,4,7-tribenzhydrylnaphthalen-1-amine (5.51 g, 8.6 mmol, 2.0 equiv), anhydrous $MgSO_4$ (2.58 g, 21.5 mmol, 5.0 equiv) and CH_2Cl_2 (50 mL). Glyoxal (aq., 40%, 0.48 mL, 0.5 equiv) and five drops of formic acid were added. The mixture was stirred at 40 °C until the reaction was judged as completed (monitored by ^1H NMR, typically 6-8 h). The reaction mixture was then filtered and washed with CH_2Cl_2 until the filter cake was white. The solution was collected and concentrated to give the crude solid which was further washed with a minimum of EtOAc to afford the product diimine **15A** as a bright yellow powder. *E*:*Z* > 95:5. Yield 80% (4.50 g, 3.45 mmol). ^1H NMR (500 MHz, $CDCl_3$) δ 8.04 (d, *J* = 8.6 Hz, 2 H), 7.96 (s, 2 H), 7.72 (s, 2 H), 7.36-7.15 (m, 46 H), 7.13-7.07 (m, 8 H), 7.03-6.93 (m, 8 H), 6.79 (s, 2 H), 6.25 (s, 2 H), 5.74 (s, 2 H), 5.64 (s, 2 H). ^{13}C NMR (126 MHz, $CDCl_3$) δ 164.57, 144.69, 143.66, 143.55, 143.37, 141.09, 136.51, 130.36, 129.79, 129.30, 129.23, 128.29, 128.25, 128.19, 127.97, 127.29, 126.36, 126.23, 126.16, 126.04, 124.36, 124.04, 57.09, 52.85, 51.36. HRMS calcd for $C_{100}H_{77}N_2$ (M^+ + H) 1306.6115, found 1306.6148.

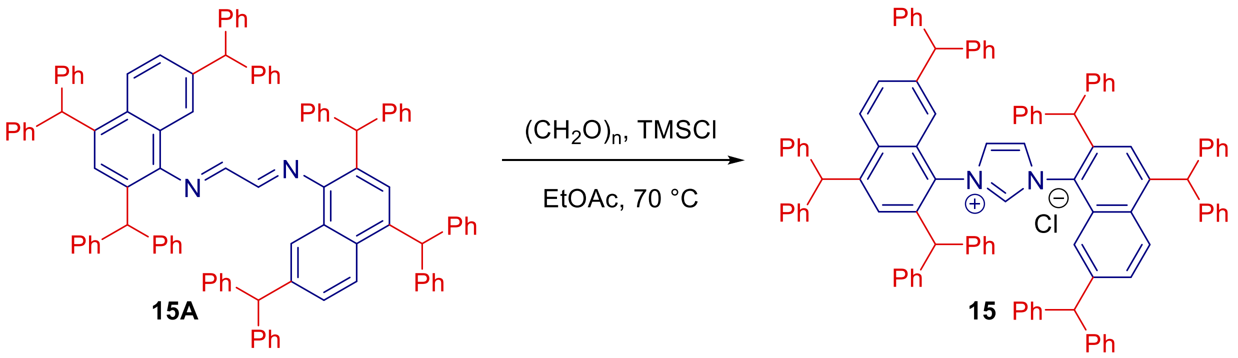

15A 15

1,3-*bis*-(2,4,7-Tribenzhydrylnaphthalen-1-yl)-1*H*-imidazol-3-ium (Np#HCl, 15). An oven-dried 100 mL round-bottomed flask equipped with a stir bar was charged with diimine **15A** (3.65 g, 2.8 mmol, 1.0 equiv), paraformaldehyde (92 g, 3.08 mmol, 1.1 equiv) and EtOAc (50 mL). The reaction mixture was stirred 70 °C and TMSCl (336 mg, 3.08 mmol, 1.1 equiv) was added. The resulting reaction was stirred at 70 °C until the color changed from yellow to light grey (typically, 3 hours). The reaction mixture was cooled down to room temperature, filtered and washed with Et_2O until the filter cake become white. The title Np-IPr# was obtained after drying under vacuum as white solid in 66% yield (2.5 g, 1.85 mmol). Mixture of rotamers, 7:3. ^{1}H NMR (500 MHz, DMSO-*d*^{6}) δ 10.67 (s, 0.4 H), 9.35 (s, 0.6 H), 8.56 (s, 1.4 H), 8.31 (d, *J* = 9.0 Hz, 2 H), 8.00 (s, 0.6 H), 7.55-7.46 (m, 2 H), 7.35-6.86 (m, 54 H), 6.83-6.71 (m, 6 H), 6.68-6.60 (m, 4 H), 6.48 (s, 2 H), 5.77 (s, 0.6 H), 5.62 (s, 1.4 H), 5.27 (s, 1.4 H), 5.19 (s, 0.6 H). ^{13}C NMR (126 MHz, DMSO-*d*^{6}) δ 172.44, 170.81, 144.28, 144.01, 143.85, 143.30, 143.07, 142.98, 142.91, 142.71, 142.63, 142.36, 141.88, 141.60, 141.36, 140.93, 140.64, 138.57, 137.54, 129.79, 129.58, 129.49, 129.42, 129.35, 129.23, 129.19, 129.16, 129.11, 129.09, 129.00, 128.94, 128.84, 128.81, 128.77, 128.67, 128.02, 127.75, 127.61, 127.51, 127.34, 127.27, 127.16, 127.14, 127.09, 127.00, 126.92, 126.86, 126.81, 125.94, 125.59, 122.69, 121.76, 60.23, 56.57, 56.39, 52.09, 52.03, 51.17, 51.10. HRMS calcd for $C_{101}H_{77}N_2$ (M^+ – Cl) 1318.6115, found 1318.6105.

9. Activity of [Pd(IPr#)(cin)Cl] in Cross-Coupling Reactions

9.1. N–C(O) Cleavage: Suzuki–Miyaura Amide Cross-Coupling

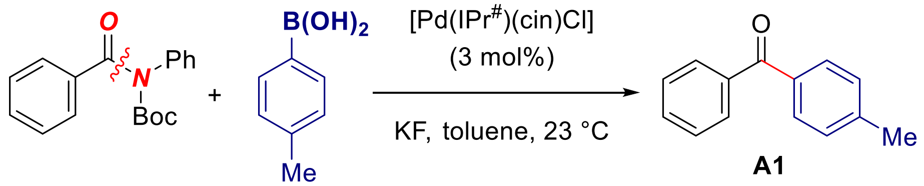

Phenyl(*p*-tolyl)methanone (A1). An oven dried vial equipped with a stir bar was charged with an amide substrate (29.7 mg, 0.10 mmol, 1.0 equiv), boronic acid (27.2 mg, 0.20 mmol, 2.0 equiv), potassium fluoride (17.4 mg, 0.30 mmol, 3.0 equiv), [(IPr#)Pd(cin)Cl] (4.4 mg, 0.003 mmol, 3 mol%), placed under a positive pressure of argon, and subjected to three evacuation/backfilling cycles under high vacuum. Toluene (0.40 mL, 0.25 M) and water (0.50 mmol, 5.0 equiv) were added with vigorous stirring at room temperature, and the reaction mixture was stirred at room temperature for 16 h. After the indicated time, the reaction mixture was diluted with CH_2Cl_2 (10 mL), filtered, and concentrated. The sample was analyzed by ^{1}HNMR ($CDCl_3$, 500 MHz) and GC-MS to obtain conversion, selectivity and yield using internal standard and comparison with authentic samples. Purification by chromatography on silica gel (EtOAc/hexanes) afforded the title product. Yield 95% (18.6 mg). ^{1}H NMR (500 MHz, $CDCl_3$) δ 7.78 (d, J = 7.8 Hz, 2H), 7.72 (d, J = 7.8 Hz, 2H), 7.60-7.55 (m, 1 H), 7.50-7.44 (m, 2 H), 7.30-7.26 (m, 2 H), 2.44 (s, 3 H). ^{13}C NMR (126 MHz, $CDCl_3$) δ 196.47, 143.21, 137.96, 134.88, 132.12, 130.29, 129.91, 128.95, 128.18, 21.65.

9.2. O–C(O) Cleavage: Buchwald-Hartwig Ester Cross-Coupling

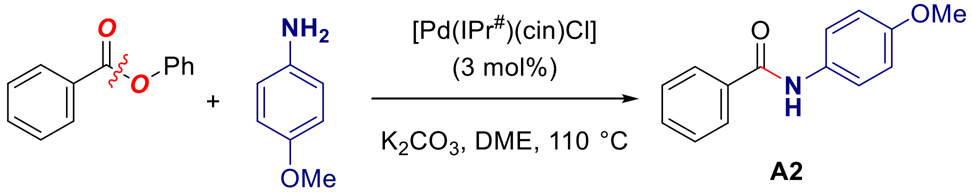

***N*-(4-Methoxyphenyl)benzamide (A2).** An oven dried vial equipped with a stir bar was charged with an ester substrate (19.8 mg, 0.10 mmol, 1.0 equiv), amine (24.6 mg, 0.20 mmol, 2.0 equiv), potassium carbonate (41.4 mg, 0.30 mmol, 3.0 equiv), [(IPr#)Pd(cin)Cl] (4.4 mg, 0.003 mmol, 3 mol%), placed under a positive pressure of argon, and subjected to three evacuation/backfilling cycles under high vacuum. DME (0.40 mL, 0.25 M) was added with vigorous stirring and the

reaction mixture was stirred at 110 °C for 16 h. After the indicated time, the reaction mixture was diluted with CH_2Cl_2 (10 mL), filtered, and concentrated. The sample was analyzed by ^{1}HNMR ($CDCl_3$, 500 MHz) and GC-MS to obtain conversion, selectivity and yield using internal standard and comparison with authentic samples. Purification by chromatography on silica gel (EtOAc/hexanes) afforded the title product. Yield 98% (22.2 mg). ^{1}H NMR (500 MHz, $CDCl_3$) δ 7.85 (d, J = 8.2 Hz, 3H), 7.57-7.50 (m, 2 H), 7.49-7.44 (m, 2 H), 6.89 (d, J = 8.2 Hz, 2H), 3.81 (s, 3 H). ^{13}C NMR (126 MHz, $CDCl_3$) δ 165.63, 156.59, 135.00, 131.65, 130.98, 128.70, 126.96, 122.11, 114.20, 55.48.

9.3. N–C(O) Cleavage: Buchwald-Hartwig Amide Cross-Coupling (Transamidation)

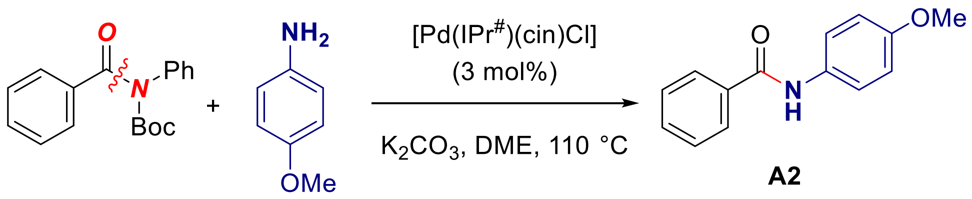

***N*-(4-Methoxyphenyl)benzamide (A2).** An oven dried vial equipped with a stir bar was charged with an amide substrate (29.7 mg, 0.10 mmol, 1.0 equiv), amine (24.6 mg, 0.20 mmol, 2.0 equiv), potassium carbonate (41.4 mg, 0.30 mmol, 3.0 equiv), [(IPr#)Pd(cin)Cl] (4.4 mg, 0.003 mmol, 3 mol%), placed under a positive pressure of argon, and subjected to three evacuation/backfilling cycles under high vacuum. DME (0.40 mL, 0.25 M) was added with vigorous stirring and the reaction mixture was stirred at 110 °C for 16 h. After the indicated time, the reaction mixture was diluted with CH_2Cl_2 (10 mL), filtered, and concentrated. The sample was analyzed by ^{1}HNMR ($CDCl_3$, 500 MHz) and GC-MS to obtain conversion, selectivity and yield using internal standard and comparison with authentic samples. Purification by chromatography on silica gel (EtOAc/hexanes) afforded the title product. Yield 75% (17.1 mg). ^{1}H NMR (500 MHz, $CDCl_3$) δ 7.85 (d, J = 8.2 Hz, 3H), 7.57-7.50 (m, 2 H), 7.49-7.44 (m, 2 H), 6.89 (d, J = 8.2 Hz, 2H), 3.81 (s, 3 H). ^{13}C NMR (126 MHz, $CDCl_3$) δ 165.63, 156.59, 135.00, 131.65, 130.98, 128.70, 126.96, 122.11, 114.20, 55.48.

9.4. C–Cl Cleavage: Suzuki–Miyaura Cross-Coupling

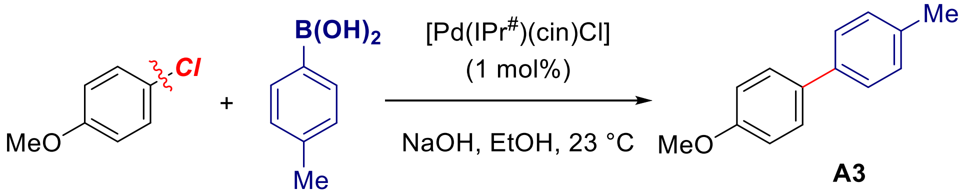

4-Methoxy-4'-methyl-1,1'-biphenyl (A3). An oven dried vial equipped with a stir bar was charged with an aryl chloride substrate (14.2 mg, 0.10 mmol, 1.0 equiv), boronic acid (27.2 mg, 0.20 mmol, 2.0 equiv), NaOH (12 mg, 0.30 mmol, 3.0 equiv), [(IPr#)Pd(cin)Cl] (1.5 mg, 0.001 mmol, 1.0 mol%), placed under a positive pressure of argon, and subjected to three evacuation/backfilling cycles under high vacuum. EtOH (0.40 mL, 0.25 M) was added with vigorous stirring at room temperature and the reaction mixture was stirred at room temperature for 16 h. After the indicated time, the reaction mixture was diluted with CH_2Cl_2 (10 mL), filtered, and concentrated. The sample was analyzed by ^{1}HNMR ($CDCl_3$, 500 MHz) and GC-MS to obtain conversion, selectivity and yield using internal standard and comparison with authentic samples. Purification by chromatography on silica gel (EtOAc/hexanes) afforded the title product. Yield 98% (19.4 mg). ^{1}H NMR (500 MHz, $CDCl_3$) δ 7.54 (d, J = 8.7 Hz, 2H), 7.48 (d, J = 7.4 Hz, 2H), 7.26 (d, J = 7.4 Hz, 2H), 7.48 (d, J = 8.7 Hz, 2H), 7.60-7.55 (m, 1 H), 7.50-7.44 (m, 2 H), 3.88 (s, 3 H), 2.41 (s, 3 H). ^{13}C NMR (126 MHz, $CDCl_3$) δ 158.91, 137.95, 136.33, 133.73, 129.41, 127.93, 126.57, 114.13, 77.25, 21.03.

9.5. C–Cl Cleavage: Buchwald-Hartwig Cross-Coupling

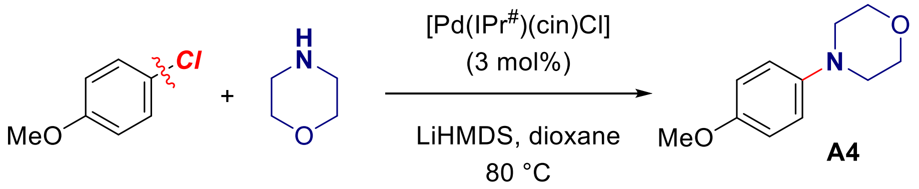

4-(4-Methoxyphenyl)morpholine (A4). An oven dried vial equipped with a stir bar was charged with an aryl chloride substrate (14.2 mg, 0.10 mmol, 1.0 equiv), morpholine (17.4 mg, 0.20 mmol, 2.0 equiv), [(IPr#)Pd(cin)Cl] (4.4 mg, 0.003 mmol, 3 mol%), placed under a positive pressure of argon, and subjected to three evacuation/backfilling cycles under high vacuum. Dioxane (0.40 mL, 0.25 M) was added and LiHMDS (1.0 M in THF, 0.30 mmol, 3.0 equiv) were added with vigorous stirring at room temperature and the reaction was stirred at 80 °C for 16 h.

After the indicated time, the reaction mixture was diluted with CH_2Cl_2 (10 mL), washed with water (1 x 10 mL), extracted with CH_2Cl_2 (2 x 10 mL), dried over $MgSO_4$, filtered and concentrated. The sample was analyzed by ^{1}HNMR ($CDCl_3$, 500 MHz) and GC-MS to obtain conversion, selectivity and yield using internal standard and comparison with authentic samples. Purification by chromatography on silica gel (EtOAc/hexanes) afforded the title product. Yield 98% (18.9 mg). ^{1}H NMR (500 MHz, $CDCl_3$) δ 6.90-6.85 (m, 4 H), 3.86 (brs, 4 H), 3.77 (brs, 3 H), 3.05 (brs, 4 H). ^{13}C NMR (126 MHz, $CDCl_3$) δ 153.96, 145.62, 117.81, 114.49, 67.02, 55.56, 50.81.

9.6. C–Br Cleavage: Feringa Cross-Coupling with Aryllithium

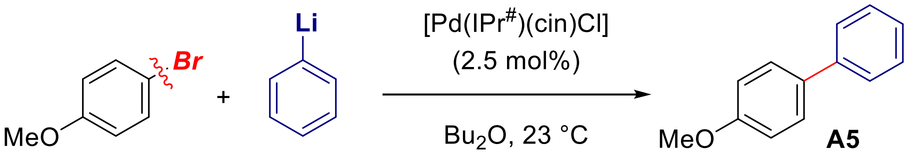

A5

4-Methoxy-1,1'-biphenyl (A5). An oven dried vial equipped with a stir bar was charged with an aryl bromide substrate (187 mg, 1.0 mmol, 1.0 equiv), PhLi (1.9 M in Bu_2O, 2.0 mmol, 2.0 equiv) and [(IPr#)Pd(cin)Cl] (36.9 mg, 0.025 mmol, 2.5 mol%) at room temperature under argon and stirred for 10 min. After the indicated time, the reaction mixture was diluted with CH_2Cl_2 (10 mL), washed with water (1 x 10 mL), extracted with CH_2Cl_2 (2 x 10 mL), dried over $MgSO_4$, filtered and concentrated. The sample was analyzed by ^{1}HNMR ($CDCl_3$, 500 MHz) and GC-MS to obtain conversion, selectivity and yield using internal standard and comparison with authentic samples. Purification by chromatography on silica gel (EtOAc/hexanes) afforded the title product. Yield 96% (177 mg). ^{1}H NMR (500 MHz, $CDCl_3$) δ 7.61-7.52 (m, 4 H), 7.58-7.40 (m, 2 H), 7.36-7.30 (m, 1 H), 7.03-6.96 (m, 2 H), 3.88 (s, 3 H). ^{13}C NMR (126 MHz, $CDCl_3$) δ 159.15, 140.84, 133.80, 128.73, 128.17, 126.75, 126.67, 114.21, 55.37.

9.7. C–Br Cleavage: Feringa Cross-Coupling with Alkyllithium

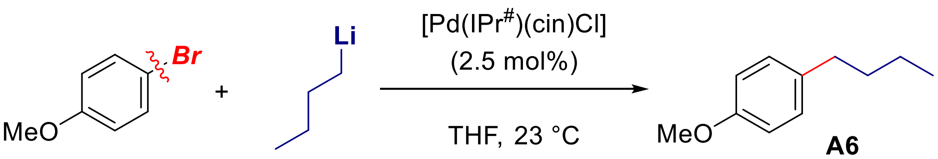

A6

1-Butyl-4-methoxybenzene (A6). An oven dried vial equipped with a stir bar was charged with an aryl bromide substrate (187 mg, 1.0 mmol, 1.0 equiv), *n*BuLi (2.5 M in hexanes, 2.0 mmol, 2.0 equiv) and [(IPr$^{\#}$)Pd(cin)Cl] (36.9 mg, 0.025 mmol, 2.5 mol%) at room temperature under argon and stirred for 10 min. After the indicated time, the reaction mixture was diluted with CH_2Cl_2 (10 mL), washed with water (1 x 10 mL), extracted with CH_2Cl_2 (2 x 10 mL), dried over $MgSO_4$, filtered and concentrated. The sample was analyzed by ^{1}HNMR ($CDCl_3$, 500 MHz) and GC-MS to obtain conversion, selectivity and yield using internal standard and comparison with authentic samples. Purification by chromatography on silica gel (EtOAc/hexanes) afforded the title product. Yield 82% (134 mg). ^{1}H NMR (500 MHz, $CDCl_3$) δ 7.12 (d, J = 7.8 Hz, 2H), 6.85 (d, J = 7.8 Hz, 2H), 3.81 (s, 3 H), 2.59 (t, J = 7.6 Hz, 2H), 1.62-1.56 (m, 2 H), 1.40-1.34 (m, 2 H), 0.95 (t, J = 7.4 Hz, 3H). ^{13}C NMR (126 MHz, $CDCl_3$) δ 157.60, 135.04, 129.26, 113.65, 55.26, 34.74, 33.93, 22.32, 13.98.

9.8. C–Cl Cleavage: α-Ketone Arylation

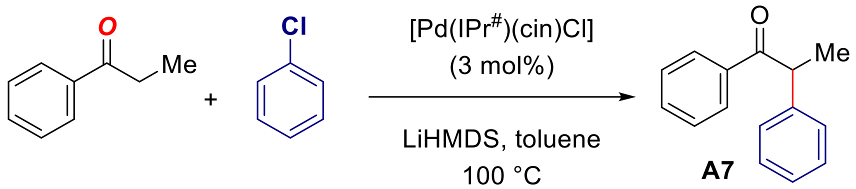

1,2-Diphenylpropan-1-one (A7). An oven dried vial equipped with a stir bar was charged with a ketone substrate (13.4 mg, 0.10 mmol, 1.0 equiv), chlorobenzene (22.4 mg, 0.20 mmol, 2.0 equiv), [(IPr$^{\#}$)Pd(cin)Cl] (4.4 mg, 0.003 mmol, 3 mol%), placed under a positive pressure of argon, and subjected to three evacuation/backfilling cycles under high vacuum. Toluene (0.40 mL, 0.25 M) and LiHMDS (1.0 M in THF, 0.20 mmol, 2.0 equiv) were added with vigorous stirring at room temperature and the reaction mixture was stirred at 100 °C for 24 h. After the indicated time, the reaction mixture was diluted with CH_2Cl_2 (10 mL), washed with water (1 x 10 mL), extracted with CH_2Cl_2 (2 x 10 mL), dried over $MgSO_4$, filtered and concentrated. The sample was analyzed by ^{1}HNMR ($CDCl_3$, 500 MHz) and GC-MS to obtain conversion, selectivity and yield using internal standard and comparison with authentic samples. Purification by chromatography on silica gel (EtOAc/hexanes) afforded the title product. Yield 90% (18.6 mg). ^{1}H NMR (500 MHz, $CDCl_3$) δ 7.98 (d, J = 8.1 Hz, 2H), 7.52-7.46 (m, 1 H), 7.43-7.38 (m, 2 H), 7.36-7.30 (m, 4 H), 7.27-7.18 (m, 1 H), 4.71 (q, J = 6.8 Hz, 1H), 3.88 (d, J = 6.8 Hz, 3H).

^{13}C NMR (126 MHz, $CDCl_3$) δ 200.31, 141.46, 136.49, 132.74, 128.95, 128.75, 128.46, 127.75, 126.87, 47.88, 19.47.

9.9. C–S Cleavage: Carbon–Sulfur Bond Metathesis

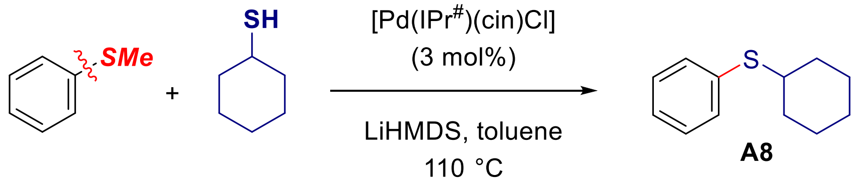

Cyclohexyl(phenyl)sulfane (A8). An oven dried vial equipped with a stir bar was charged with a thioether substrate (12.4 mg, 0.10 mmol, 1.0 equiv), cyclohexanethiol (26.0 mg, 0.20 mmol, 2.0 equiv), [(IPr#)Pd(cin)Cl] (4.4 mg, 0.003 mmol, 3 mol%), placed under a positive pressure of argon, and subjected to three evacuation/backfilling cycles under high vacuum. Toluene (0.10 mL, 1.0 M) and LiHMDS (1.0 M in THF, 0.26 mmol, 2.6 equiv) were added with vigorous stirring at room temperature and the reaction mixture was stirred at 110 °C for 16 h. After the indicated time, the reaction mixture was diluted with CH_2Cl_2 (10 mL), washed with water (1 x 10 mL), extracted with CH_2Cl_2 (2 x 10 mL), dried over $MgSO_4$, filtered and concentrated. The sample was analyzed by ^{1}HNMR ($CDCl_3$, 500 MHz) and GC-MS to obtain conversion, selectivity and yield using internal standard and comparison with authentic samples. Purification by chromatography on silica gel (EtOAc/hexanes) afforded the title product. Yield 80% (18.6 mg). ^1H NMR (500 MHz, $CDCl_3$) δ 7.46-7.40 (m, 2 H), 7.35-7.28 (m, 2 H), 7.26-7.22 (m, 1 H), 3.17-3.10 (m, 1 H), 2.05-2.00 (m, 2 H), 1.83-1.78 (m, 2 H), 1.40-1.29 (m, 6 H). ^{13}C NMR (126 MHz, $CDCl_3$) δ 135.17, 131.85, 128.72, 126.56, 46.57, 33.34, 26.05, 25.76.

9.10. C–H Cleavage: Direct C–H Arylation

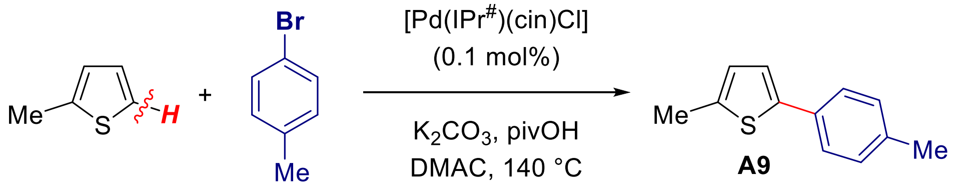

2-Methyl-5-(*p*-tolyl)thiophene (A9). An oven dried vial equipped with a stir bar was charged with a thiophene substrate (9.8 mg, 0.10 mmol, 1.0 equiv), 1-bromo-4-methylbenzene (18.8 mg, 0.11 mmol, 1.1 equiv), potassium carbonate (20.7 mg, 0.15 mmol, 1.5 equiv), PivOH (3.1 mg,

0.03 mmol, 0.30 equiv), [(IPr#)Pd(cin)Cl] (0.15 mg, 0.0001 mmol, 0.1 mol%), placed under a positive pressure of argon, and subjected to three evacuation/backfilling cycles under high vacuum. DMA (0.40 mL, 0.25 M) was added with vigorous stirring at room temperature and the reaction mixture was stirred at 140 °C for 16 h. After the indicated time, the reaction mixture was diluted with CH_2Cl_2 (10 mL), washed with water (1 x 10 mL), extracted with CH_2Cl_2 (2 x 10 mL), dried over $MgSO_4$, filtered and concentrated. The sample was analyzed by ^{1}HNMR ($CDCl_3$, 500 MHz) and GC-MS to obtain conversion, selectivity and yield using internal standard and comparison with authentic samples. Purification by chromatography on silica gel (EtOAc/hexanes) afforded the title product. Yield 95% (17.9 mg). ^{1}H NMR (500 MHz, $CDCl_3$) δ 7.50 (d, *J* = 7.2 Hz, 2H), 7.21 (d, *J* = 7.2 Hz, 2H), 7.11 (d, *J* = 2.1 Hz, 1H), 6.76 (d, *J* = 2.1 Hz, 1H), 2.55 (s, 3H), 2.41 (s, 3H). ^{13}C NMR (126 MHz, $CDCl_3$) δ 142.12, 138.86, 136.75, 131.95, 129.43, 126.04, 125.39, 122.31, 21.09, 15.38.

Details of Crystal Structure Analysis of 9 and 13

Crystallographic information for all of the compounds is given in Table S1 in the Supporting Information (page S21). All compounds were colorless single crystals. Full datasets were collected using graphite-monochromated CuKα radiation (λ = 1.54178 Å) on a Bruker SMART APEX2 single crystal diffractometer. X-rays were provided by a fine-focus sealed X-ray tube operated at 48kV and 30mA. Lattice constants were all determined using the Bruker SAINT software package using all available reflections (after data collection, ORTEP files, see Figures S1-S4).

All data were corrected for absorption by measuring the faces of each crystal and doing a numerical absorption correction. The Bruker software package SHELXTL-2014 was used to solve all of the structures using the direct methods technique and difference electron density maps. All stages of weighted full-matrix least-squares refinement were conducted using F_o^2 data with the same software package. The final structural model for each compound was refined using anisotropic thermal parameters for all non-hydrogen atoms; all of the H atoms were located in difference maps, but were placed in geometrically idealized positions and allowed to "ride" on their parent C, O or N atoms, with bond lengths of 0.95, 1.00, 0.99, 0.98, and 0.84 Å for aromatic, methine, methylene, methyl, and hydroxyl, respectively. The isotropic thermal parameters for these H atoms were fixed to be 1.2 times the U_{iso} for C or N and 1.5 times the U_{iso} for O.

Details for all of the structures are given in Table S1 (page S21). Also included in this table are the largest shifts/s.u. for the final cycle of refinement and the largest maxima and minima in any of the final difference maps.

Table S1. *Crystal Data and Structure Refinement Summaries for **9** and **13**.*

Compound	**9**	**13**
Chemical formula	$C_{93}H_{72}AuClN_2$	$C_{102}H_{81}ClN_2Pd{\cdot}2(CH_2Cl_2)$
M_r	1449.94	1646.40
Crystal system, space group	Monoclinic, *C*2/*c*	Triclinic, *P*1
Temperature (K)	100	100
a, *b*, *c* (Å)	29.1943 (5), 9.7400 (2), 26.3028 (4)	14.037 (1), 15.0707 (11), 21.0521 (15)
β (°)	114.091 (1)	99.161 (4), 95.616 (5), 102.560 (4)
V (Å^3)	6827.8 (2)	4250.6 (5)
Z	4	2
Radiation type	Cu *K*α	Cu *K*α
μ (mm^{-1})	4.80	3.58
Crystal size (mm)	0.34 × 0.23 × 0.14	0.49 × 0.17 × 0.14
Diffractometer	Bruker *SMART* CCD Apex-II area-detector	Bruker *SMART* CCD Apex-II area-detector
Absorption correction	Numerical *SADABS* (Sheldrick, 2008a)	Numerical *SADABS* 2016/2: Krause, L., Herbst-Irmer, R., Sheldrick G.M. & Stalke D., J. Appl. Cryst. 48 (2015) 3-10
T_{min}, T_{max}	0.345, 0.641	0.448, 0.773
No. of measured, independent and observed [$I > 2\sigma(I)$] reflections	31154, 6067, 5996	35885, 13860, 11572
R_{int}	0.032	0.033
$(\sin \theta/\lambda)_{max}$ (Å^{-1})	0.605	0.604
$R[F^2 > 2\sigma(F^2)]$, $wR(F^2)$, S	0.022, 0.057, 1.07	0.057, 0.173, 1.06
No. of reflections	6067	13860
No. of parameters	439	1009
H-atom treatment	H-atom parameters constrained	H-atom parameters constrained
	$w = 1/[\sigma^2(F_o^2) + (0.0256P)^2 + 16.759P]$ where $P = (F_o^2 + 2F_c^2)/3$	$w = 1/[\sigma^2(F_o^2) + (0.0855P)^2 + 13.7136P]$ where $P = (F_o^2 + 2F_c^2)/3$
$\Delta\rho_{max}$, $\Delta\rho_{min}$ (e Å^{-3})	1.11, −0.90	1.49, −1.15

Computer programs: *APEX* 2 (Bruker, 2006), *APEX* 2, *SAINT* (Bruker, 2005), *SHELXL2014*/7 (Sheldrick, 2014), *SHELXTL*.

ORTEP Structures of 9 and 13 (Figures S1-S4)

Figure S1. *ORTEP Structure of **9** (50% ellipsoids).* (Crystallographic data has been deposited with the Cambridge Crystallographic Data Center as supplementary publication no. CCDC 2077050).

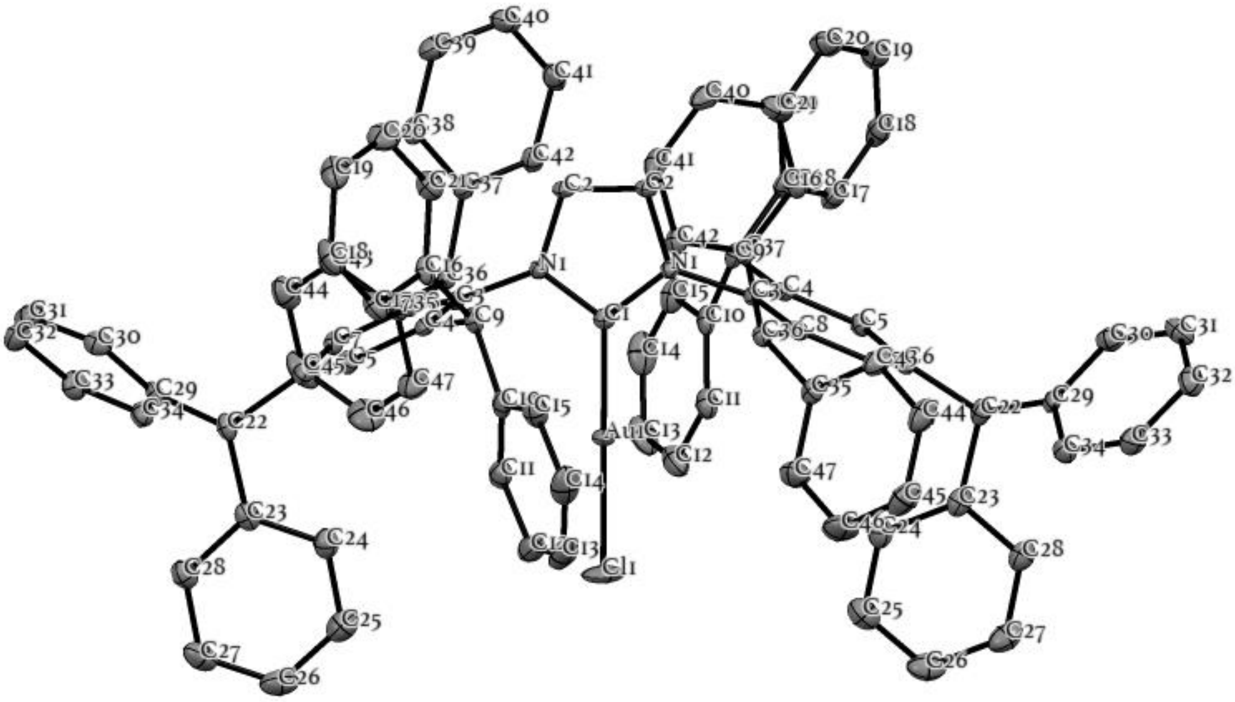

Selected bond lengths (Å) and angles (deg), **9**: Au–C1, 1.972; Au–Cl, 2.2768; C1–N1, 1.356; C3–N1, 1.442(2); C1–Au–Cl, 180.0; N1–C1–N1, 104.9; C3–N1–C1, 122.7; N1–C1–Au, 127.5.

Figure S2. *ORTEP Structure of* ***9*** *(50% ellipsoids).* (Crystallographic data has been deposited with the Cambridge Crystallographic Data Center as supplementary publication no. CCDC 2077050).

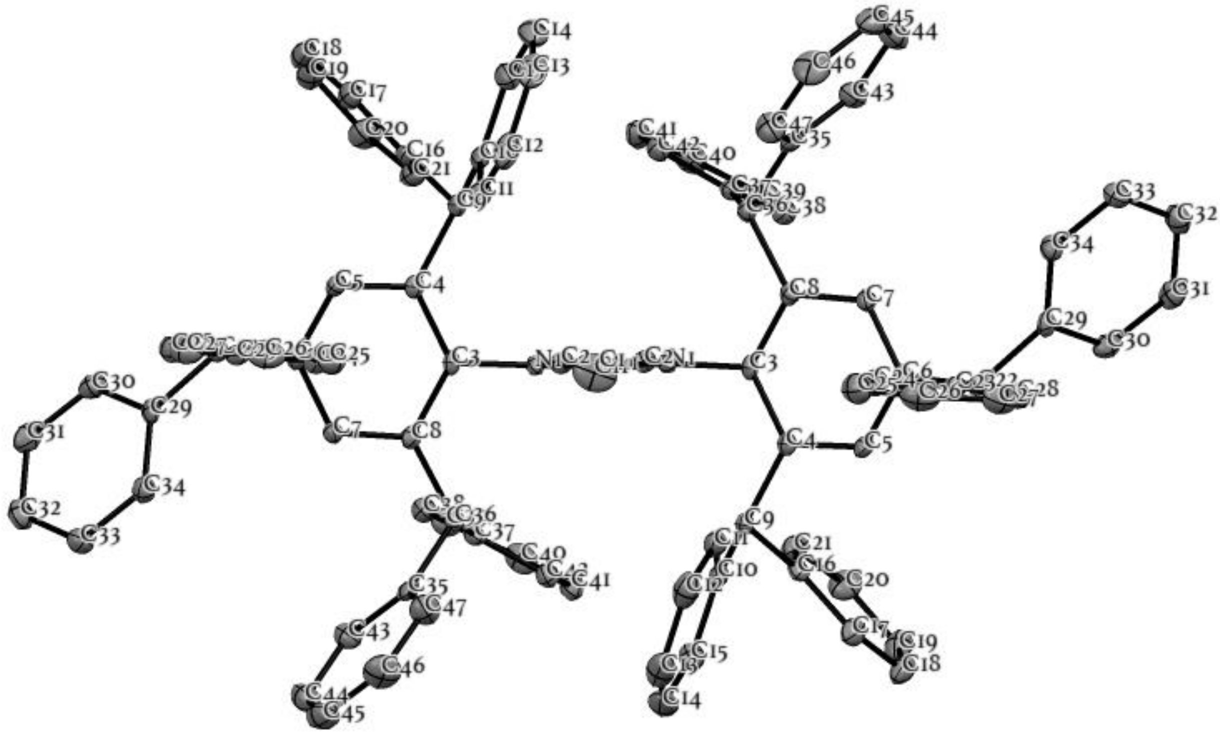

Selected bond lengths (Å) and angles (deg), **9**: Au–C1, 1.972; Au–Cl, 2.2768; C1–N1, 1.356; C3–N1, 1.442(2); C1–Au–Cl, 180.0; N1–C1–N1, 104.9; C3–N1–C1, 122.7; N1–C1–Au, 127.5.

Figure S3. *ORTEP Structure of* ***13*** *(50% ellipsoids)*. (Crystallographic data has been deposited with the Cambridge Crystallographic Data Center as supplementary publication no. CCDC 2077053).

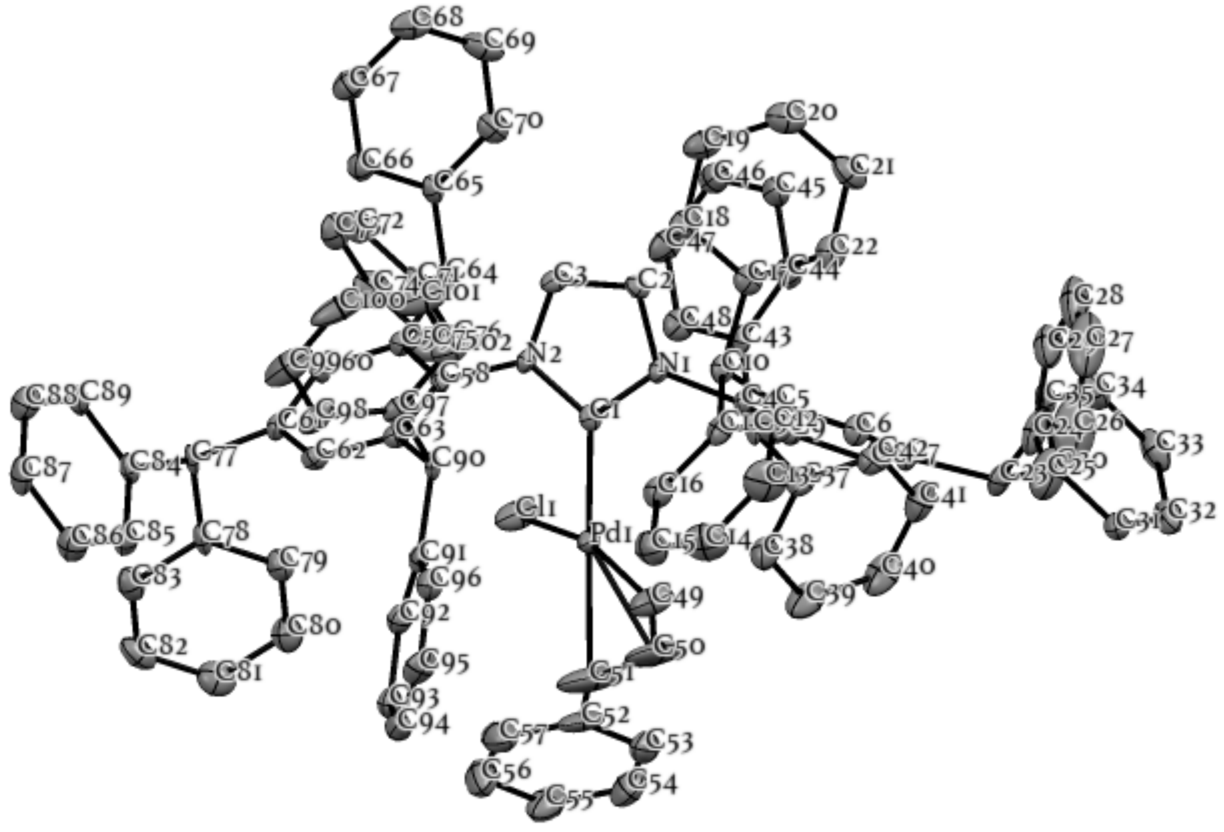

Selected bond lengths (Å) and angles (deg), **13**: Pd–C1, 2.044(4); Pd–Cl, 2.374(1); Pd–C49, 2.121(4); Pd–C50, 2.130(6); Pd–C51, 2.210(7); C1–N1, 1.364(5); C1–N2, 1.368(4); C4–N1, 1.452(4); C58–N2, 1.440(5); C1–Pd–C49, 103.2(2); C1–Pd–C50, 138.5(2); C1–Pd–C51, 169.4(2); C49–Pd–C51, 67.0(2); C1–Pd–Cl, 93.7(1); N1–C1–N2, 103.3(3); C4–N1–C1, 124.9(3); C58–N2–C1, 124.7(3).

Figure S4. *ORTEP Structure of **13** (50% ellipsoids)*. (Crystallographic data has been deposited with the Cambridge Crystallographic Data Center as supplementary publication no. CCDC 2077053).

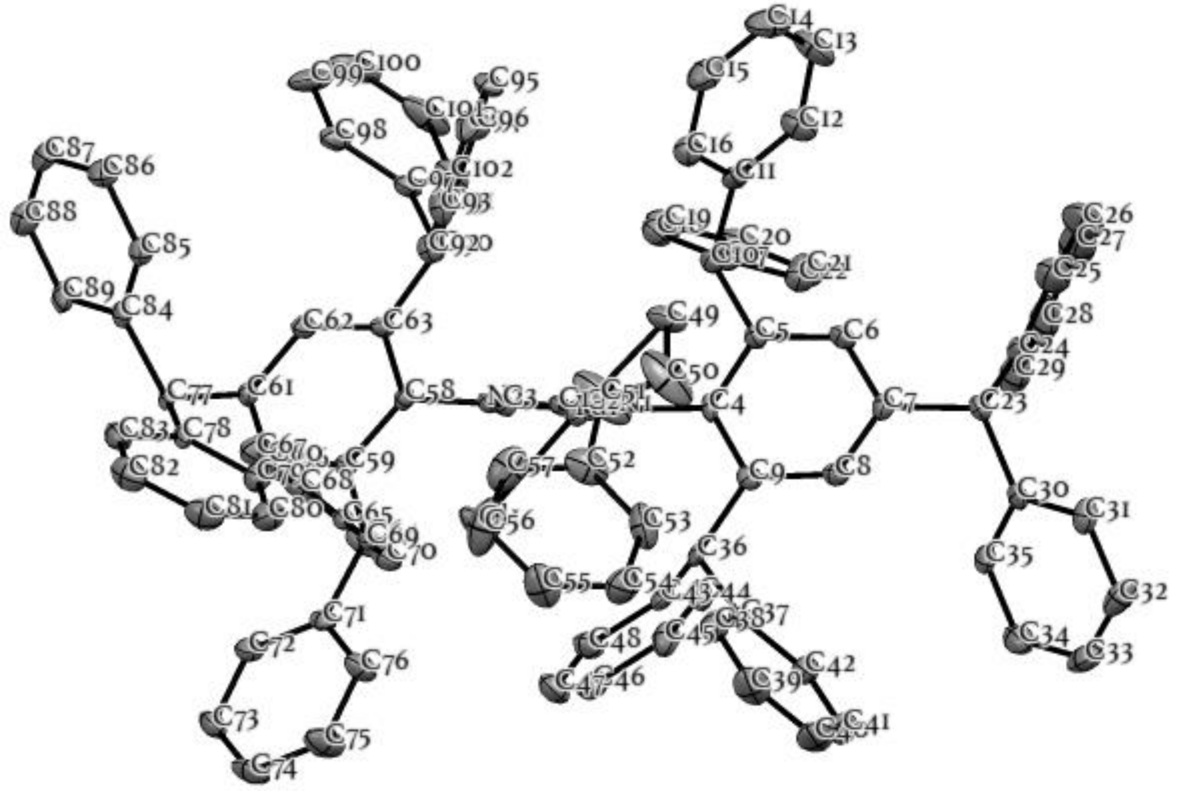

Selected bond lengths (Å) and angles (deg), **13**: Pd–C1, 2.044(4); Pd–Cl, 2.374(1); Pd–C49, 2.121(4); Pd–C50, 2.130(6); Pd–C51, 2.210(7); C1–N1, 1.364(5); C1–N2, 1.368(4); C4–N1, 1.452(4); C58–N2, 1.440(5); C1–Pd–C49, 103.2(2); C1–Pd–C50, 138.5(2); C1–Pd–C51, 169.4(2); C49–Pd–C51, 67.0(2); C1–Pd–Cl, 93.7(1); N1–C1–N2, 103.3(3); C4–N1–C1, 124.9(3); C58–N2–C1, 124.7(3).

Computational Methods

Computational Methods. All of the calculations were performed using Gaussian 09 suite of programs. All of the geometry optimizations were performed at the B3LYP level of theory in the gas phase with the QZVP basis set for palladium (*Phys. Chem. Chem. Phys.* **2005** *7*, 3297) and the 6-311++G(d,p) basis set for the other atoms. This level has been shown to be accurate in predicting structures and electronic properties of NHCs (*Angew. Chem. Int. Ed.* **2018** *57*, 8603). For geometry optimizations, we employed the X-ray structures of 1,3-*bis*-(2,4,6-tribenzhydrylphenyl)-1*H*-imidazol-3-ium, 7,9-*bis*-(2,4,6-tribenzhydrylphenyl)-7*H*-acenaphtho[1,2-*d*]imidazol-9-ium, 1,3-*bis*-(2,4,7-tribenzhydrylnaphthalen-1-yl)-1*H*-imidazol-3-ium or their metal complexes as the starting geometry and performed full optimization. The absence of imaginary frequencies was used to characterize the structures as minima on the potential energy surface. All of the optimized geometries were verified as minima (no imaginary frequencies). Energetic parameters were calculated under standard conditions (298.15 K and 1 atm). Structural representations were generated using CYLview software (Legault, C. Y. CYLview version 1.0 BETA, University of Sherbrooke). All other representations were generated using GaussView (GaussView, version 5, Dennington, R.; Keith, T.; Millam, J. Semichem Inc., Shawnee Mission, KS, 2009) or ChemCraft software (Andrienko, G. L. ChemCraft version b562a, https://www.chemcraftprog.com).

Full Reference for Gaussian 09

Gaussian 09, Revision D.01, Frisch, M. J.; Trucks, G. W.; Schlegel, H. B.; Scuseria, G. E.; Robb, M. A.; Cheeseman, J. R.; Scalmani, G.; Barone, V.; Mennucci, B.; Petersson, G. A.; Nakatsuji, H.; Caricato, M.; Li, X.; Hratchian, H. P.; Izmaylov, A. F.; Bloino, J.; Zheng, G.; Sonnenberg, J. L.; Hada, M.; Ehara, M.; Toyota, K.; Fukuda, R.; Hasegawa, J.; Ishida, M.; Nakajima, T.; Honda, Y.; Kitao, O.; Nakai, H.; Vreven, T.; Montgomery, J. A., Jr.; Peralta, J. E.; Ogliaro, F.; Bearpark, M.; Heyd, J. J.; Brothers, E.; Kudin, K. N.; Staroverov, V. N.; Kobayashi, R.; Normand, J.; Raghavachari, K.; Rendell, A.; Burant, J. C.; Iyengar, S. S.; Tomasi, J.; Cossi, M.; Rega, N.; Millam, M. J.; Klene, M.; Knox, J. E.; Cross, J. B.; Bakken, V.; Adamo, C.; Jaramillo, J.; Gomperts, R.; Stratmann, R. E.; Yazyev, O.; Austin, A. J.; Cammi, R.; Pomelli, C.; Ochterski, J. W.; Martin, R. L.; Morokuma, K.; Zakrzewski, V. G.; Voth, G. A.; Salvador, P.; Dannenberg, J. J.; Dapprich, S.; Daniels, A. D.; Farkas, Ö.; Foresman, J. B.; Ortiz, J. V.; Cioslowski, J.; Fox, D. J. Gaussian, Inc., Wallingford CT, 2009.

Table S2. HOMO and LUMO Energy Levels of IPr-hash and Related NHC Ligands Calculated at the B3LYP 6-311++g(d,p) Level[a,b]

entry	compound	orbital	E [au]	E [eV]	E [kcal/mol]	ΔE [kcal/mol]
1	IPr#	HOMO	-0.2264	-6.16	-142.07	
2	IPr#	LUMO	-0.0354	-0.96	-22.21	-5.20
3[a]	BIAN-IPr#	HOMO-1	-0.2263	-6.16	-142.00	
4[b]	BIAN-IPr#	LUMO+1	-0.0349	-0.95	-21.90	-5.21
5[a]	Np# (rac)	HOMO-2	-0.2259	-6.15	-141.75	
6[b]	Np# (rac)	LUMO+2	-0.0356	-0.97	-22.34	-5.18
7[a]	Np# (meso)	HOMO-2	-0.2253	-6.13	-141.38	
8[b]	Np# (meso)	LUMO+2	-0.0347	-0.94	-21.77	-5.19
9	IPr*	HOMO	-0.2249	-6.12	-141.12	
10	IPr*	LUMO	-0.0330	-0.90	-20.71	-5.22
11	IPr	HOMO	-0.2210	-6.01	-139.78	
12[b]	IPr	LUMO+1	-0.0177	-0.48	-12.21	-5.53
13	IMes	HOMO	-0.2169	-5.90	-137.21	
14[b]	IMes	LUMO+2	-0.0121	-0.33	-8.70	-5.57

[a]HOMO-1/HOMO-2 due to required in-plane σ-orbital. [b]LUMO+1/LUMO+2 due to required orbital symmetry. See, Falivene, L.; Cavallo, L. *Coord. Chem. Rev.* **2017**, *344*, 101-114.

Figure S5. Optimized Geometries of NHCs Referred to from the Main Manuscript at the B3LYP 6-311++g(d,p) Level

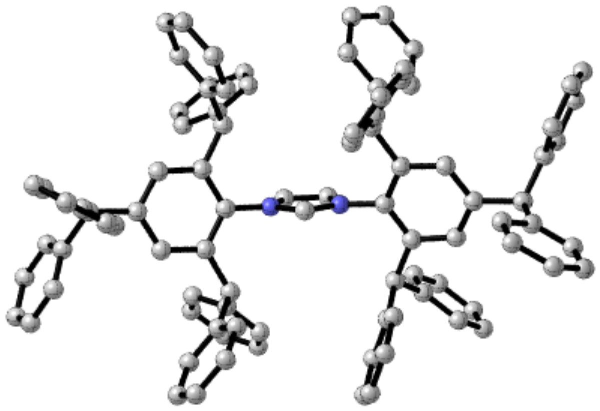

IPr#

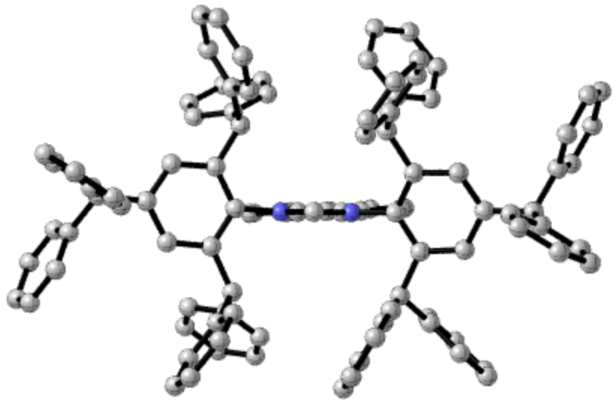

BIAN-IPr#

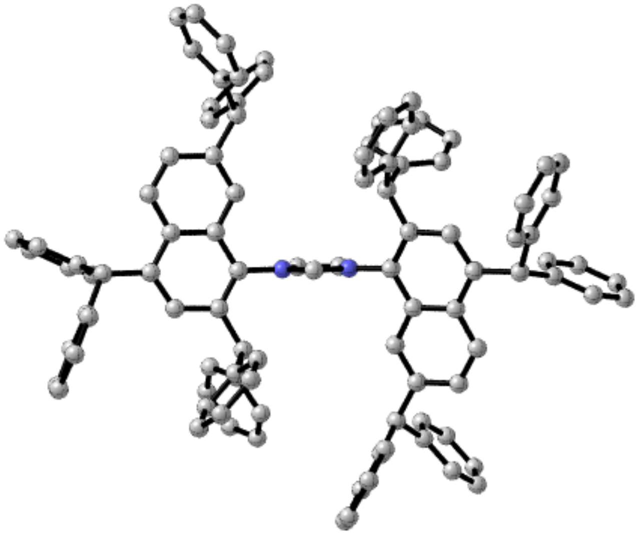

Np# (rac)

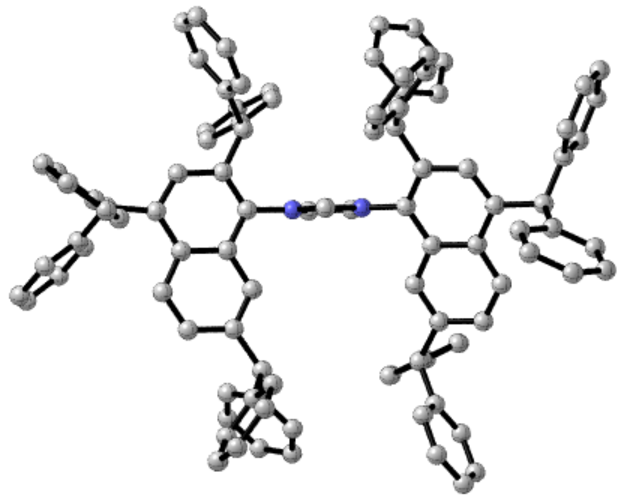

Np# (meso)

Figure S5 cont. Optimized Geometries of NHCs Referred to from the Main Manuscript at the B3LYP 6-311++g(d,p) Level

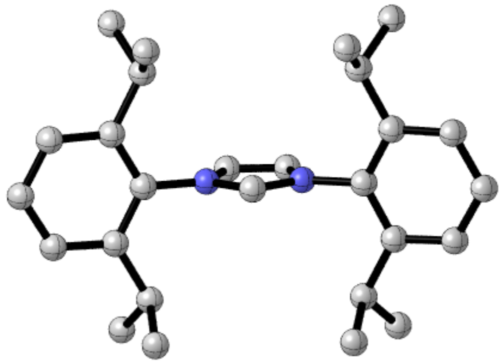

IPr

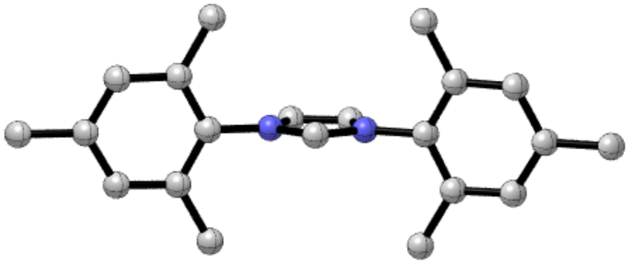

IMes

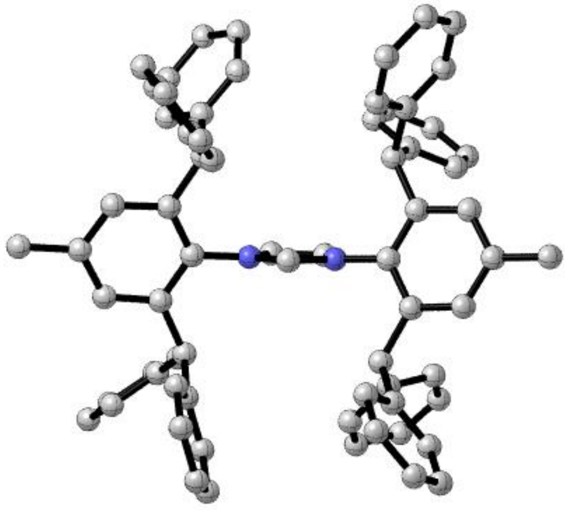

IPr*

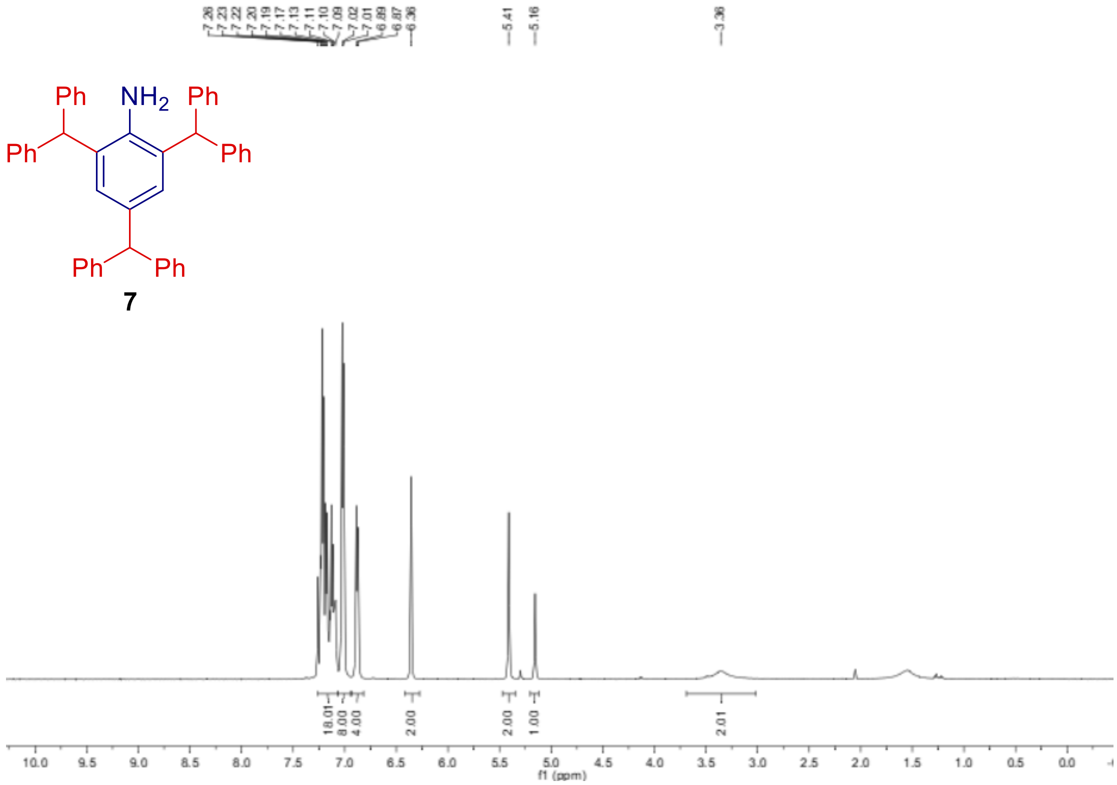

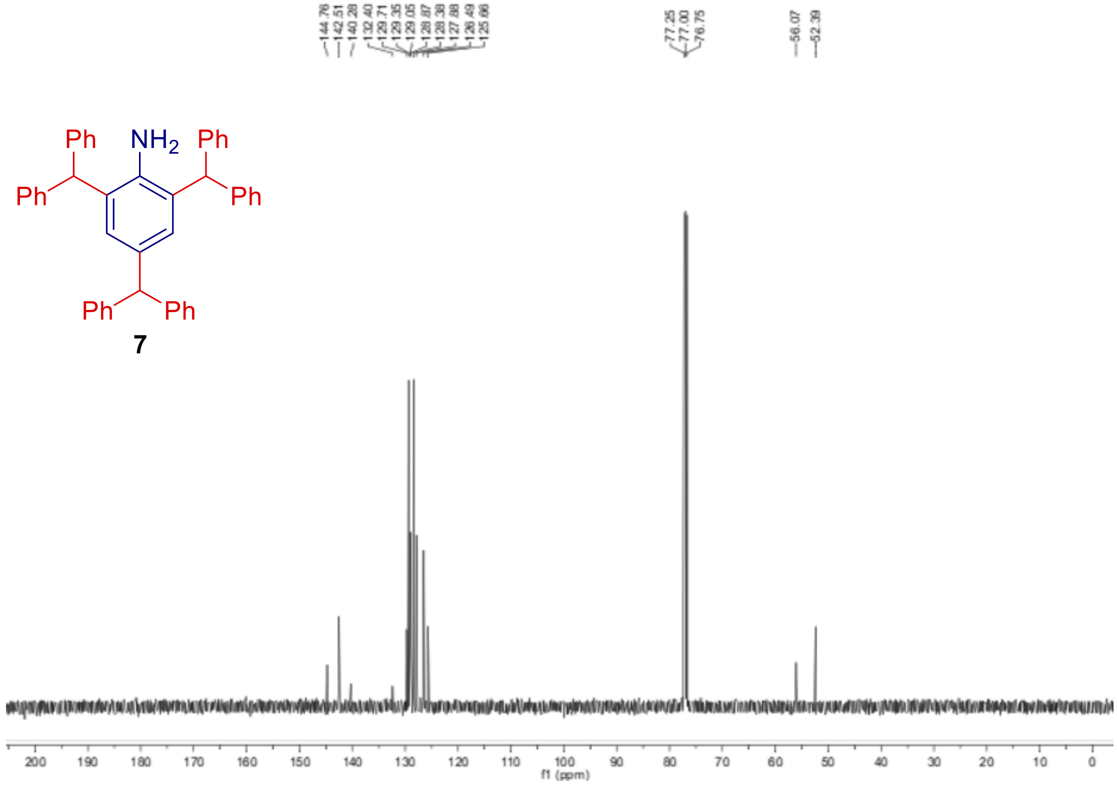

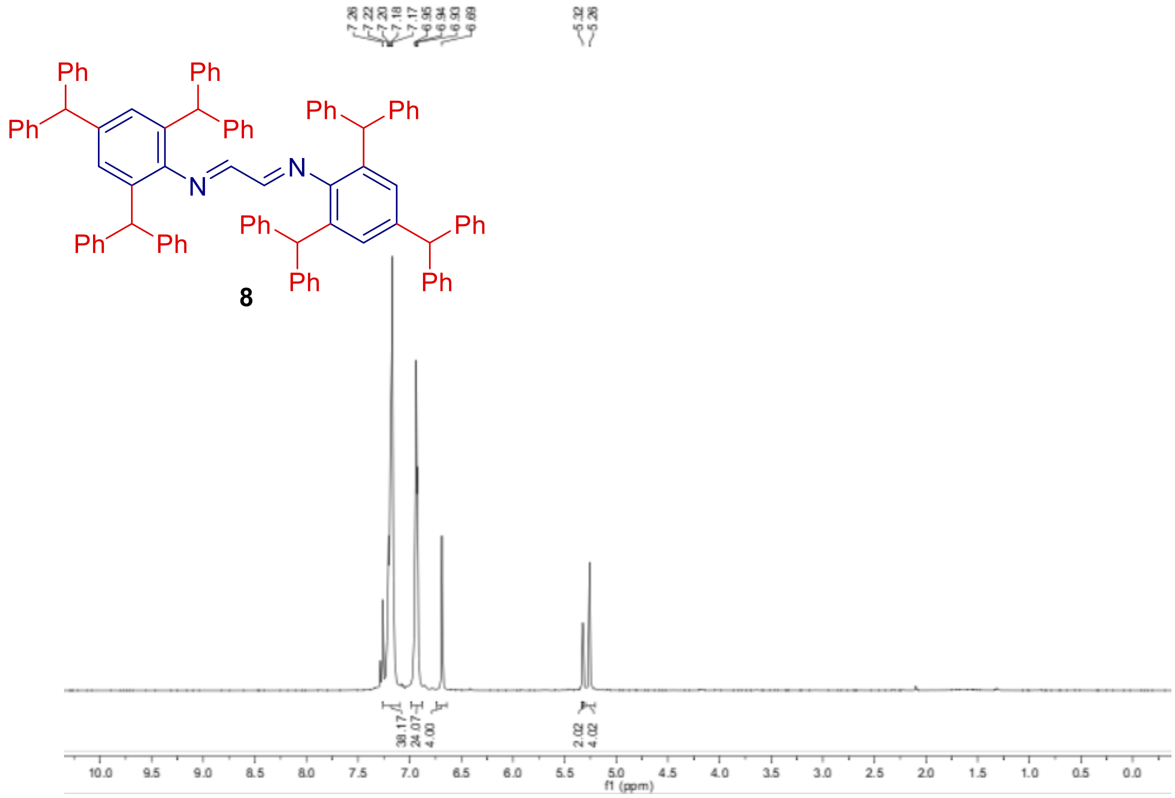
7.26
7.22
7.20
7.18
7.17
6.95
6.94
6.93
6.69
5.32
5.26
Ph
N
8
38.17
24.07
4.00
2.02
4.02
f1 (ppm)

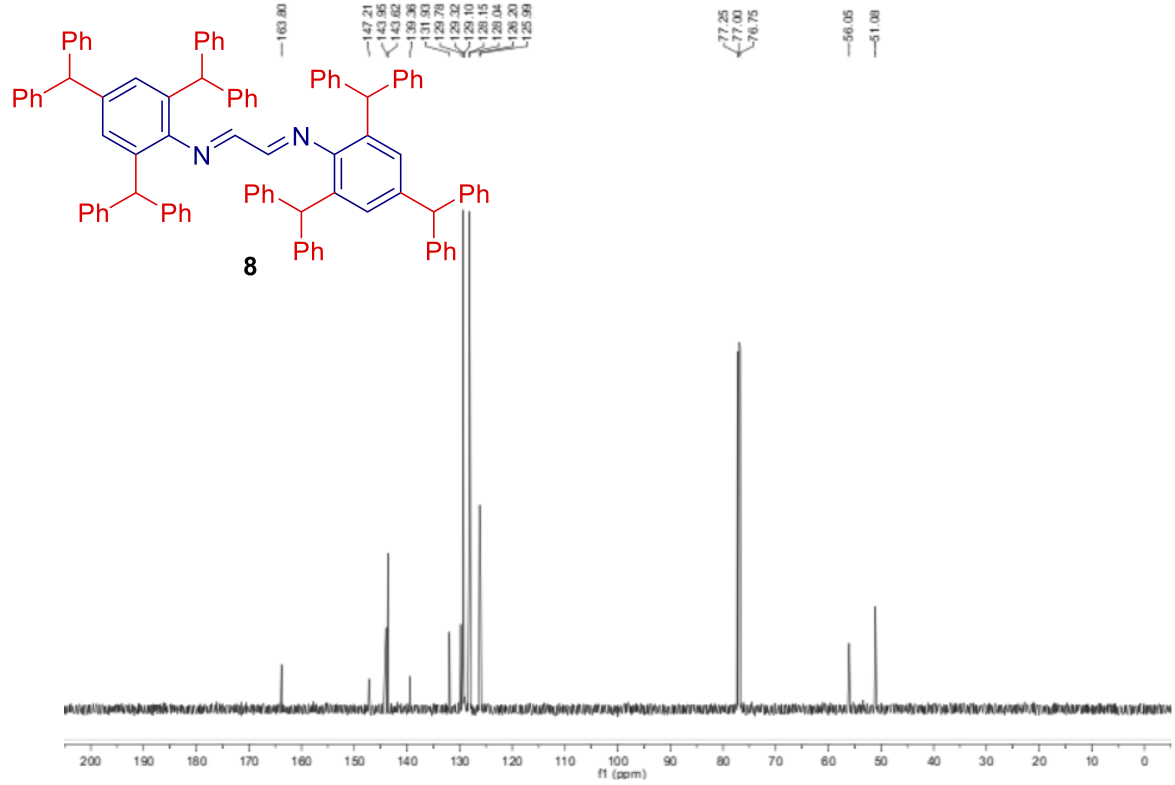
163.80
147.21
143.95
143.62
139.36
131.93
129.78
129.32
129.10
128.15
128.04
126.20
125.99
77.25
77.00
76.75
56.05
51.08
Ph
N
8
f1 (ppm)

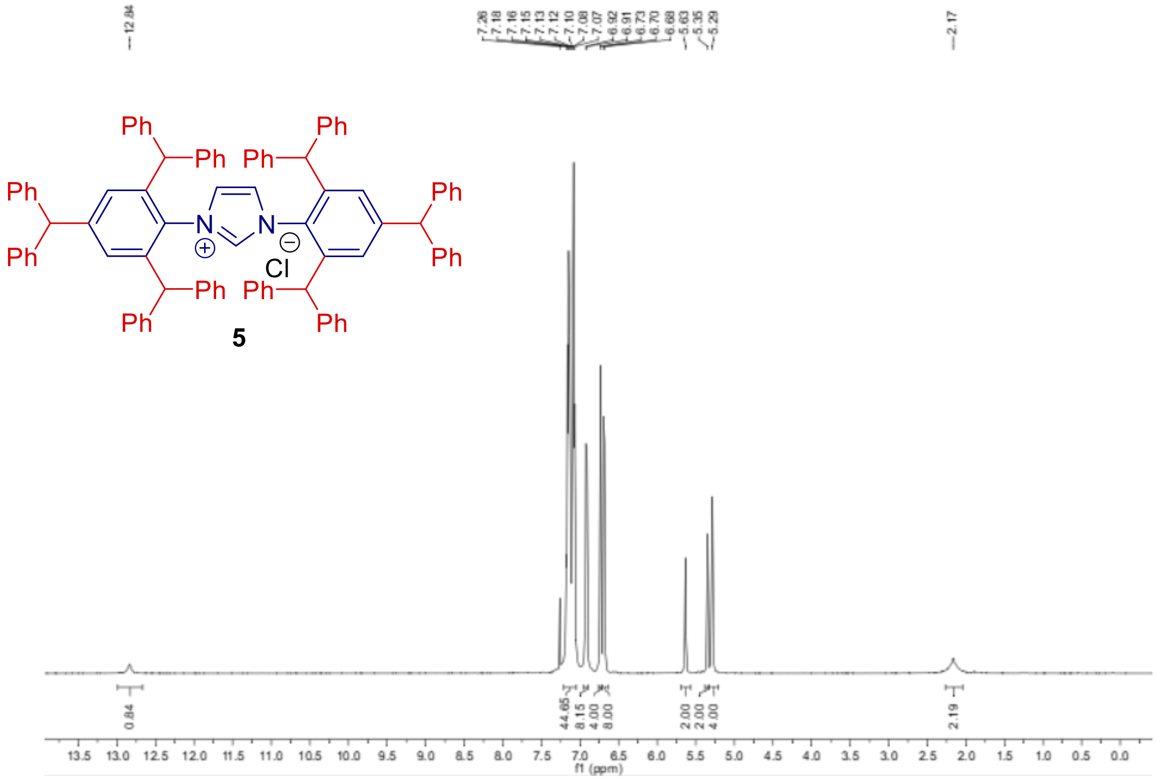

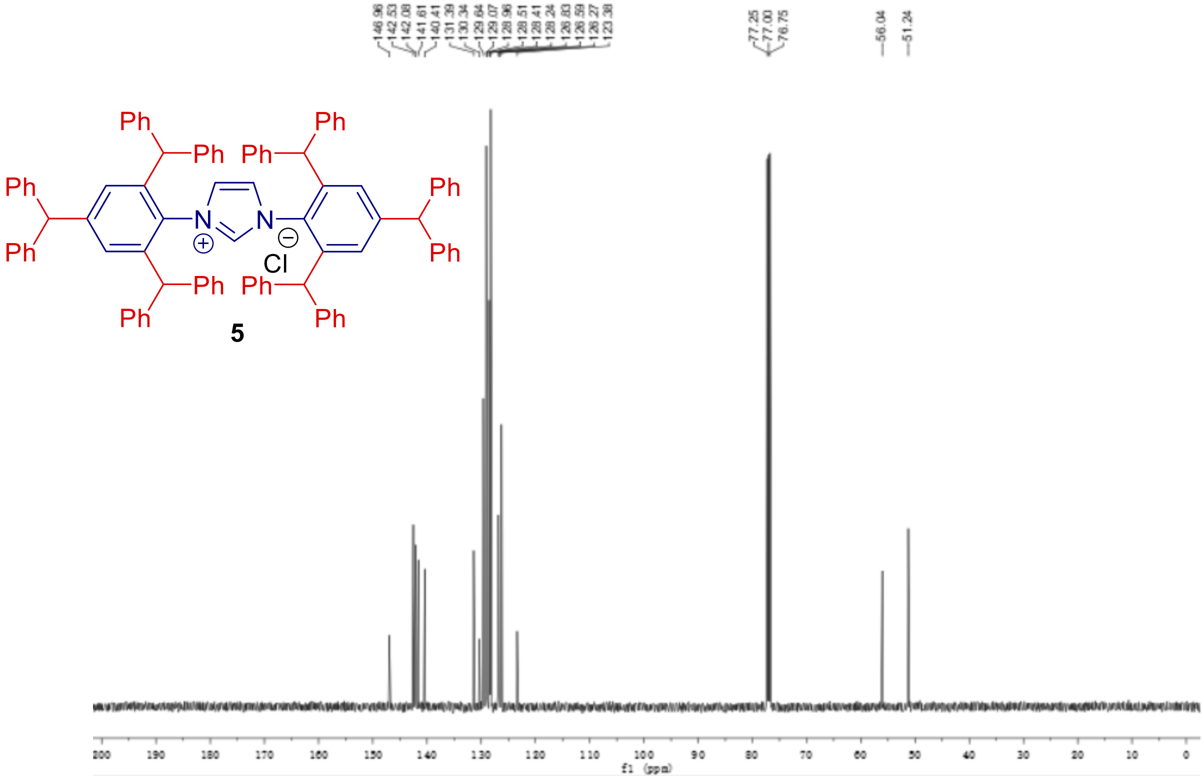

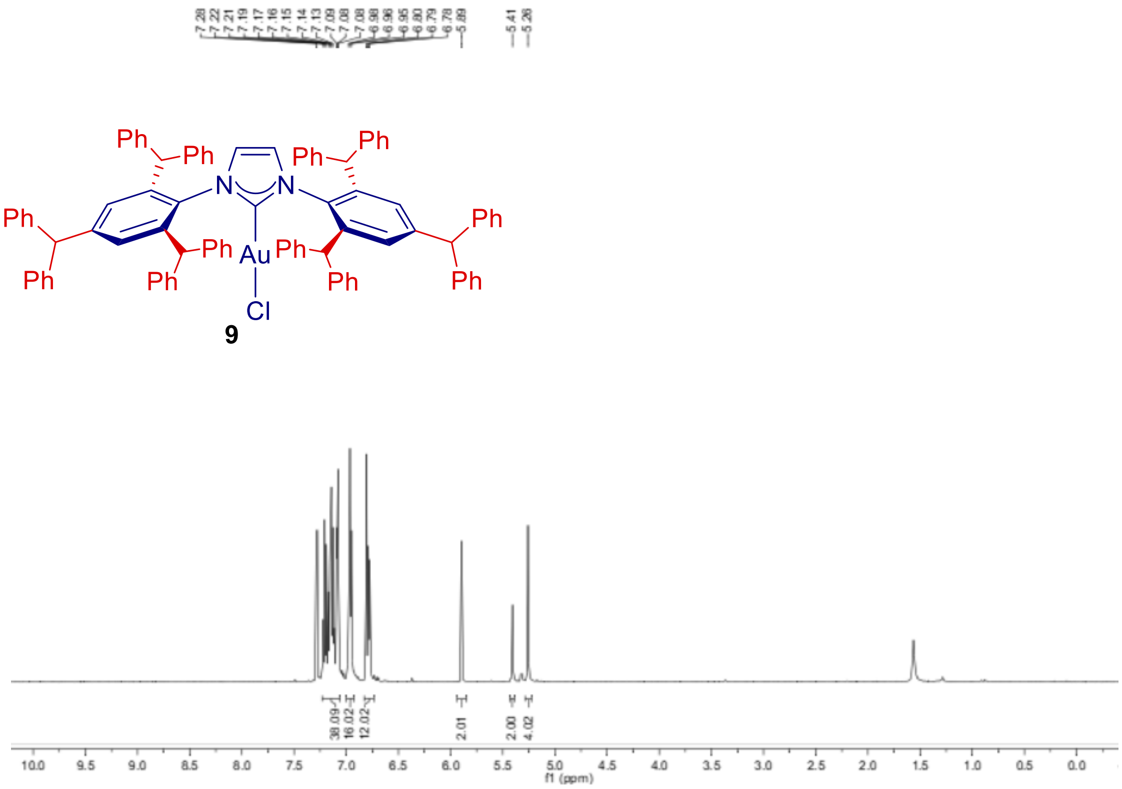

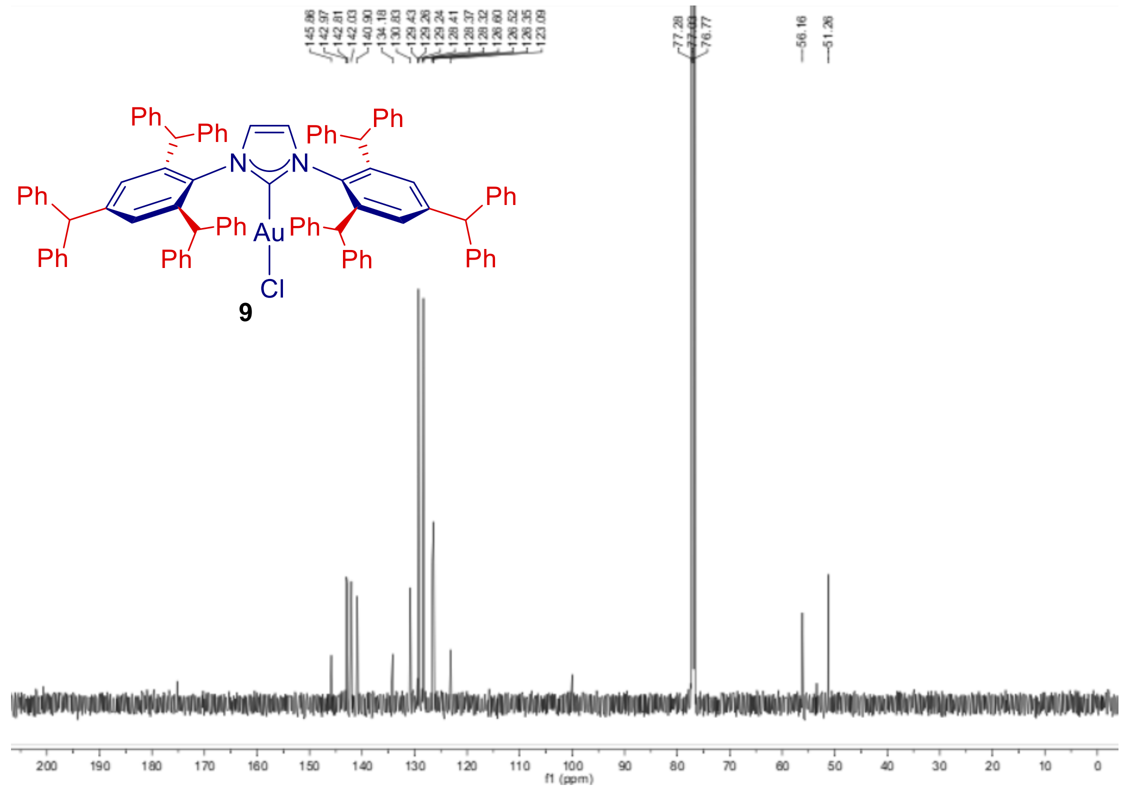

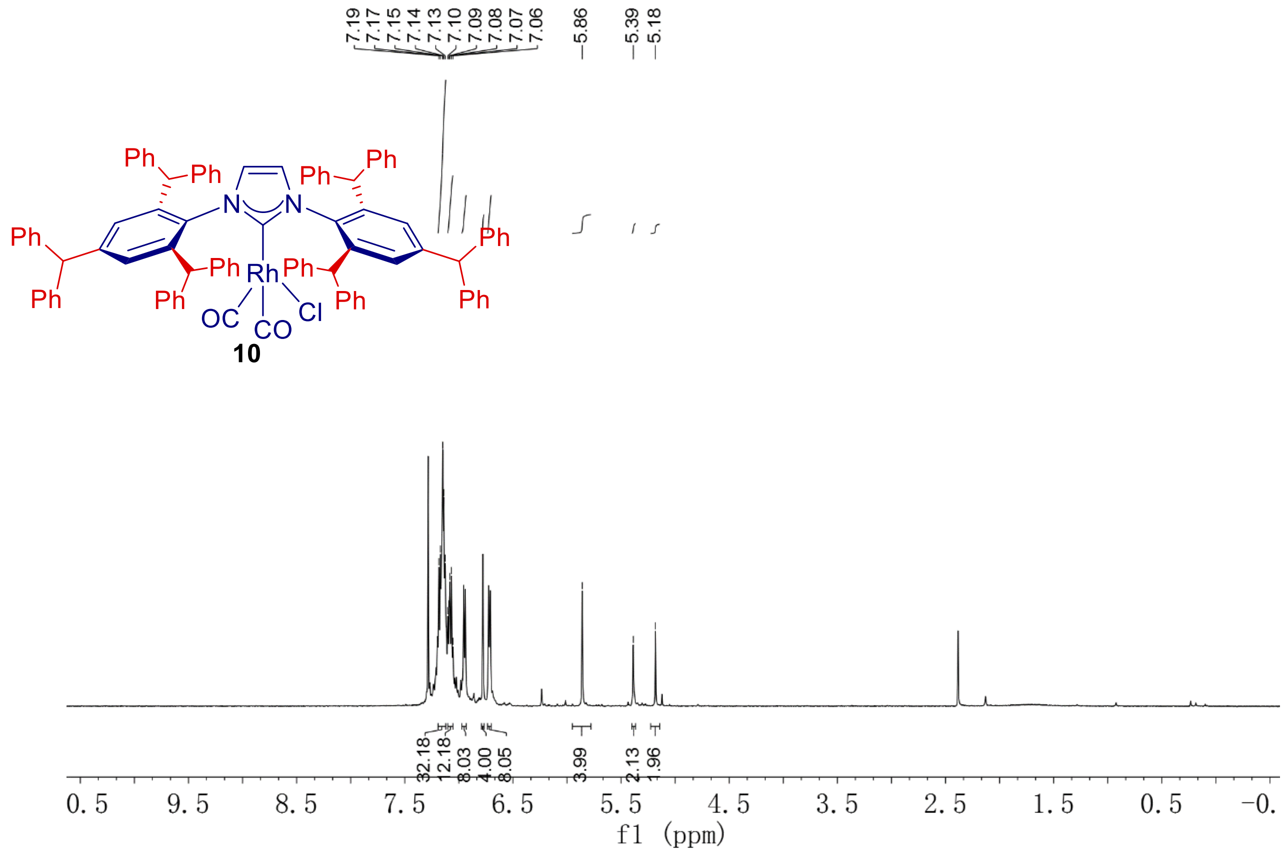

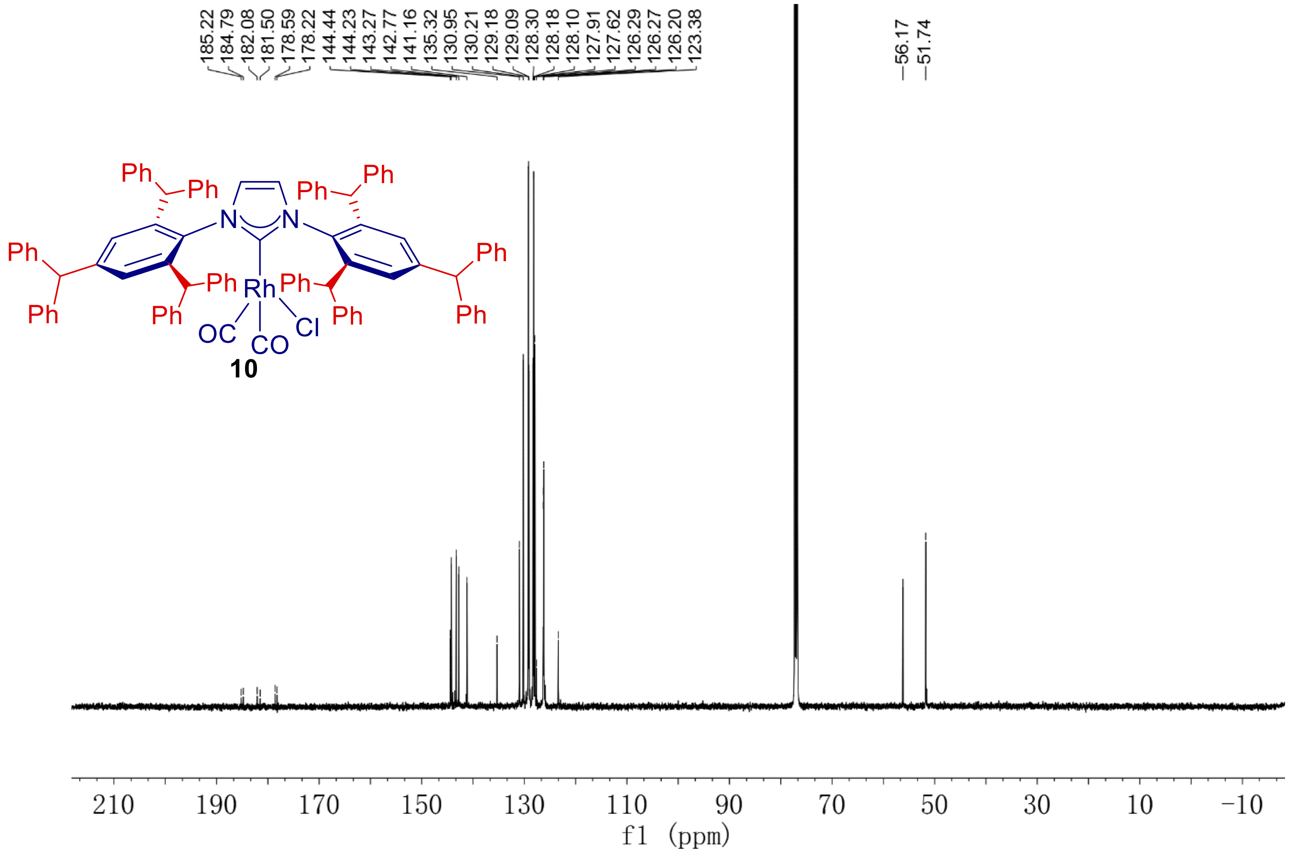

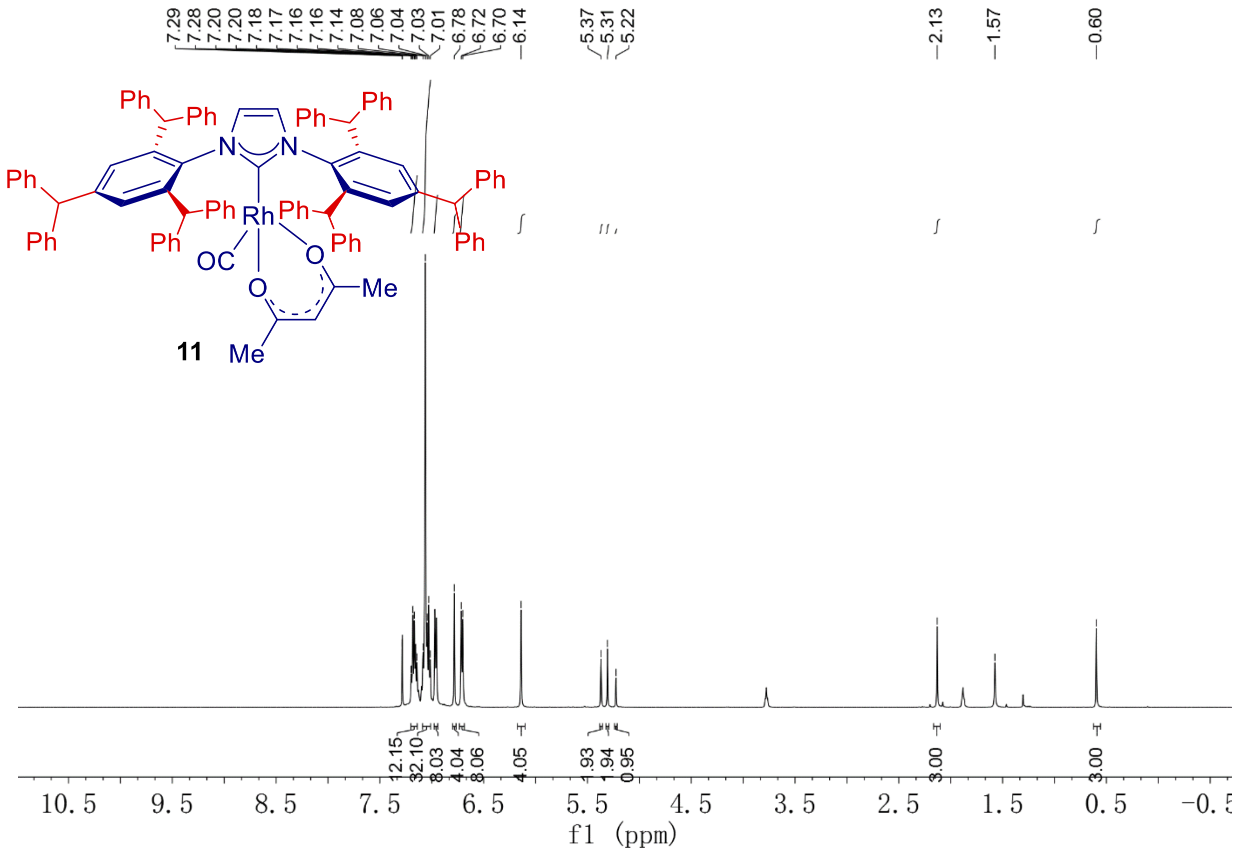
7.29
7.28
7.20
7.20
7.18
7.17
7.16
7.16
7.14
7.08
7.06
7.04
7.03
7.01
6.78
6.72
6.70
6.14
5.37
5.31
5.22
2.13
1.57
0.60
11
12.15
32.10
8.03
4.04
8.06
4.05
1.93
1.94
0.95
3.00
3.00
f1 (ppm)

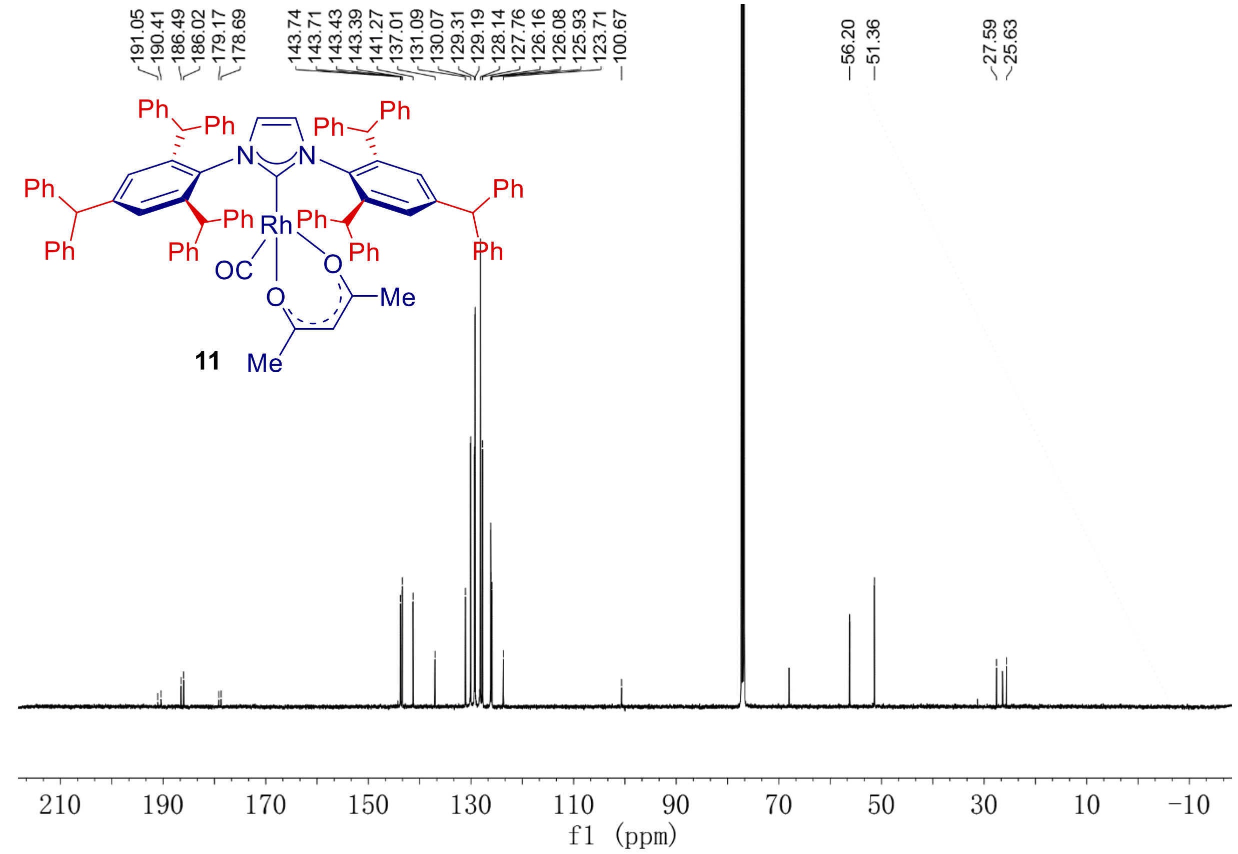
191.05
190.41
186.49
186.02
179.17
178.69
143.74
143.71
143.43
143.39
141.27
137.01
131.09
130.07
129.31
129.19
128.14
127.76
126.16
126.08
125.93
123.71
100.67
56.20
51.36
27.59
25.63
11
f1 (ppm)

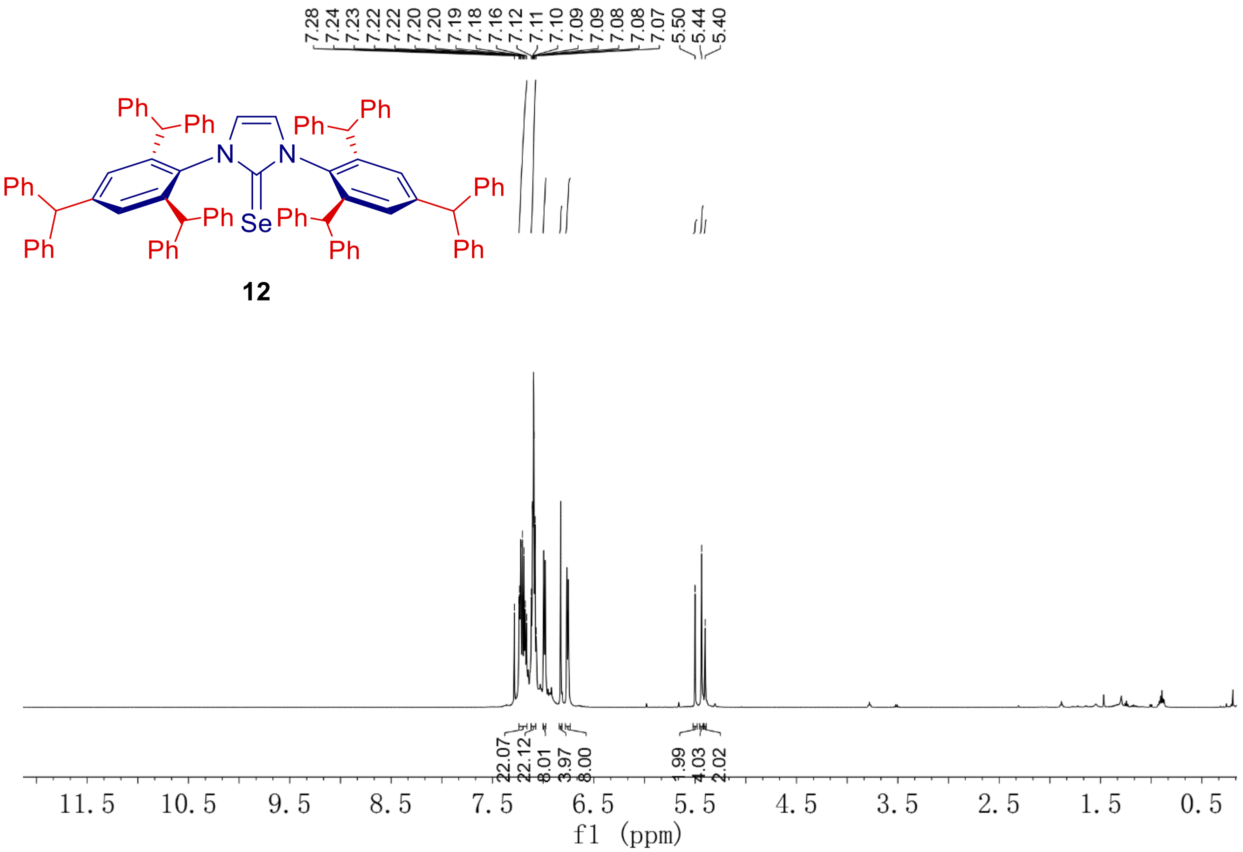
7.28
7.24
7.23
7.22
7.22
7.20
7.20
7.19
7.18
7.16
7.12
7.11
7.10
7.09
7.09
7.08
7.08
7.07
5.50
5.44
5.40
Ph
N
N
Se
12
22.07
22.12
8.01
3.97
8.00
1.99
4.03
2.02
11.5 10.5 9.5 8.5 7.5 6.5 5.5 4.5 3.5 2.5 1.5 0.5
f1 (ppm)

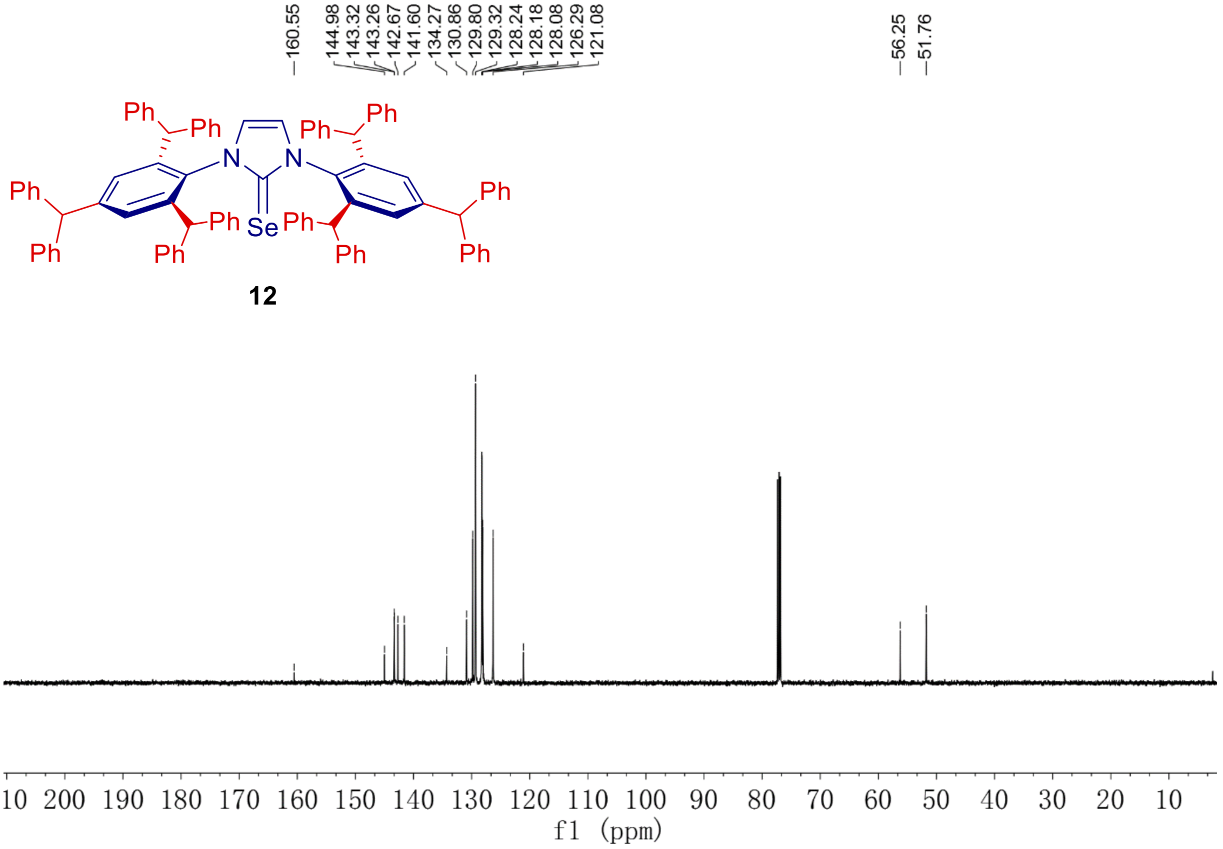
160.55
144.98
143.32
143.26
142.67
141.60
134.27
130.86
129.80
129.32
128.24
128.18
128.08
126.29
121.08
56.25
51.76
Ph
N
N
Se
12
10 200 190 180 170 160 150 140 130 120 110 100 90 80 70 60 50 40 30 20 10
f1 (ppm)

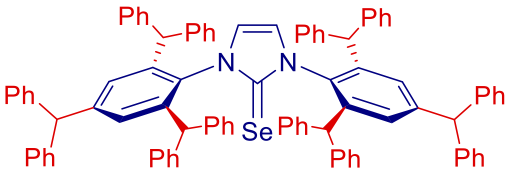
Ph
Ph
Ph
Ph
Ph
Ph
Ph
Ph
Ph
Ph
Ph
Ph
N
N
Se

12

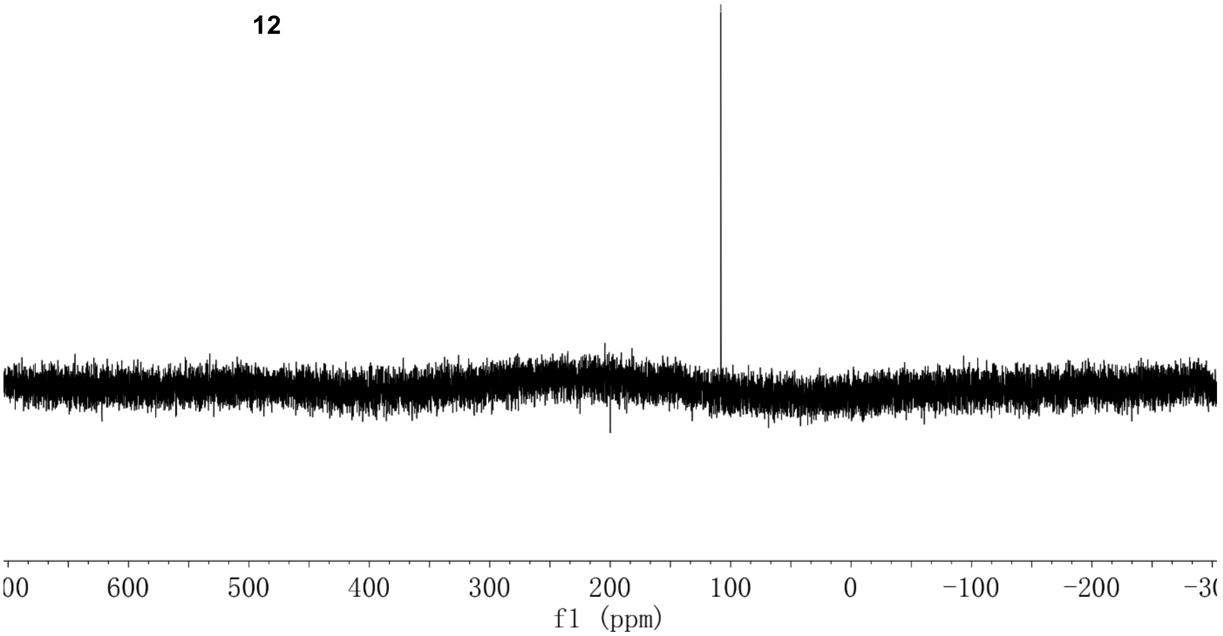
—108.11
00
600
500
400
300
200
100
0
−100
−200
−3(
f1 (ppm)

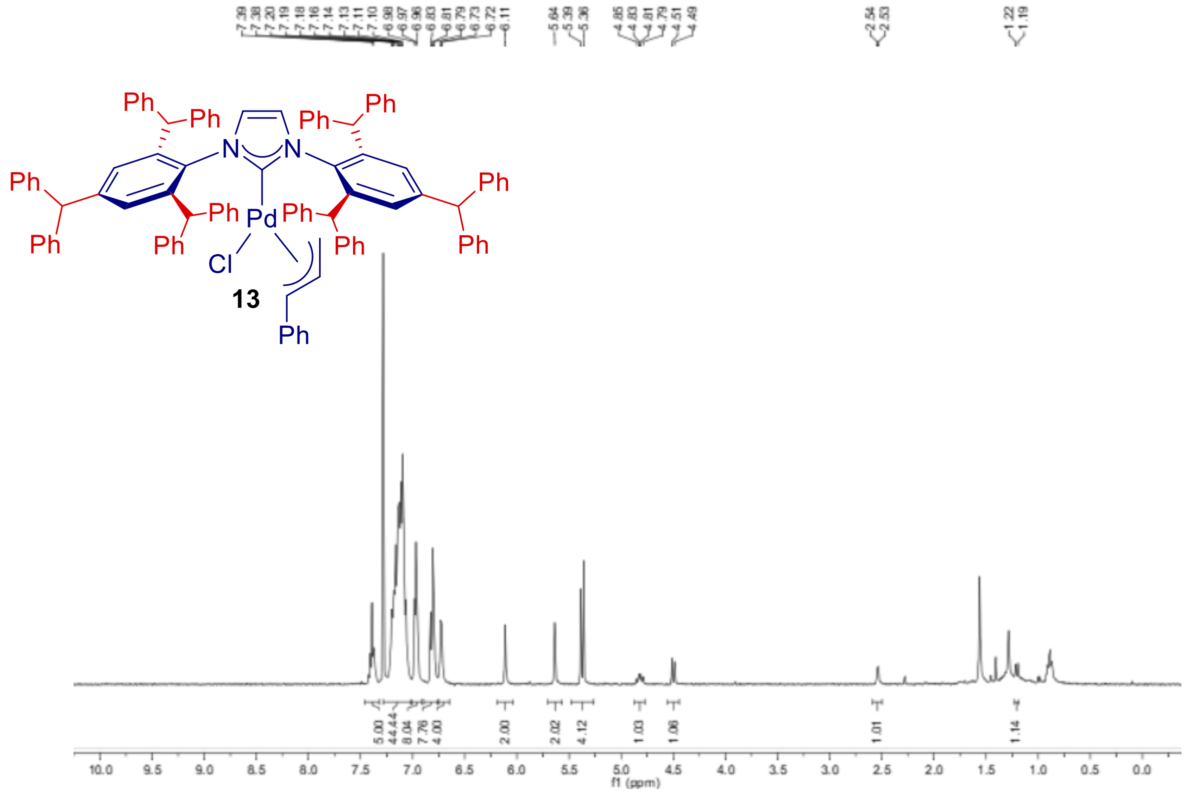

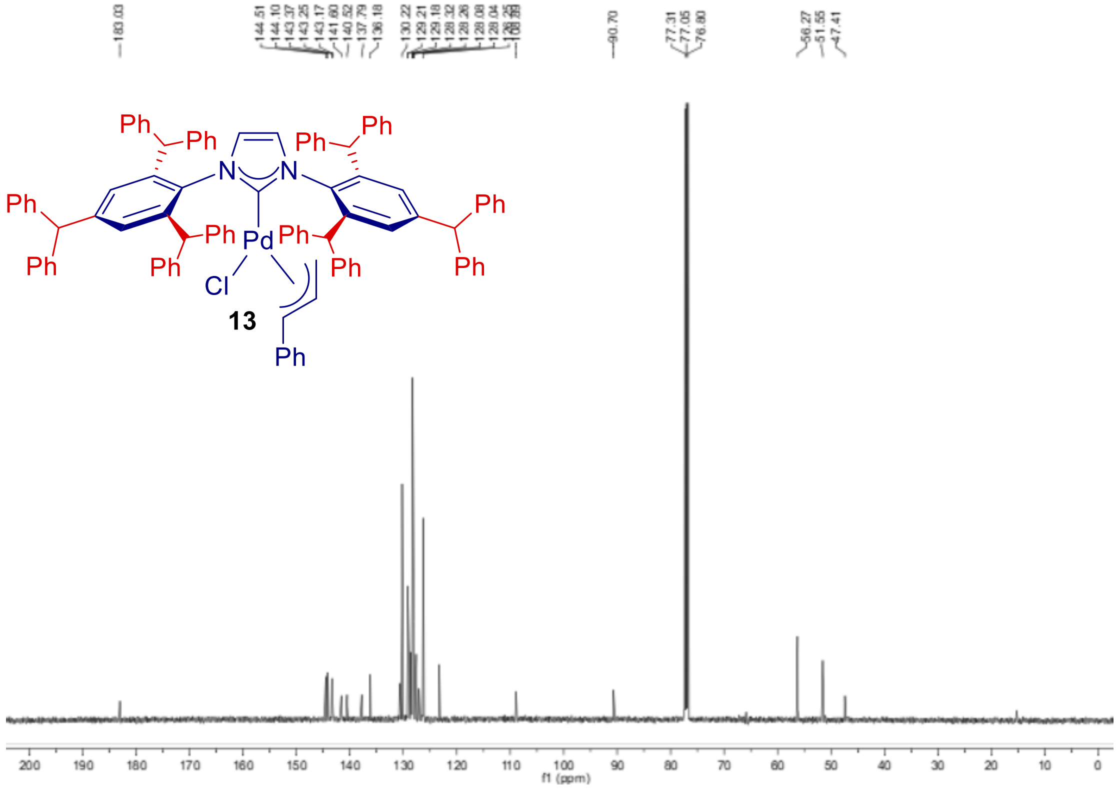

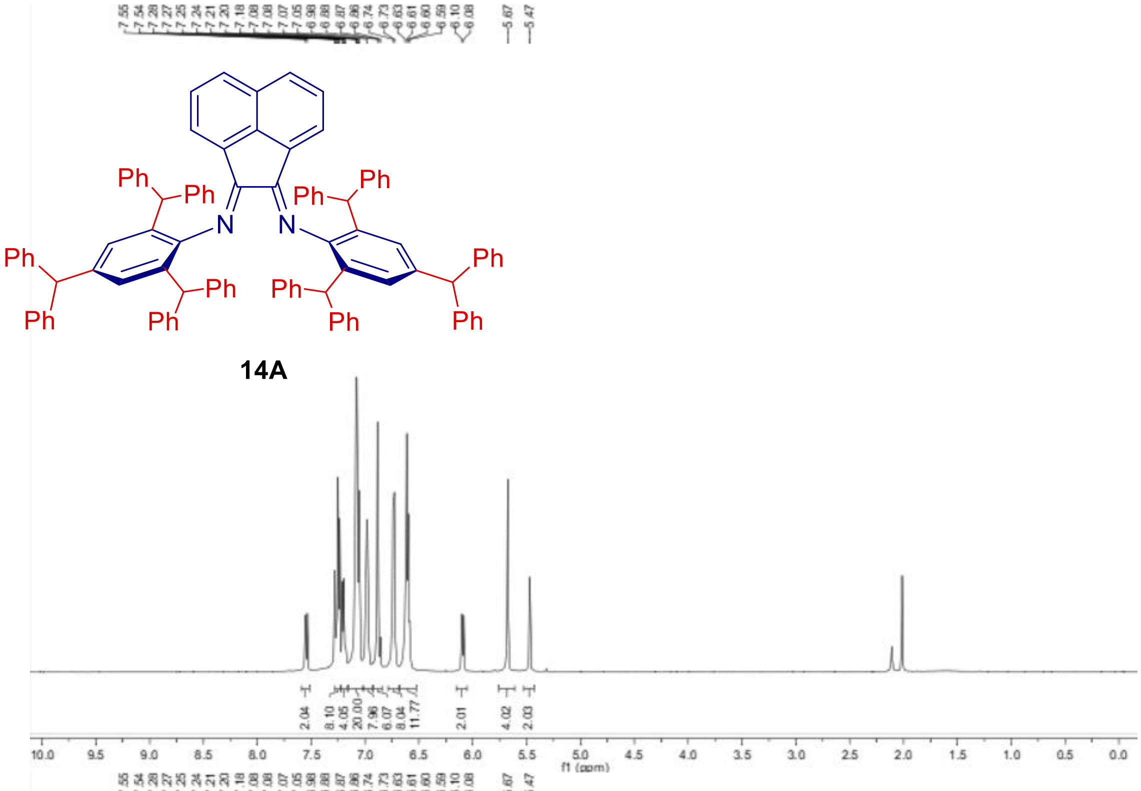
14A
f1 (ppm)

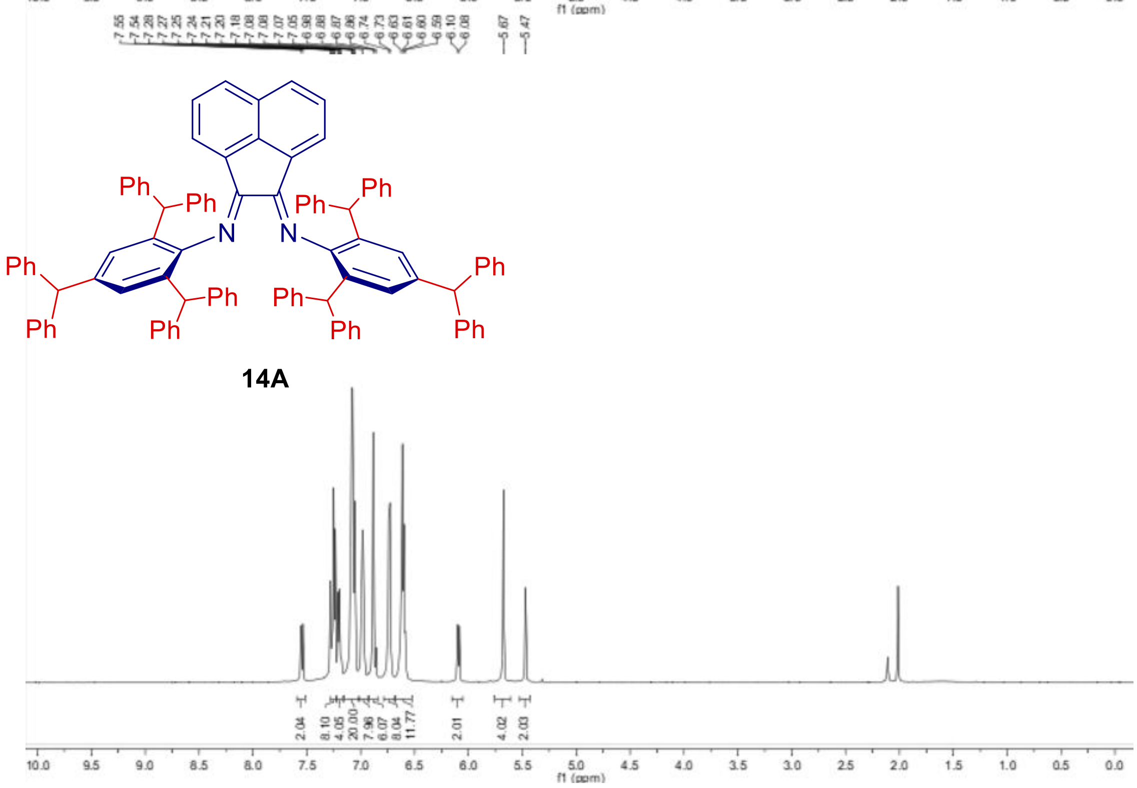
14A
f1 (ppm)

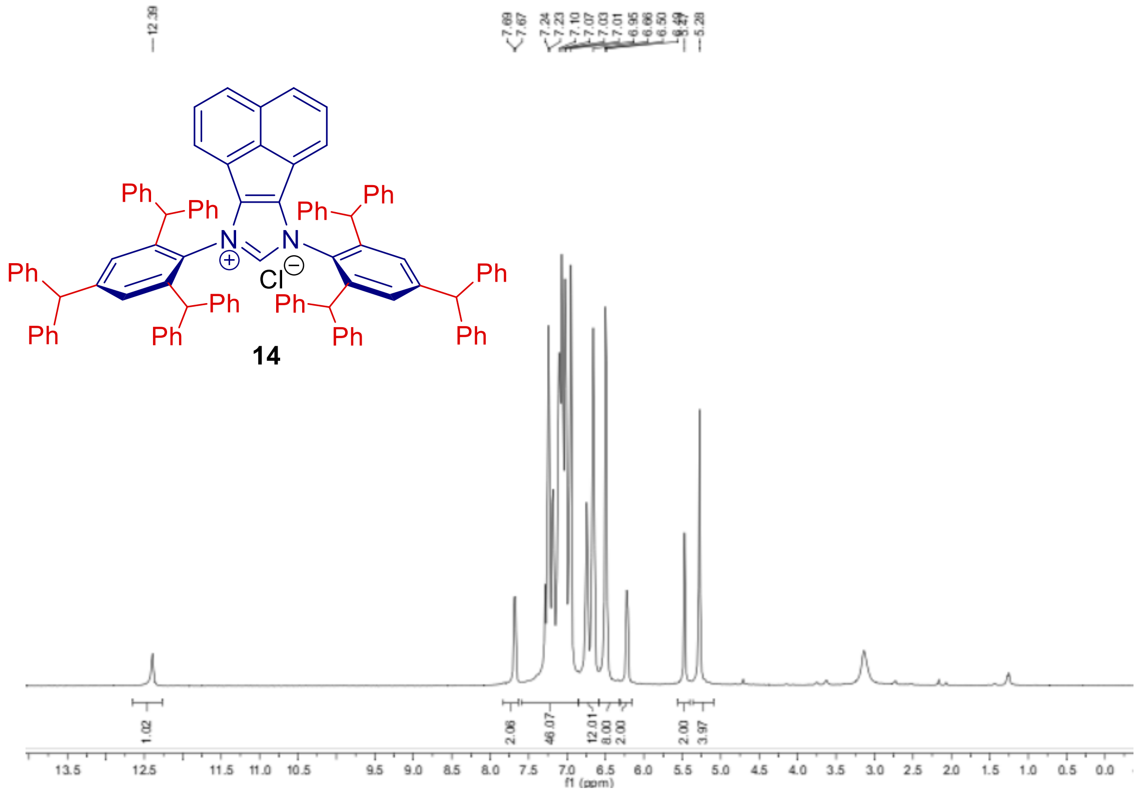
14
f1 (ppm)

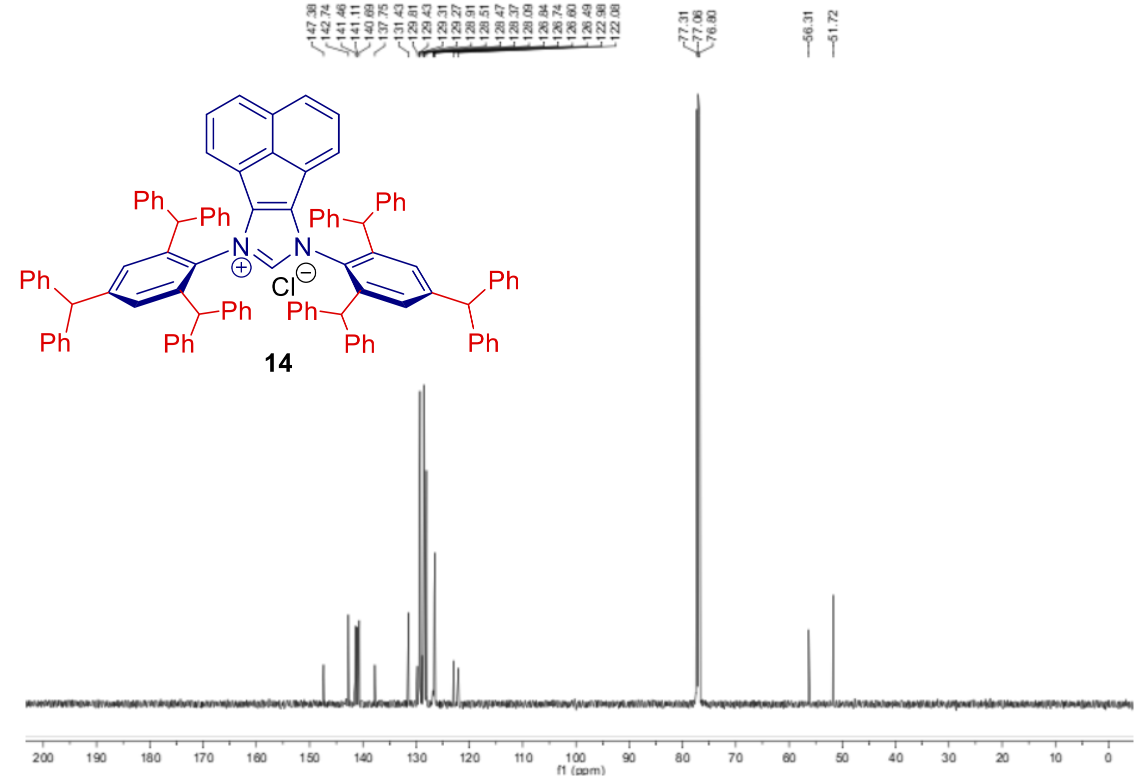
14
f1 (ppm)

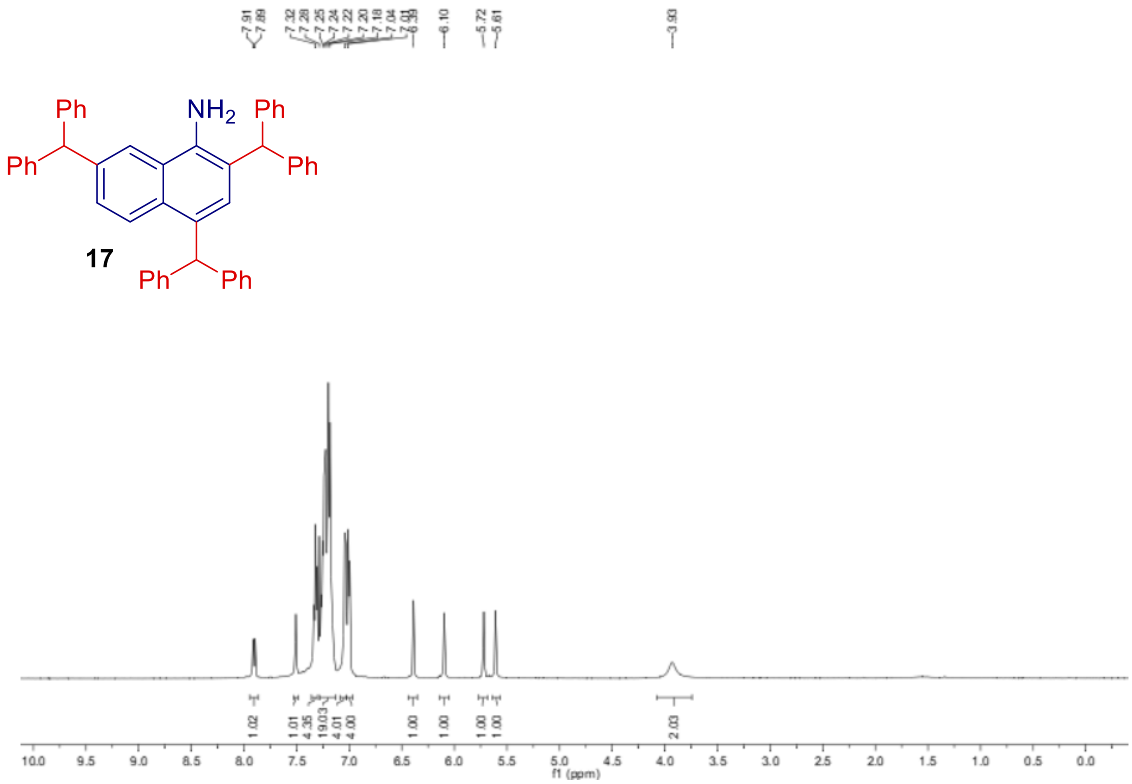
Ph
NH2
Ph
Ph
Ph
17
Ph
Ph
f1 (ppm)

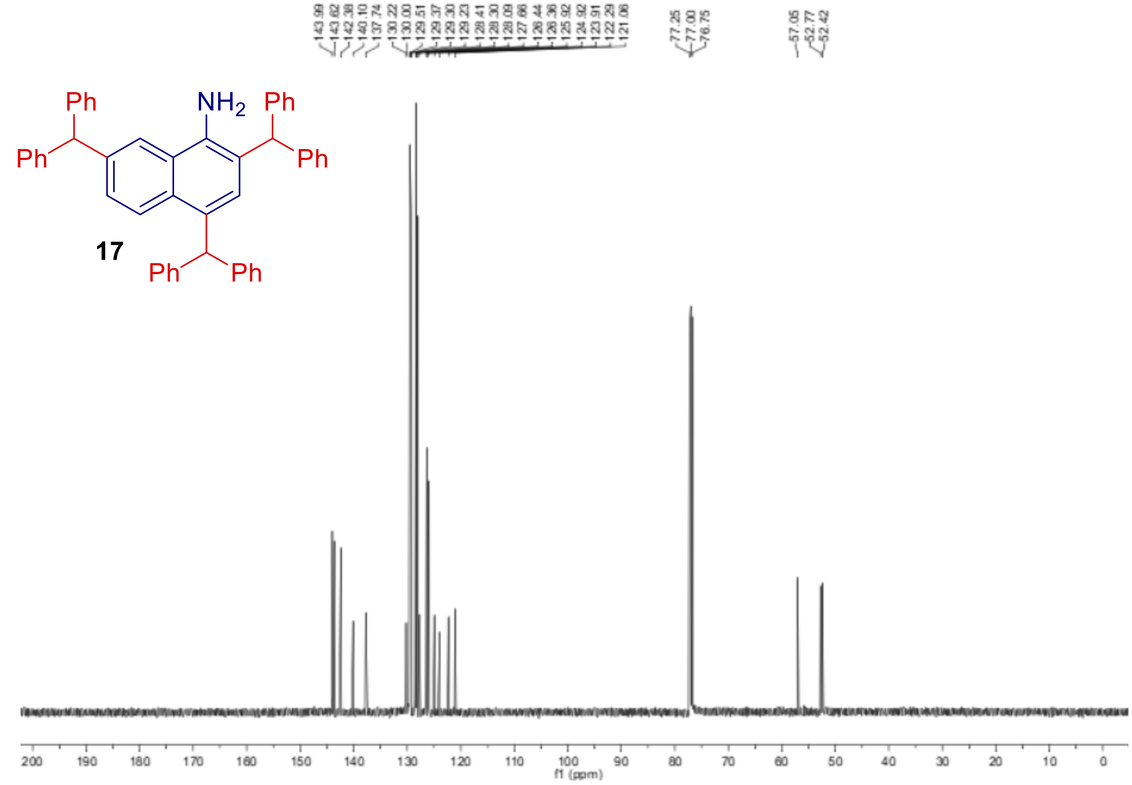
Ph
NH2
Ph
Ph
Ph
17
Ph
Ph
f1 (ppm)

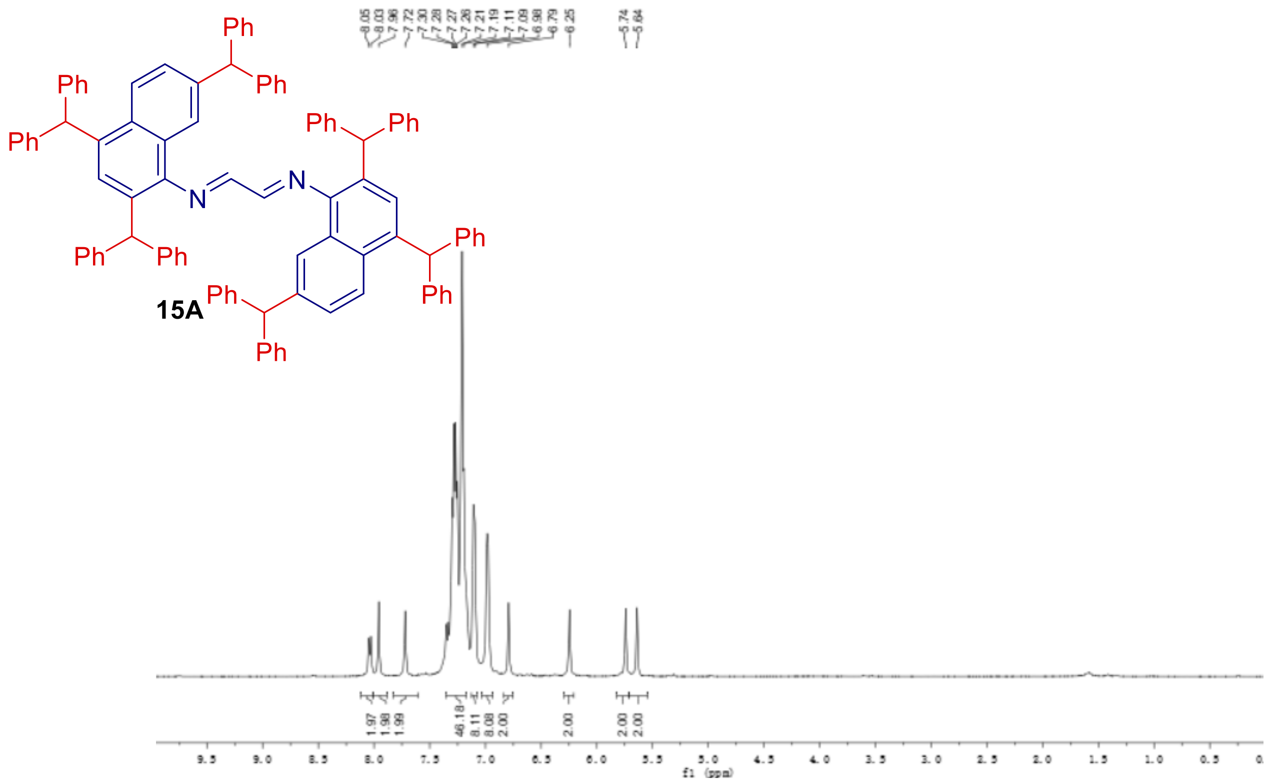

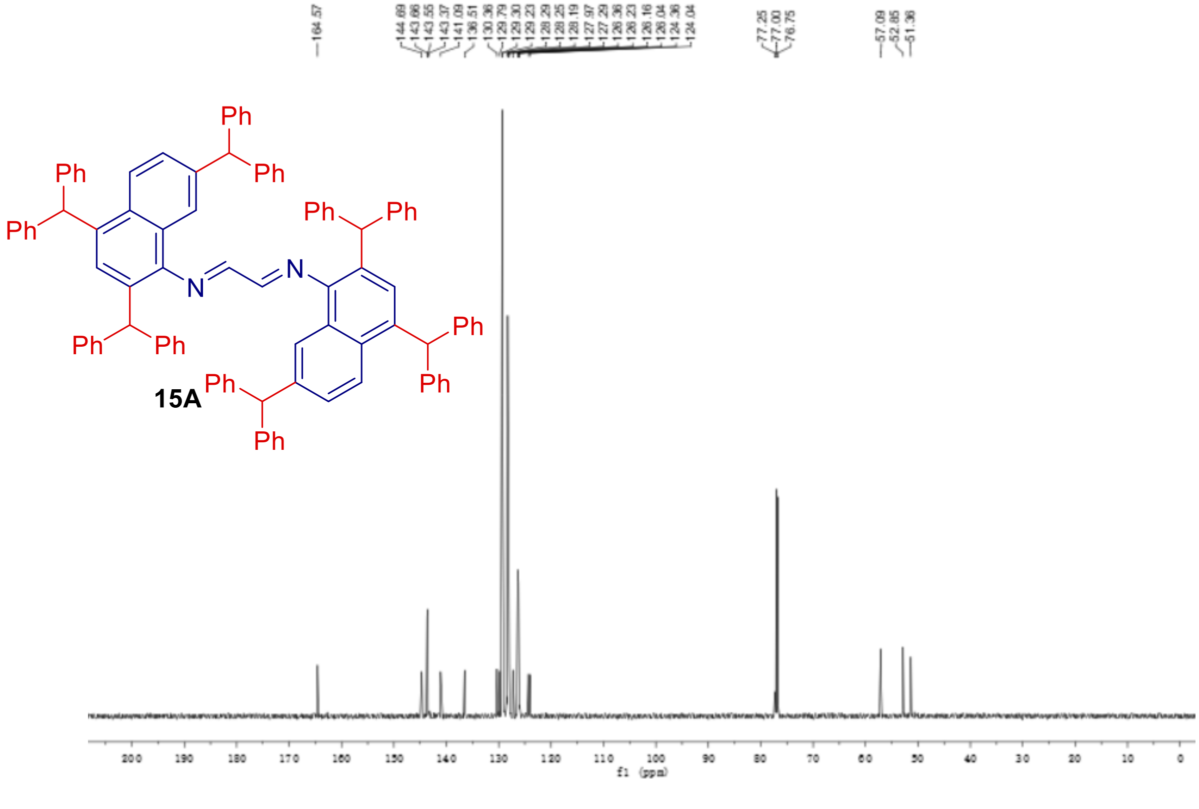

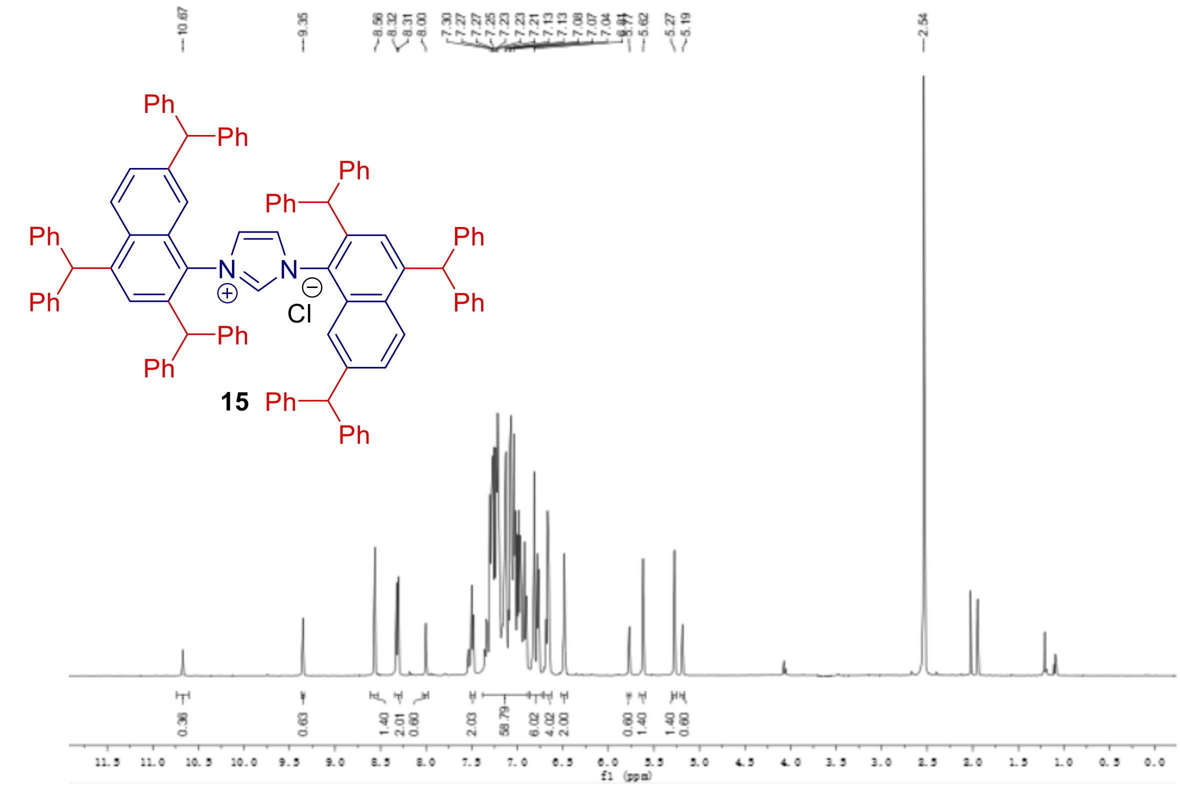

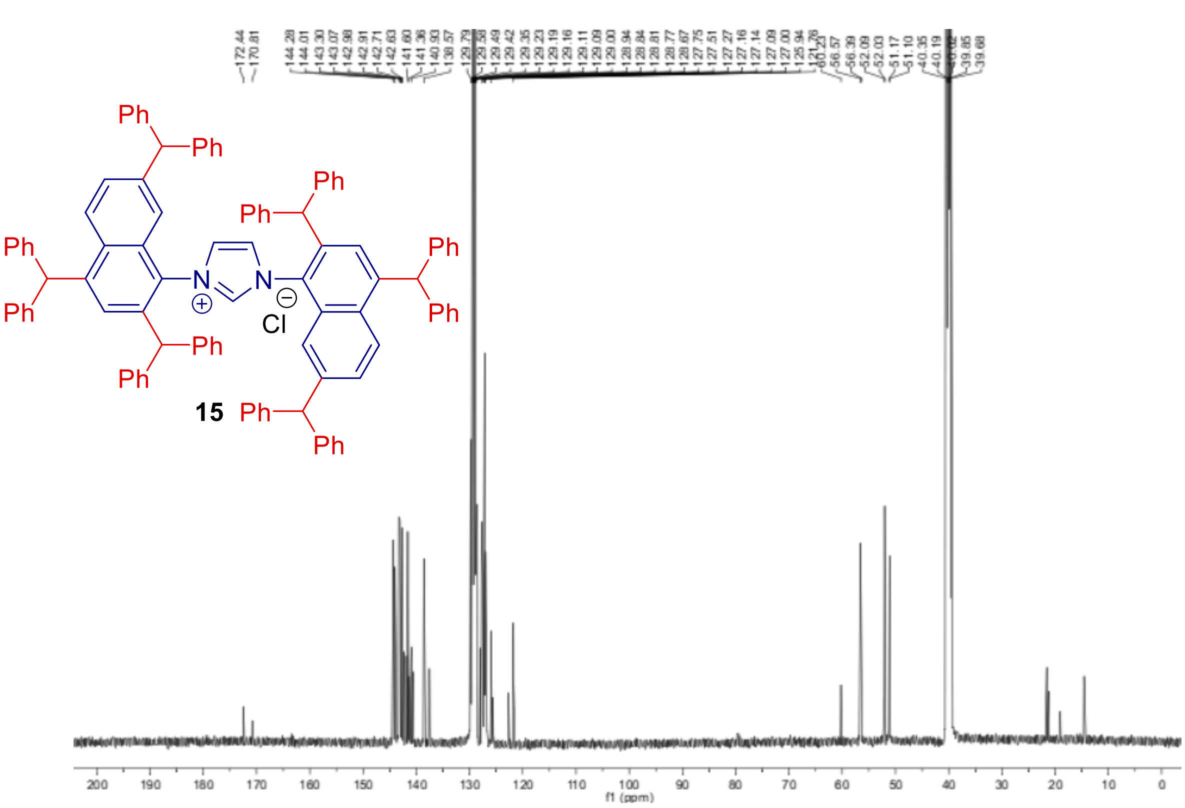

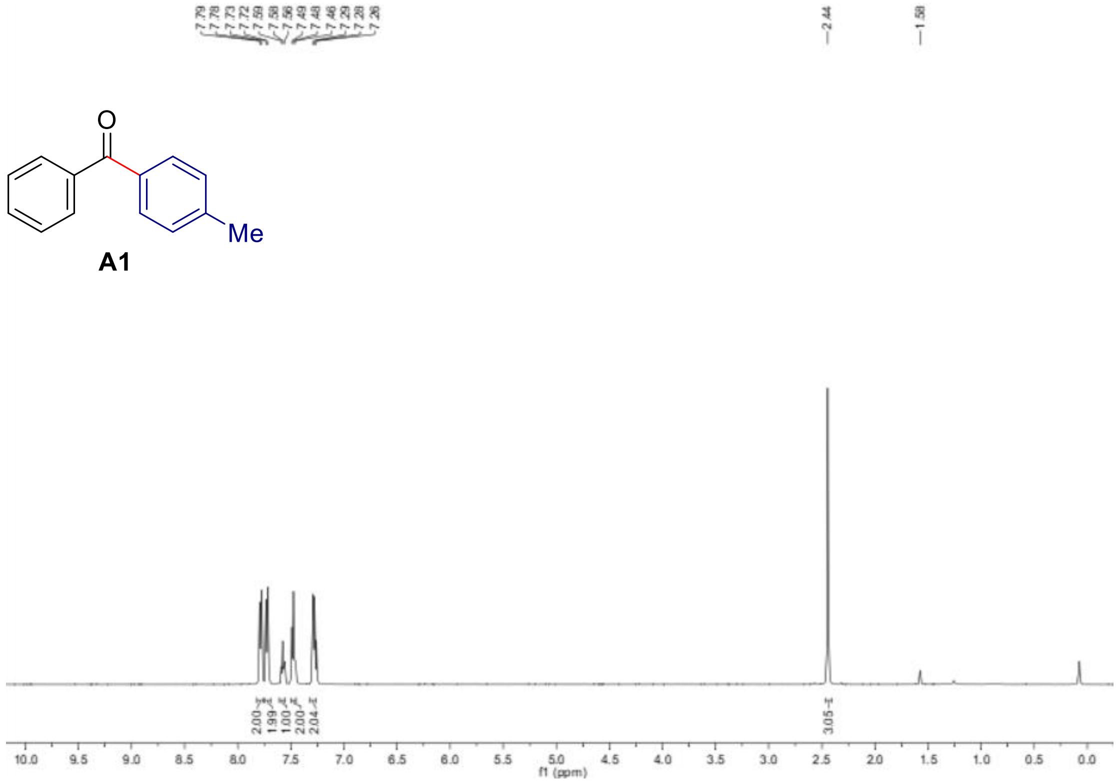
7.79
7.78
7.73
7.72
7.59
7.58
7.56
7.49
7.48
7.46
7.29
7.28
7.26
2.44
1.58
O
Me
A1
2.00
1.99
1.00
2.00
2.04
3.05
f1 (ppm)

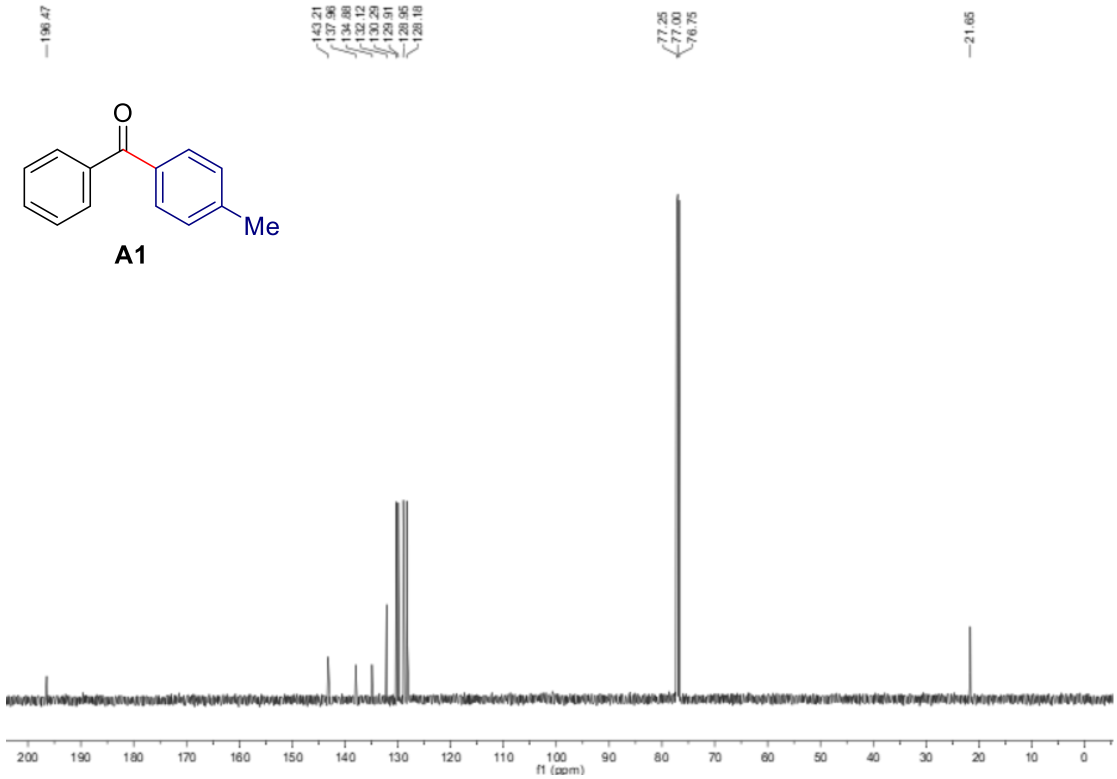
196.47
143.21
137.96
134.88
132.12
130.29
129.91
128.95
128.18
77.25
77.00
76.75
21.65
O
Me
A1
f1 (ppm)

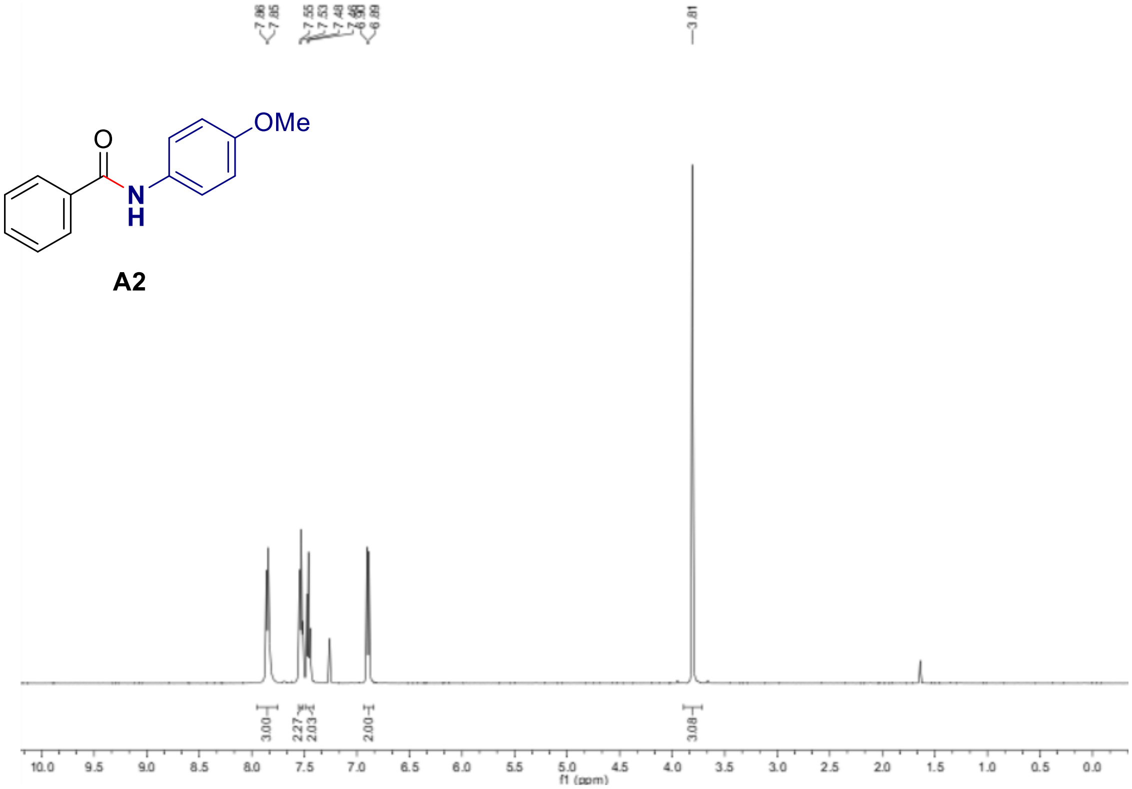
7.86
7.85
7.55
7.53
7.48
6.90
6.89
3.81
OMe
O
N
H
A2
3.00
2.27
2.03
2.00
3.08
10.0
9.5
9.0
8.5
8.0
7.5
7.0
6.5
6.0
5.5
5.0
4.5
4.0
3.5
3.0
2.5
2.0
1.5
1.0
0.5
0.0
f1 (ppm)

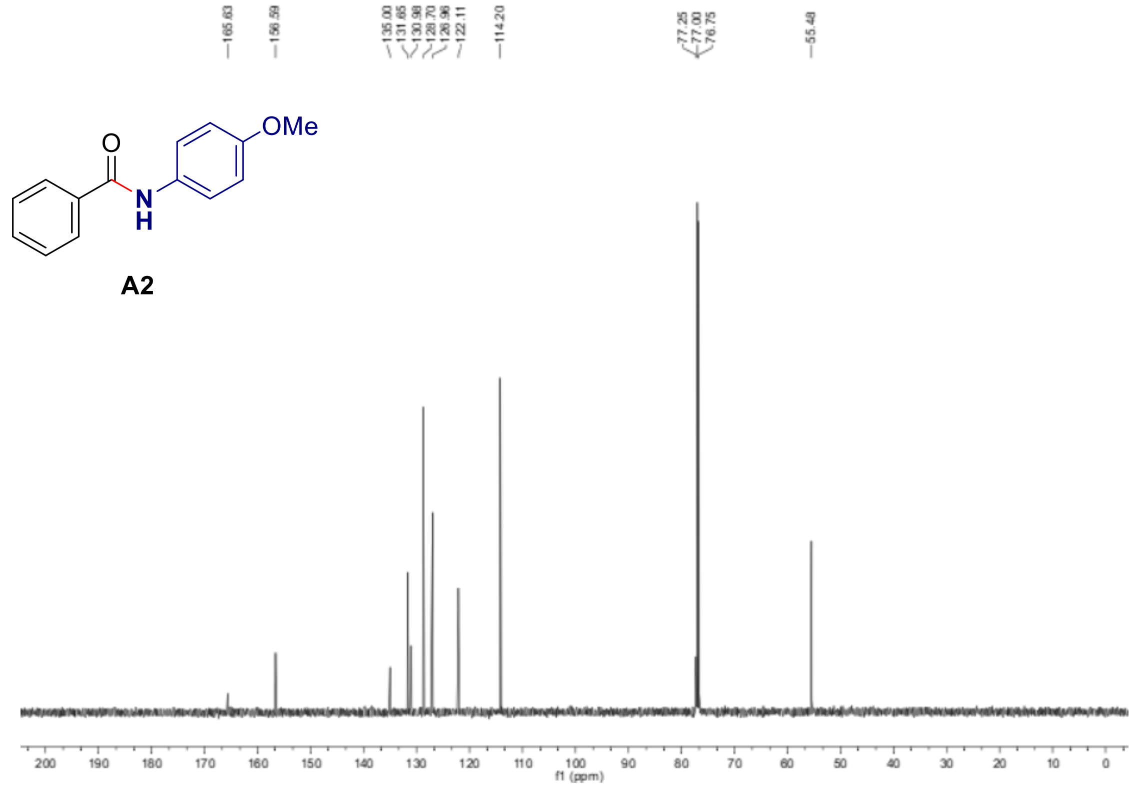
165.63
156.59
135.00
131.65
130.98
128.70
126.96
122.11
114.20
77.25
77.00
76.75
55.48
OMe
O
N
H
A2
200
190
180
170
160
150
140
130
120
110
100
90
80
70
60
50
40
30
20
10
0
f1 (ppm)

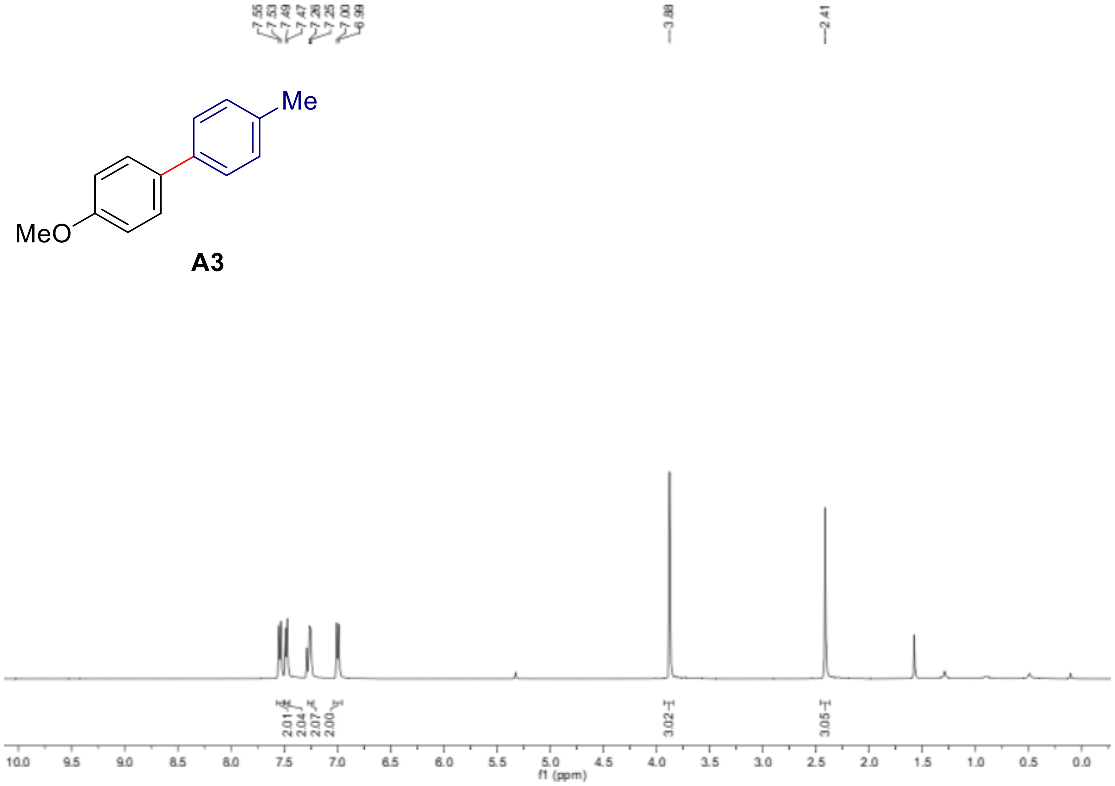
7.55
7.53
7.49
7.47
7.26
7.25
7.00
6.99
3.88
2.41
Me
MeO
A3
2.01
2.04
2.07
2.00
3.02
3.05
10.0 9.5 9.0 8.5 8.0 7.5 7.0 6.5 6.0 5.5 5.0 4.5 4.0 3.5 3.0 2.5 2.0 1.5 1.0 0.5 0.0
f1 (ppm)

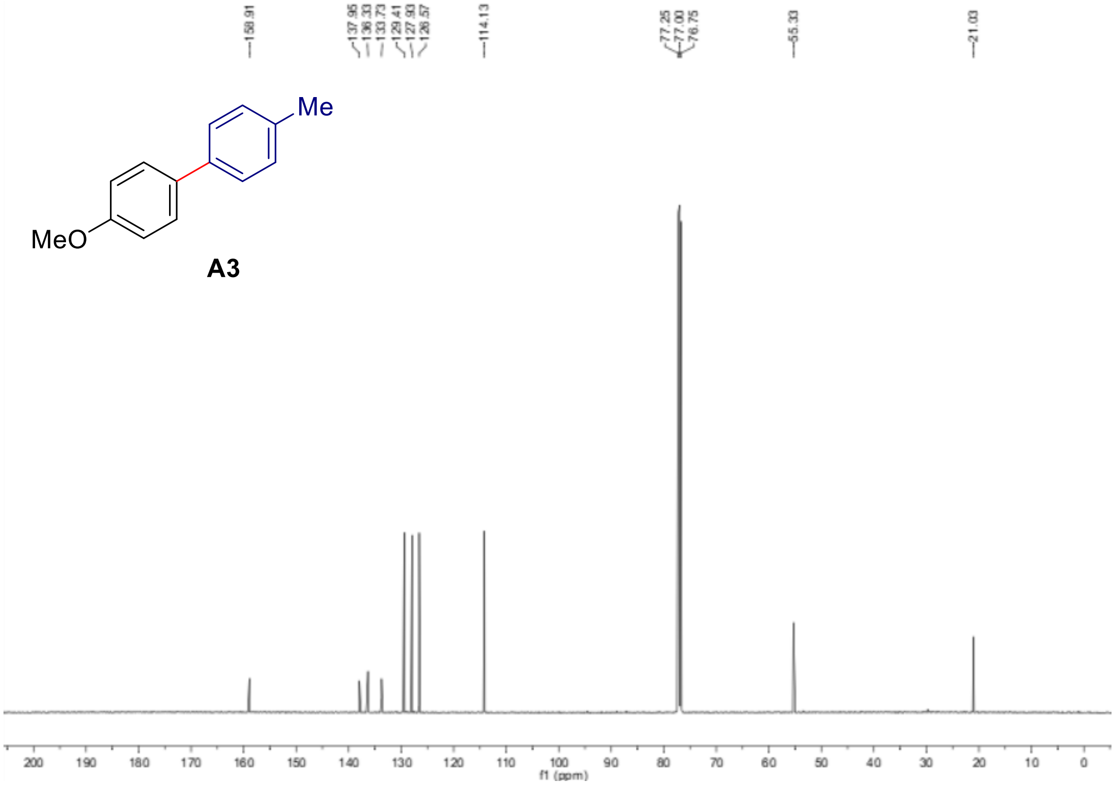
158.91
137.95
136.33
133.73
129.41
127.93
126.57
114.13
77.25
77.00
76.75
55.33
21.03
Me
MeO
A3
200 190 180 170 160 150 140 130 120 110 100 90 80 70 60 50 40 30 20 10 0
f1 (ppm)

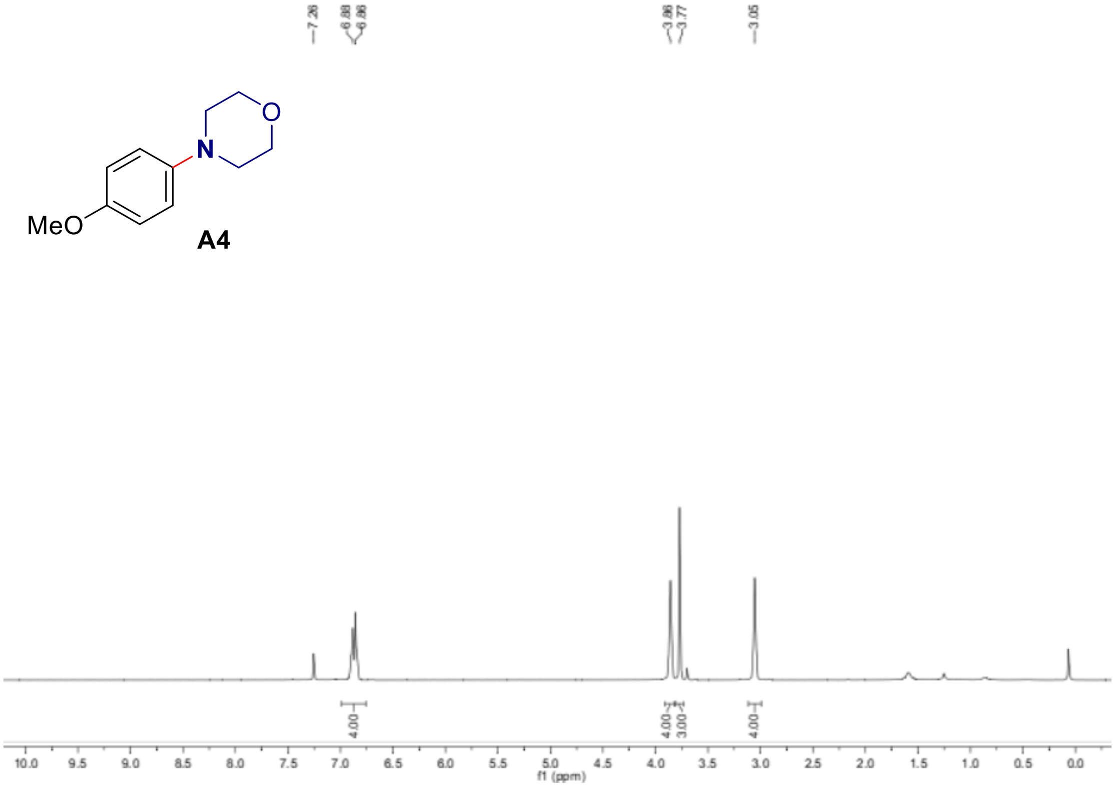
7.26
6.88
6.86
3.86
3.77
3.05
MeO
N
O
A4
4.00
4.00
3.00
4.00
f1 (ppm)

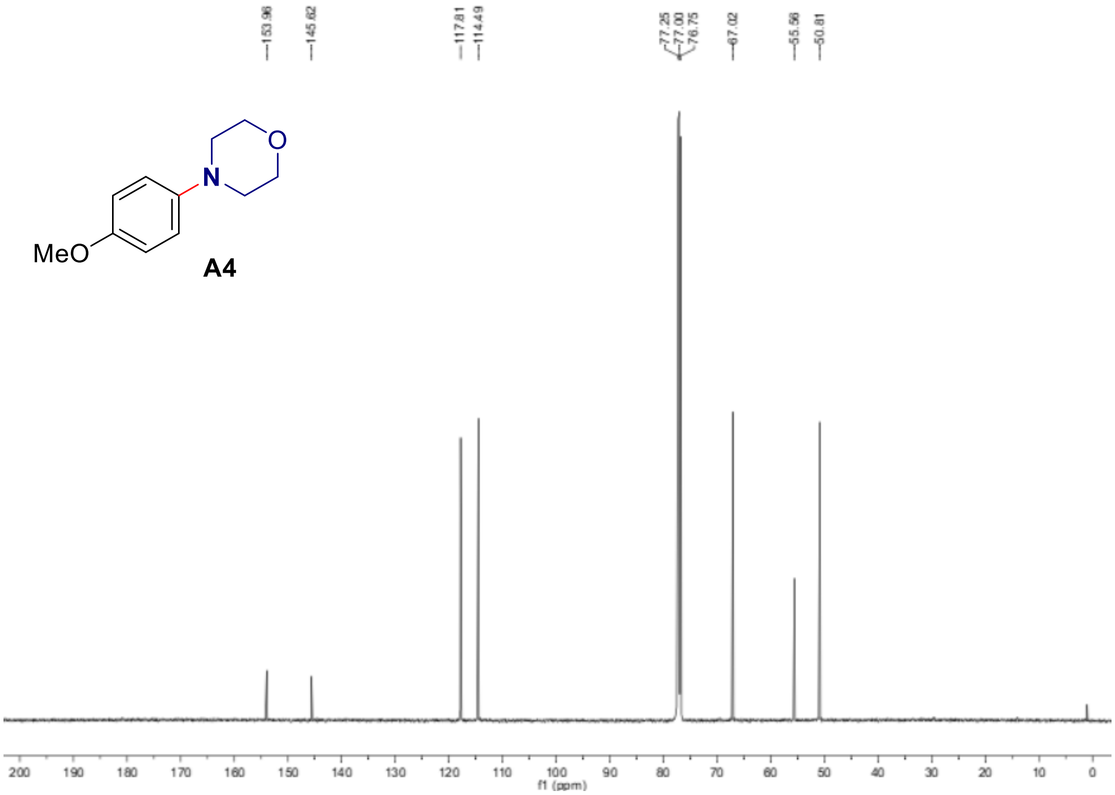
153.96
145.62
117.81
114.49
77.25
77.00
76.75
67.02
55.56
50.81
MeO
N
O
A4
f1 (ppm)

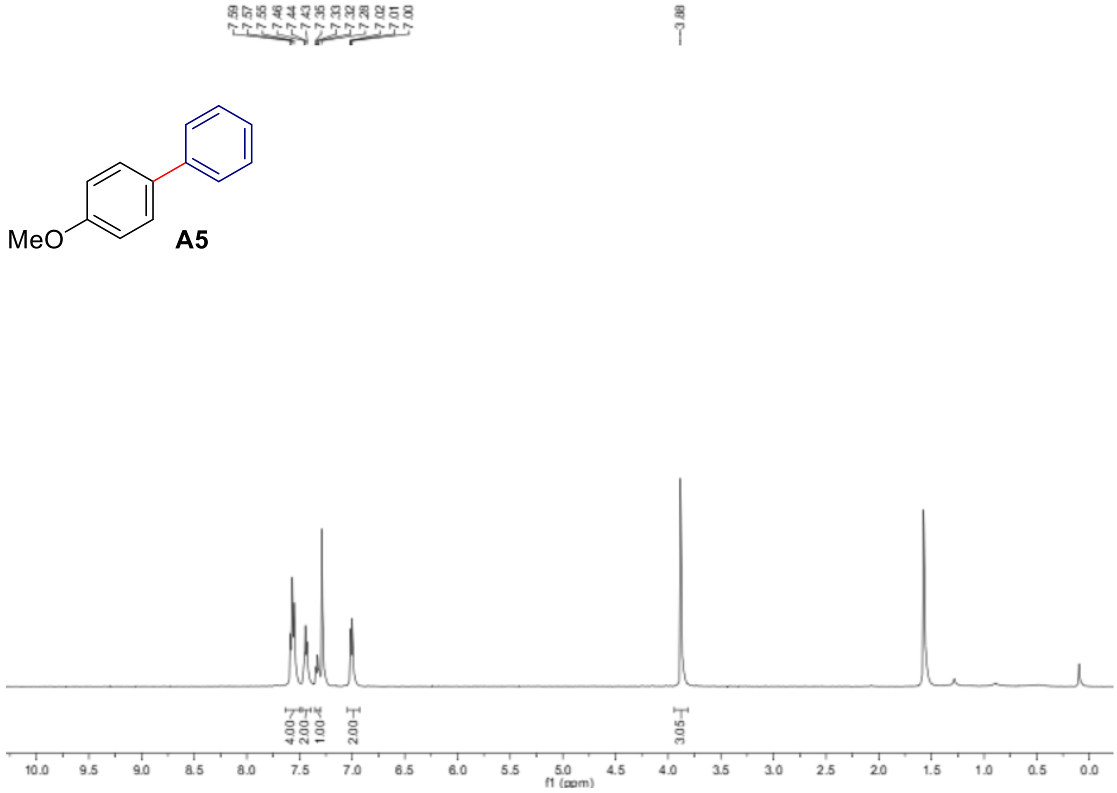
MeO
A5
f1 (ppm)

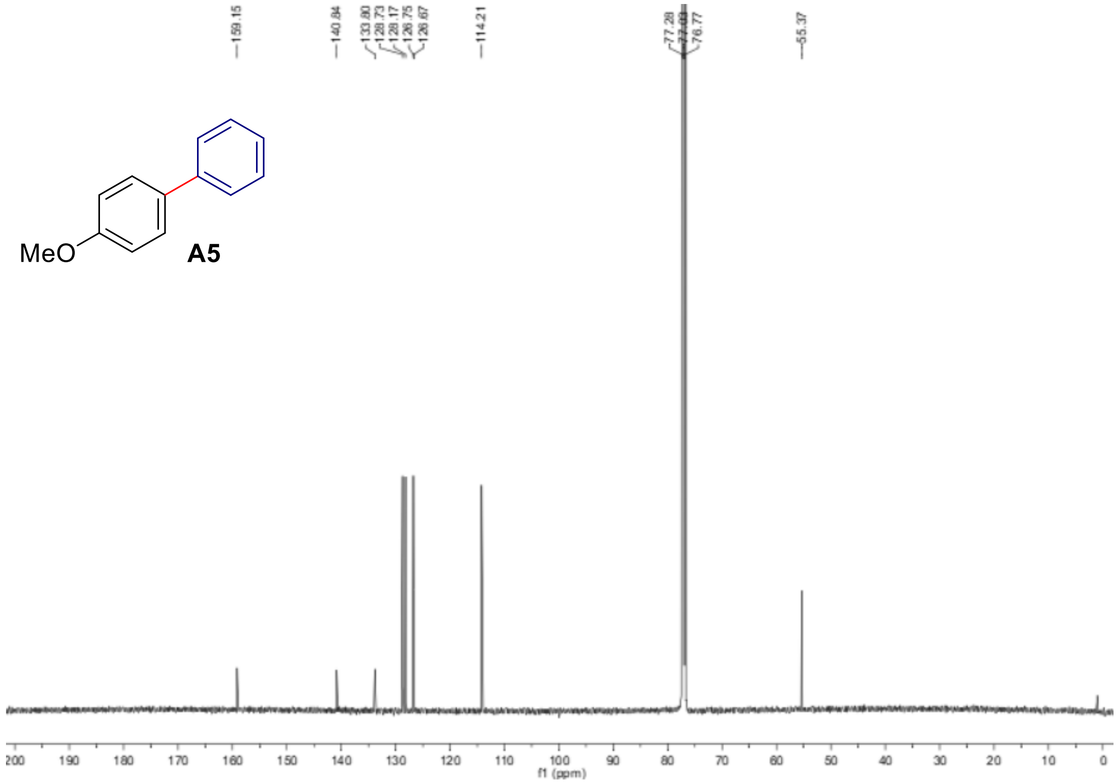
MeO
A5
f1 (ppm)

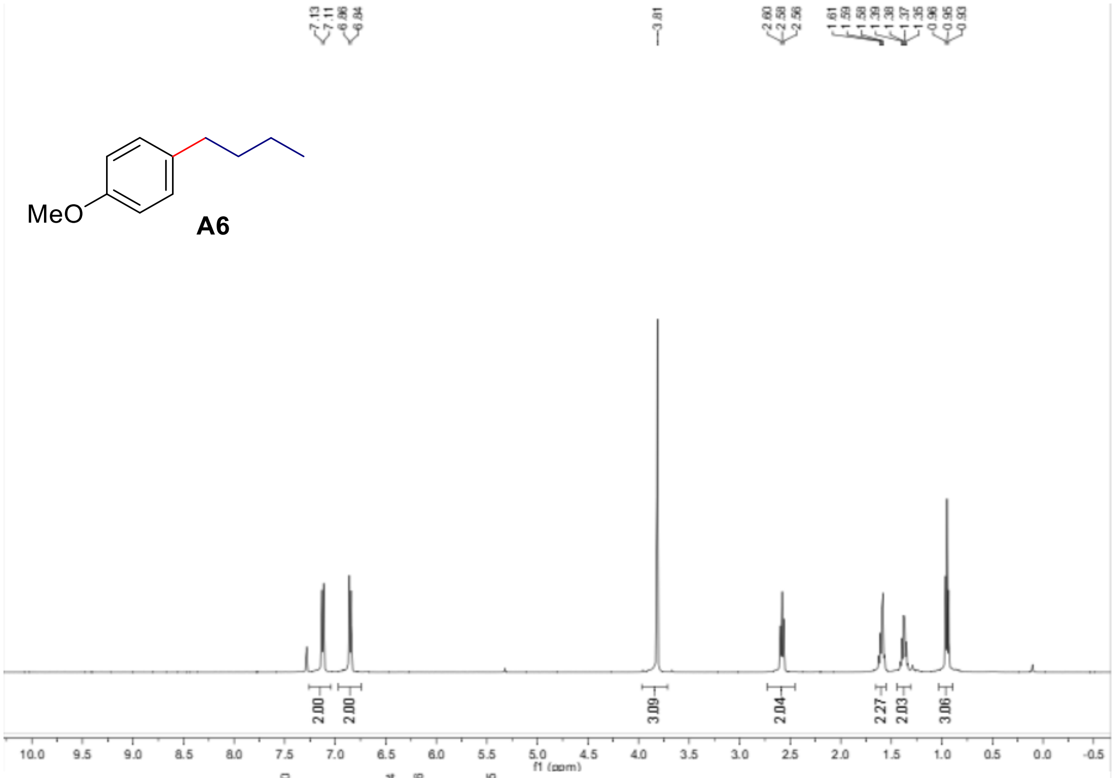
7.13
7.11
6.86
6.84
3.81
2.60
2.58
2.56
1.61
1.59
1.58
1.39
1.38
1.37
1.35
0.96
0.95
0.93
MeO
A6
2.00
2.00
3.09
2.04
2.27
2.03
3.06
f1 (ppm)

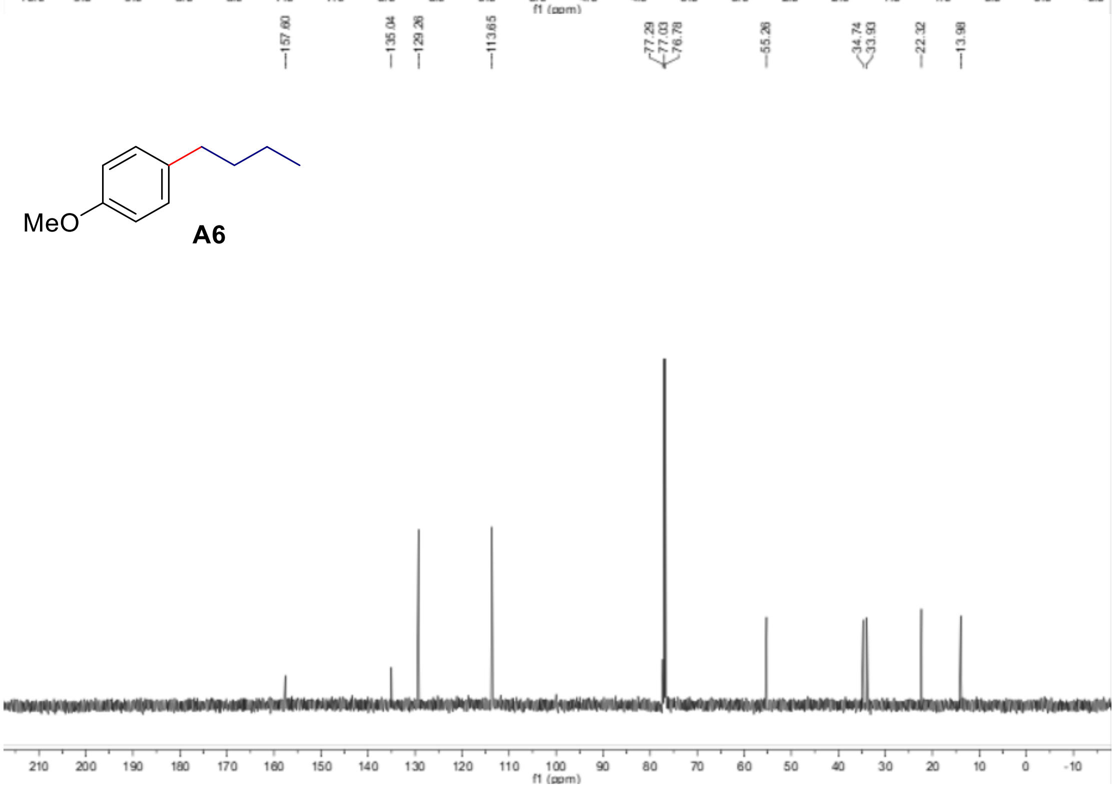
157.60
135.04
129.26
113.65
77.29
77.03
76.78
55.26
34.74
33.93
22.32
13.98
MeO
A6
f1 (ppm)

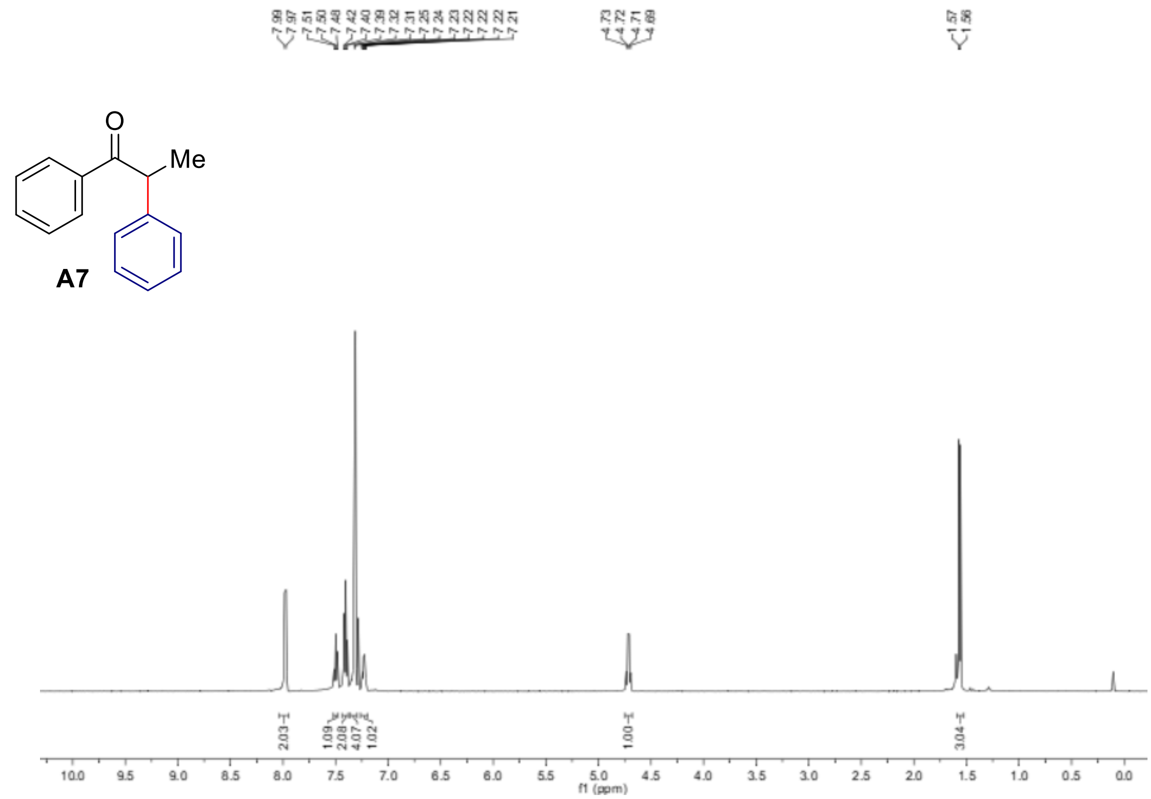
7.99
7.97
7.51
7.50
7.48
7.42
7.40
7.39
7.32
7.31
7.25
7.24
7.23
7.22
7.22
7.22
7.21
4.73
4.72
4.71
4.69
1.57
1.56
O
Me
A7
2.03
1.09
2.08
4.07
1.02
1.00
3.04
f1 (ppm)

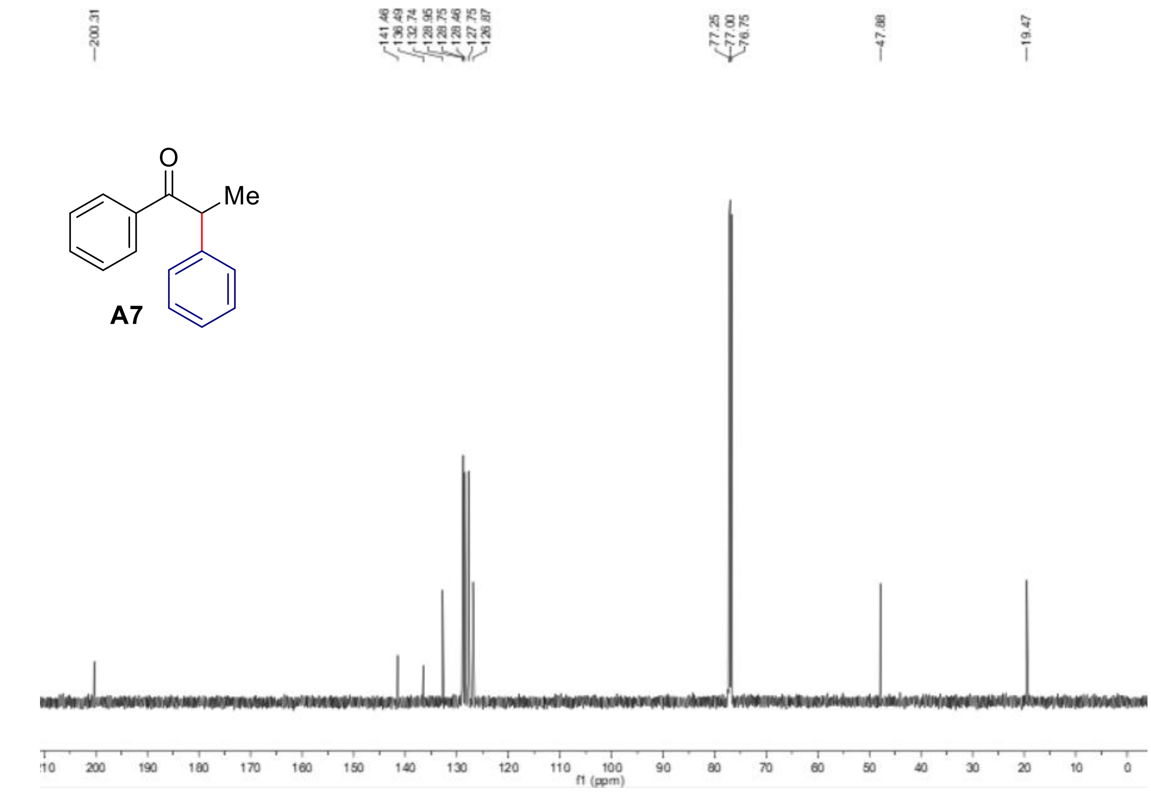
200.31
141.46
136.49
132.74
128.95
128.75
128.46
127.75
126.87
77.25
77.00
76.75
47.88
19.47
O
Me
A7
f1 (ppm)

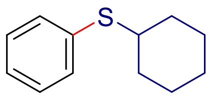
S

A8

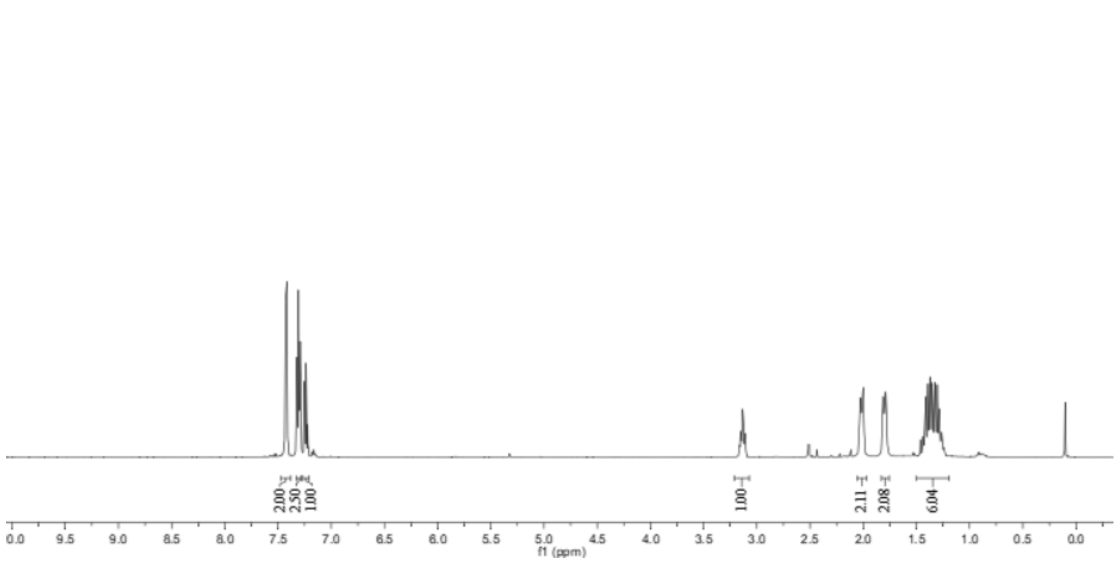
7.43
7.43
7.42
7.41
7.32
7.31
7.31
7.30
7.29
7.28
7.25
7.24
3.15
3.14
3.13
3.13
3.11
2.03
2.00
2.00
1.81
1.81
1.79
1.39
1.37
1.35
1.33
1.30
2.00
2.50
1.00
1.00
2.11
2.08
6.04
f1 (ppm)

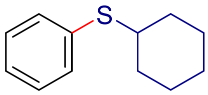
S

A8

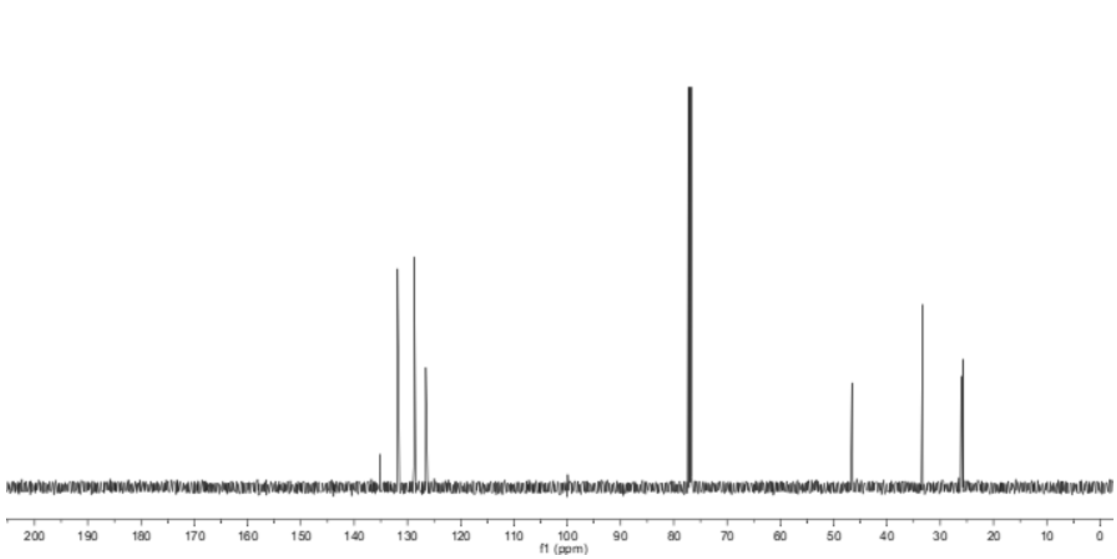
135.17
131.85
128.72
126.56
77.25
77.00
76.75
46.57
33.34
26.05
25.76
f1 (ppm)

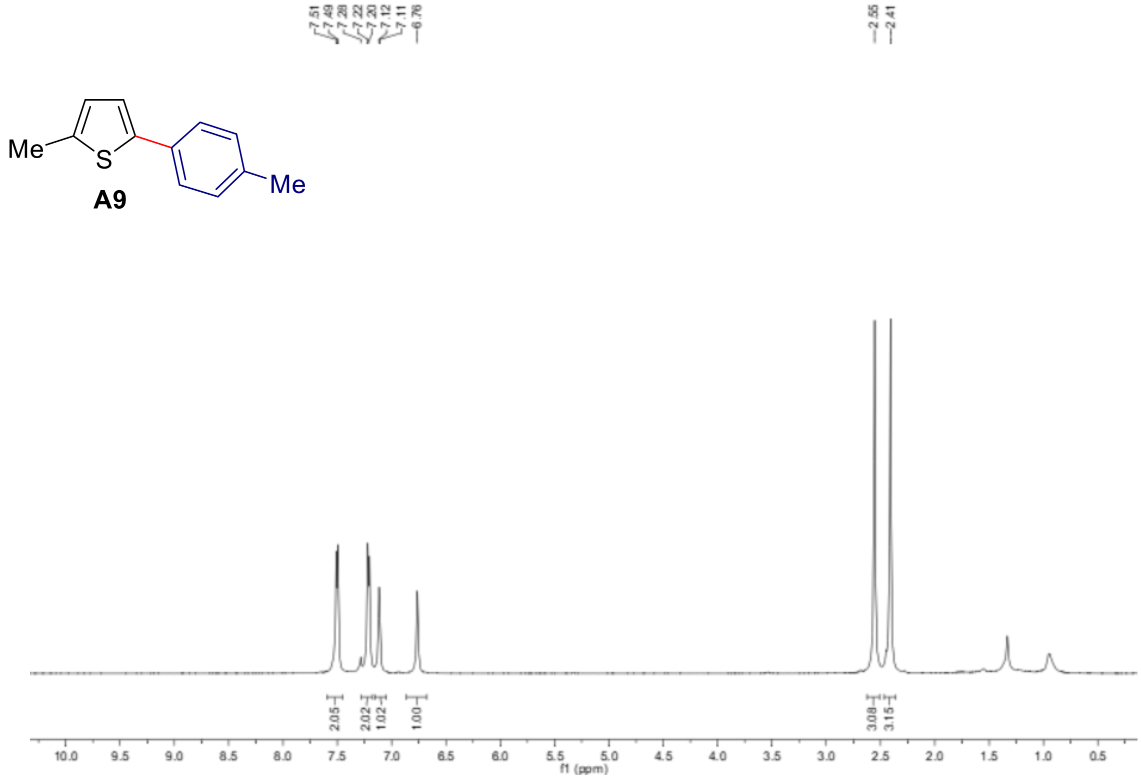
7.51
7.49
7.28
7.22
7.20
7.12
7.11
6.76
2.55
2.41
Me
S
Me
A9
2.05
2.02
1.02
1.00
3.08
3.15
f1 (ppm)

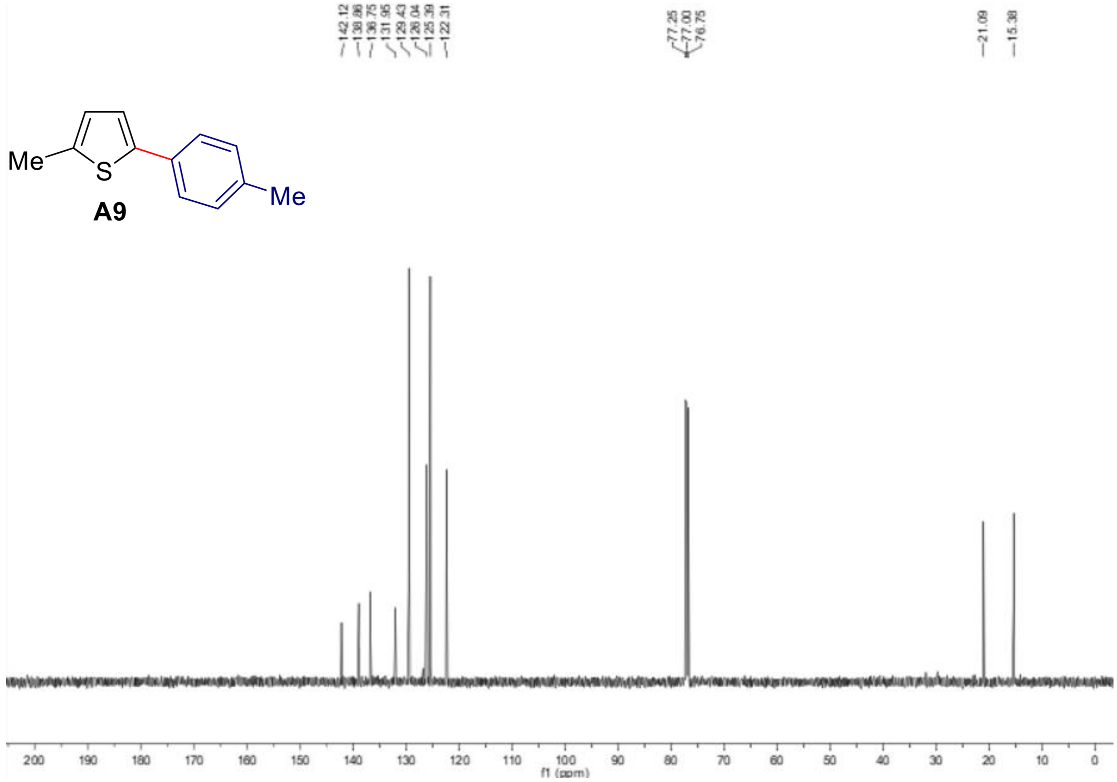
142.12
138.86
136.75
131.95
129.43
126.04
125.39
122.31
77.25
77.00
76.75
21.09
15.38
Me
S
Me
A9
f1 (ppm)

IPr#

Energy: -3697.632984 au

Sum of electronic and thermal Energies: -3696.188550 au

Geometry:

N	-1.04962500	0.01604000	1.05318600
C	-5.02728100	-0.63386300	-0.27435000
C	-4.25034000	-1.68729600	0.20630200
H	-4.65943800	-2.69162700	0.20190100
C	-6.43560500	-0.92427700	-0.79755200
H	-6.77624600	-1.81380400	-0.25734800
C	-3.90879500	2.74052300	2.35017300
H	-4.60357600	1.94947900	2.09505900
C	-3.17123200	0.88888600	0.15893200
C	-4.08907900	3.45046900	3.53901600
H	-4.92202600	3.20357000	4.18844000
C	-7.78867600	1.19163800	-1.34873300
H	-7.37783800	1.18599300	-2.35141200
C	-3.06876600	3.11832700	-1.05345600
C	-0.63917700	0.07046100	2.38611100
H	-1.32617100	0.00542600	3.21178800
C	-2.94769200	-1.49523200	0.66917200
C	-6.46163000	-1.30457500	-2.28109900
C	-7.43285000	0.18450600	-0.44761000
C	-4.47397800	0.64527900	-0.28368300
H	-5.06270900	1.47800700	-0.64760600
C	-2.84540800	3.03757300	1.49334400

C	-2.60789500	2.31156000	0.16502700
H	-1.52589800	2.21655200	0.05832000
C	-4.09234300	4.06732800	-0.99494200
H	-4.57863900	4.27776800	-0.04955700
C	-2.14435100	-2.67374000	1.22535500
H	-1.08989100	-2.42663400	1.08417400
C	-2.41045700	-0.19587400	0.62637000
C	-2.37831200	-3.96019600	0.42314400
C	-2.33002100	-2.86708000	2.73504900
C	-5.52119200	-0.83776800	-3.20299400
H	-4.70955800	-0.20105700	-2.87227700
C	-3.49623900	-2.50117400	3.41295300
H	-4.30988400	-2.03313400	2.87212700
C	-3.20685700	4.46764600	3.89205900
H	-3.34515000	5.01642600	4.81699200
C	-1.96563300	4.06444200	1.85974700
H	-1.13907200	4.31218800	1.20225800
C	-8.67838200	2.19989500	-0.97641800
H	-8.94074200	2.97244600	-1.69090100
C	-1.29168600	-3.45338100	3.47071200
H	-0.37385300	-3.73733800	2.96689000
C	-7.48597500	-2.14158100	-2.74179300
H	-8.22491100	-2.51513200	-2.03956600
C	-8.89205600	1.20878800	1.20627700
H	-9.32428400	1.20285200	2.20105400
C	-9.23318900	2.21331400	0.30081300

H	-9.92903800	2.99394100	0.58719700
C	-2.14097200	4.77264600	3.04482900
H	-1.44489400	5.56137700	3.30948000
C	-3.14260000	-5.02711600	0.90182700
H	-3.58292500	-4.97374500	1.89044600
C	-2.44311000	2.88407200	-2.28495600
H	-1.63747900	2.15889400	-2.34011000
C	-2.58684700	-3.30611500	5.50485200
H	-2.68499800	-3.47243500	6.57181800
C	-1.99186500	-5.21040500	-1.62525900
H	-1.53350500	-5.27467200	-2.60585200
C	-1.80437800	-4.06973600	-0.85057500
H	-1.20858000	-3.25067900	-1.24053100
C	-3.62491800	-2.71981400	4.78616000
H	-4.53838200	-2.42620000	5.29203100
C	-4.49076300	4.75724400	-2.14114400
H	-5.28744700	5.49035100	-2.07518400
C	-1.41613100	-3.67175000	4.83959100
H	-0.59604700	-4.12268900	5.38755400
C	-8.00033600	0.20686700	0.83265000
H	-7.74128000	-0.57188700	1.54370200
C	-7.57315600	-2.49840400	-4.08420900
H	-8.37250500	-3.15129900	-4.41739500
C	-0.00262600	0.11229300	0.16990500
C	-2.76279100	-6.26737400	-1.13996700
H	-2.91025300	-7.15735500	-1.74148200

C	-3.33642400	-6.17085400	0.12459600
H	-3.93354900	-6.98809600	0.51457300
C	-5.60770700	-1.19081400	-4.55057100
H	-4.86703200	-0.81760200	-5.24934200
C	-6.63235600	-2.02095800	-4.99674000
H	-6.69560800	-2.29886400	-6.04287900
C	-3.86702900	4.51192800	-3.36155100
H	-4.17406000	5.05101400	-4.25100600
C	-2.83751200	3.57245800	-3.42855100
H	-2.33645100	3.38120500	-4.37137200
N	1.07290500	0.23022500	1.01175100
C	5.12393000	0.44478800	-0.24445000
C	4.45693200	1.57675700	0.22025800
H	4.99291100	2.51740700	0.26473600
C	6.57902500	0.57151100	-0.70080100
H	7.00594500	1.38773600	-0.10885200
C	3.71756300	-2.98866700	2.03542600
H	4.31816700	-2.08860500	1.99889200
C	3.08357500	-0.84739800	0.09316000
C	3.96991000	-3.93457700	3.03248700
H	4.76190800	-3.75367700	3.75107900
C	7.71729500	-1.65655000	-1.30336800
H	7.38541800	-1.54484100	-2.32873400
C	2.51706200	-2.76408100	-1.43132600
C	0.70607900	0.19581700	2.36112000
H	1.42463200	0.24371600	3.16102500

C	3.12004000	1.54455400	0.61876200
C	6.70849200	1.00556300	-2.16326700
C	7.41307600	-0.66848200	-0.36338900
C	4.41940000	-0.75445600	-0.30527200
H	4.91659900	-1.64618100	-0.66699200
C	2.70799100	-3.19314400	1.09230500
C	2.36227900	-2.19211000	-0.01548000
H	1.29764700	-1.98125300	0.09159500
C	3.42619300	-3.77695300	-1.74742400
H	4.03418800	-4.21952300	-0.96676700
C	2.43356100	2.84406400	1.05569400
H	1.49055600	2.56517600	1.53042700
C	2.44007700	0.31274300	0.56342000
C	2.05235900	3.75850300	-0.12000500
C	3.24997900	3.56806700	2.13339000
C	5.79474300	0.62468000	-3.15037800
H	4.93419500	0.02098100	-2.88731700
C	4.22450300	4.52288500	1.82065900
H	4.38240900	4.81089100	0.78757400
C	3.21626700	-5.10137700	3.10376600
H	3.41467200	-5.83657000	3.87573600
C	1.95119400	-4.37213500	1.17950600
H	1.16337400	-4.55549000	0.45604800
C	8.45717700	-2.78283300	-0.94011100
H	8.68277700	-3.53799900	-1.68516200
C	3.03884100	3.25800300	3.48145800

H	2.27158200	2.53913500	3.74905700
C	7.79981700	1.79876300	-2.53836500
H	8.51948700	2.10483700	-1.78533000
C	8.62104900	-1.95134900	1.31219800
H	8.97650700	-2.05403900	2.33173900
C	8.91115800	-2.93551000	0.36721200
H	9.49023700	-3.80834900	0.64712900
C	2.20121700	-5.31651200	2.16964400
H	1.60343000	-6.22051100	2.21227400
C	1.44432500	4.99237200	0.15713500
H	1.29654900	5.29373400	1.18871600
C	1.72874900	-2.22462400	-2.45696100
H	1.02198700	-1.43496000	-2.21918100
C	4.76525200	4.80383200	4.16085600
H	5.34767600	5.28153400	4.94066000
C	1.84139500	4.25890700	-2.48777000
H	2.00074200	3.96081300	-3.51823000
C	2.23975300	3.40395800	-1.45760800
H	2.69579600	2.45509700	-1.70795500
C	4.97816000	5.13140000	2.82301800
H	5.72700400	5.86966000	2.55728500
C	3.55013800	-4.23803600	-3.05892000
H	4.26047100	-5.02595000	-3.28509000
C	3.78711200	3.86658200	4.48736700
H	3.60030900	3.61343900	5.52529700
C	7.87913600	-0.83131700	0.94762100

H	7.65741300	-0.07046800	1.68989900
C	7.97782000	2.19749200	-3.86044600
H	8.82829300	2.81545700	-4.12693900
C	1.24332400	5.48062000	-2.19899700
H	0.93097400	6.14199800	-2.99914400
C	1.04372700	5.84433300	-0.86684000
H	0.57450300	6.79194700	-0.62583800
C	5.97225800	1.01897800	-4.47706500
H	5.25208900	0.71102700	-5.22718700
C	7.06292600	1.80634000	-4.83753800
H	7.19719400	2.11681000	-5.86766300
C	2.76282800	-3.69620000	-4.07195500
H	2.85605400	-4.05878900	-5.08971900
C	1.84907900	-2.68719600	-3.76499400
H	1.22636200	-2.26192200	-4.54488400

BIAN-IPr#

Energy: -4081.193258 au

Sum of electronic and thermal Energies: -4079.637057 au

Geometry:

N	-1.07836000	0.05787000	0.69226900
C	-5.01667500	-0.76389100	-0.67055400
C	-4.19724700	-1.78294400	-0.18784300
H	-4.55457400	-2.80667500	-0.21654600
C	-6.40290900	-1.11462900	-1.21589900
H	-6.71624600	-2.01477300	-0.67671000

C	-4.29819200	2.87813200	1.73289100
H	-5.02480600	2.15886200	1.37443200
C	-3.23497400	0.83749400	-0.20870500
C	-4.58758000	3.62540000	2.87487700
H	-5.53493800	3.48050500	3.38293000
C	-7.83308000	0.94065100	-1.80127100
H	-7.40752500	0.94546400	-2.79778500
C	-3.16486400	3.00920400	-1.50966300
C	-0.64705100	0.24948700	1.99904500
C	-2.91257800	-1.53571200	0.29958500
C	-6.38756400	-1.50209100	-2.69755100
C	-7.44992600	-0.04557600	-0.88823700
C	-4.52070000	0.53766500	-0.66757400
H	-5.13623800	1.34319500	-1.04842100
C	-3.08667100	3.04616100	1.05406500
C	-2.73577500	2.28431200	-0.22677100
H	-1.64620300	2.23914100	-0.26818700
C	-4.12967800	4.01833400	-1.53482200
H	-4.59832100	4.34100100	-0.61300000
C	-2.07203300	-2.69171700	0.84734700
H	-1.03117600	-2.36550700	0.81692600
C	-2.43243900	-0.21253000	0.26951400
C	-2.14476800	-3.92599400	-0.06474300
C	-2.36765400	-3.01215200	2.31651300
C	-5.45946600	-0.98932800	-3.60753900
H	-4.68733200	-0.30966100	-3.26772100

C	-3.64988900	-2.94550600	2.87046200
H	-4.48342500	-2.61059500	2.26535600
C	-3.66775000	4.54996600	3.36388500
H	-3.89093800	5.12607100	4.25490700
C	-2.17149600	3.97753000	1.55805200
H	-1.22697500	4.12046700	1.04461900
C	-8.76821900	1.91437800	-1.44879200
H	-9.05095400	2.67121100	-2.17224200
C	-1.31396800	-3.42991700	3.13813000
H	-0.30910000	-3.48406100	2.73323200
C	-7.35970900	-2.39325200	-3.16933700
H	-8.08808900	-2.80291900	-2.47626600
C	-8.97383000	0.92909300	0.73730200
H	-9.42068700	0.91134700	1.72548600
C	-9.34195900	1.91331600	-0.17989000
H	-10.07318000	2.66672500	0.09082400
C	-2.45406100	4.72234100	2.70032600
H	-1.72526800	5.43338400	3.07380400
C	-2.77817600	-5.11473600	0.30496900
H	-3.23799300	-5.20058100	1.28197800
C	-2.56243200	2.62984200	-2.71716200
H	-1.79834700	1.85910100	-2.70942600
C	-2.81753200	-3.71583200	5.00642800
H	-2.99085800	-3.98362800	6.04274100
C	-1.57152600	-4.94302300	-2.19669900
H	-1.08862800	-4.86891900	-3.16464400

C	-1.54109400	-3.85703700	-1.32784800
H	-1.04454300	-2.94144200	-1.63174700
C	-3.87426600	-3.29704600	4.20182800
H	-4.87790500	-3.23881800	4.60955800
C	-4.49549300	4.62523500	-2.73855800
H	-5.24767500	5.40679000	-2.73648200
C	-1.53314000	-3.77791900	4.46851700
H	-0.69793600	-4.09136100	5.08496400
C	-8.03666300	-0.03807400	0.38358700
H	-7.75669800	-0.80097100	1.10381800
C	-7.40831000	-2.75870400	-4.51145400
H	-8.16703000	-3.45411600	-4.85347200
C	-0.02178000	0.06581000	-0.20162900
C	-2.21399200	-6.12339800	-1.82048400
H	-2.23950200	-6.97108100	-2.49613000
C	-2.81613400	-6.20403700	-0.56854000
H	-3.31391500	-7.11781200	-0.26216900
C	-5.50779300	-1.35074300	-4.95470700
H	-4.77769900	-0.94139400	-5.64429100
C	-6.48067400	-2.23536300	-5.41211000
H	-6.51376100	-2.51985000	-6.45786900
C	-3.89889900	4.23355400	-3.93326100
H	-4.18276100	4.70541400	-4.86753300
C	-2.92579100	3.23292500	-3.91674100
H	-2.44357800	2.92804700	-4.83920300
N	1.07887800	0.26905900	0.60680300

C	5.13872300	0.44858100	-0.62934000
C	4.43024000	1.61002500	-0.32082000
H	4.94095800	2.56374900	-0.37706300
C	6.60575600	0.56103300	-1.04985800
H	6.99278400	1.44986100	-0.54097300
C	3.79678400	-2.89927200	1.83609800
H	4.40354100	-2.00766900	1.74286100
C	3.11619400	-0.84748500	-0.20920600
C	4.05820100	-3.79288100	2.87769600
H	4.86232500	-3.58039600	3.57372100
C	7.80029700	-1.69801800	-1.35279000
H	7.49248800	-1.71741300	-2.39150900
C	2.60616900	-2.84931300	-1.62274800
C	0.71855000	0.36314800	1.94626500
C	3.08391600	1.58941000	0.04243900
C	6.77821500	0.83030000	-2.54718100
C	7.45449200	-0.61059700	-0.54625200
C	4.46103300	-0.76498200	-0.58375900
H	4.98340500	-1.67784100	-0.84302800
C	2.77019900	-3.14468500	0.92184300
C	2.41933800	-2.20887800	-0.23856700
H	1.34956500	-2.01154100	-0.15666900
C	3.50039300	-3.89518800	-1.86341800
H	4.07170300	-4.31625800	-1.04431600
C	2.33442500	2.91148800	0.24778200
H	1.45597600	2.69339900	0.85912000

C	2.43940100	0.33770200	0.13017900
C	1.80373600	3.50579700	-1.06797000
C	3.15721500	3.93749000	1.03795400
C	5.90952200	0.31699100	-3.51478700
H	5.05332100	-0.27545000	-3.21454600
C	4.00351000	4.85739900	0.40724100
H	4.05961000	4.88102300	-0.67512800
C	3.29634800	-4.94732500	3.02313600
H	3.50077000	-5.64133600	3.83082400
C	2.00518900	-4.31030300	1.08373900
H	1.20414000	-4.52415000	0.38340700
C	8.55162800	-2.75621000	-0.83958000
H	8.80939200	-3.59075000	-1.48248500
C	3.07076100	3.96771200	2.43406500
H	2.40956500	3.27635100	2.94415900
C	7.86477300	1.60421900	-2.97303000
H	8.54951200	2.01211000	-2.23586700
C	8.64490600	-1.65596000	1.29860400
H	8.97787500	-1.62698800	2.33045300
C	8.97615800	-2.74046200	0.48639000
H	9.56448100	-3.56051500	0.88248800
C	2.26399300	-5.20268900	2.11882700
H	1.65851700	-6.09671600	2.22035800
C	0.97317600	4.63390900	-0.99926800
H	0.73231300	5.05968300	-0.03064500
C	1.86709400	-2.33709600	-2.69796700

H	1.17117100	-1.52260800	-2.51991200
C	4.66999600	5.77570900	2.54237100
H	5.25043800	6.48582700	3.12078000
C	1.62022900	3.59597100	-3.48737600
H	1.88057800	3.18163000	-4.45531700
C	2.11491000	2.99302200	-2.32825500
H	2.74611300	2.11860300	-2.41812400
C	4.75739900	5.76342100	1.15135600
H	5.40575600	6.46754000	0.64119300
C	3.65676600	-4.41651300	-3.14893300
H	4.35466200	-5.22992800	-3.31580100
C	3.81892300	4.87619200	3.18127100
H	3.72939700	4.88402800	4.26213600
C	7.89132400	-0.60423700	0.78472100
H	7.63863500	0.23637100	1.42395100
C	8.08185400	1.85590600	-4.32507000
H	8.92766400	2.46177100	-4.63123800
C	0.80062000	4.71609700	-3.40471600
H	0.41337200	5.18106900	-4.30415700
C	0.47373100	5.23248800	-2.15029300
H	-0.17174700	6.09994600	-2.06983300
C	6.12649100	0.56372200	-4.87097400
H	5.44105900	0.15430200	-5.60492400
C	7.21193500	1.33346100	-5.28166100
H	7.37673700	1.52917200	-6.33527800
C	2.91825000	-3.90164300	-4.21118700

H	3.03705300	-4.31035700	-5.20862100
C	2.02008800	-2.85870700	-3.97974900
H	1.43553500	-2.45272100	-4.79856800
C	2.50176200	0.62694100	3.88728400
C	1.26392500	0.51555700	3.29408800
C	0.10521900	0.51934200	4.12969000
C	0.15130900	0.65128900	5.51778600
C	1.44603800	0.77262900	6.10229300
C	2.57186800	0.75426800	5.30346200
H	3.41555000	0.62244100	3.30520900
C	-1.10328400	0.36290100	3.38536200
C	-1.10218500	0.64448600	6.19788000
H	1.54447800	0.87479500	7.17791900
H	3.54949800	0.84144500	5.76443400
C	-2.27458700	0.50591000	5.48428000
C	-2.29681000	0.35968200	4.06847000
H	-1.13064400	0.74735200	7.27752800
H	-3.21969900	0.50268800	6.01542600
H	-3.24491500	0.24783600	3.55822500

Np# (rac)

Energy: -4004.984019 au

Sum of electronic and thermal Energies: -4003.441018 au

Geometry:

N	-1.04124600	0.16919700	0.61252000
N	1.05339400	-0.22420600	0.62084900

C	0.01134500	-0.01512000	-0.24685700
C	-0.66586100	0.07873700	1.95579300
H	-1.36487200	0.19927300	2.76578800
C	0.66270600	-0.17286900	1.96083800
H	1.35066300	-0.32363400	2.77530700
C	-2.38768600	0.43381300	0.17895200
C	-3.31445300	-0.65509400	0.14405700
C	-2.92189400	-1.98226000	0.45819500
H	-1.89081700	-2.16239300	0.73498900
C	-3.80282100	-3.04165800	0.40829700
C	-5.14321800	-2.78373800	0.03663300
H	-5.84990800	-3.60296700	-0.02140500
C	-5.55993000	-1.51100900	-0.27012400
H	-6.59583800	-1.35670800	-0.53990100
C	-4.66939600	-0.40633700	-0.24185200
C	-3.32414700	-4.45194600	0.75203300
H	-2.23922600	-4.44382900	0.60321400
C	-3.54269500	-4.84374800	2.21787800
C	-2.85809900	-5.95998000	2.71982000
H	-2.20025400	-6.52012000	2.06283100
C	-3.01728400	-6.36625800	4.04076000
H	-2.47805300	-7.23358100	4.40619300
C	-3.86955500	-5.66159200	4.89260100
H	-3.99611900	-5.97617700	5.92246300
C	-4.55222700	-4.55059800	4.40751100
H	-5.21524800	-3.99247800	5.05963700

C	-4.38810900	-4.14361800	3.08127500
H	-4.92089500	-3.27048600	2.72498400
C	-3.87113600	-5.49286100	-0.23094900
C	-3.24335500	-5.65088700	-1.47226300
H	-2.36603700	-5.05663200	-1.70765300
C	-3.72793100	-6.56046900	-2.40871500
H	-3.22219200	-6.67150400	-3.36152100
C	-4.85511400	-7.32904800	-2.11984800
H	-5.23308500	-8.03945100	-2.84656300
C	-5.48618500	-7.18277400	-0.88668400
H	-6.35909500	-7.78085300	-0.64848200
C	-4.99526800	-6.27460500	0.05154900
H	-5.48439500	-6.18505600	1.01487100
C	-5.06340900	0.92434700	-0.60499200
C	-6.51290900	1.20363300	-1.02126800
H	-6.79296600	0.39765100	-1.70781000
C	-7.50229600	1.12755000	0.14604400
C	-7.14238100	1.44061800	1.45900000
H	-6.11813300	1.71583500	1.68195500
C	-8.08236200	1.39315000	2.48918000
H	-7.78173300	1.63899300	3.50189200
C	-9.39901900	1.02677300	2.22231600
H	-10.12873800	0.98545000	3.02310900
C	-9.76989600	0.70849600	0.91585000
H	-10.79140100	0.41838100	0.69558900
C	-8.82936700	0.76061400	-0.10917200

H	-9.12905600	0.51762700	-1.12407200
C	-6.68062600	2.49142900	-1.83313100
C	-7.06987900	3.70253100	-1.25372300
H	-7.28038600	3.74831800	-0.19173500
C	-7.20593600	4.85357200	-2.03015900
H	-7.51099900	5.78285000	-1.56141100
C	-6.95989800	4.81063700	-3.40026600
H	-7.07117000	5.70419600	-4.00428100
C	-6.57415200	3.60753400	-3.98988300
H	-6.38065700	3.56048100	-5.05577800
C	-6.43697600	2.46151200	-3.21140600
H	-6.13178800	1.53032000	-3.67858800
C	-4.11620400	1.91869800	-0.58807900
H	-4.39571700	2.91571900	-0.90224400
C	-2.76765300	1.70625200	-0.19364400
C	-1.78356400	2.87425200	-0.22812800
H	-0.78782700	2.44018100	-0.12531900
C	-1.79508300	3.53508700	-1.61310400
C	-1.10039300	2.90698500	-2.65494000
H	-0.56097900	1.98664500	-2.45226700
C	-1.10186900	3.44881800	-3.93743700
H	-0.55469800	2.95139600	-4.73106100
C	-1.80070700	4.62692600	-4.20125000
H	-1.80118200	5.05041600	-5.19969100
C	-2.49482200	5.25722600	-3.17163300
H	-3.04168400	6.17354100	-3.36498600

C	-2.49021200	4.71589500	-1.88599200
H	-3.02262400	5.22603700	-1.09144600
C	-1.92974500	3.86233400	0.93400500
C	-0.98513800	4.89295100	1.05713200
H	-0.20259000	4.99289300	0.31216900
C	-1.04334100	5.79846500	2.11115400
H	-0.30268000	6.58754500	2.18060600
C	-2.05002000	5.69544600	3.07283400
H	-2.09730800	6.40159800	3.89429900
C	-2.99009200	4.67622200	2.96557700
H	-3.77717100	4.58193800	3.70602200
C	-2.92827700	3.76767000	1.90591800
H	-3.66714200	2.97853300	1.84402300
C	2.39954200	-0.46253400	0.17345100
C	2.78868100	-1.72455700	-0.22795900
C	1.85022800	-2.92694000	-0.11684700
H	0.84386600	-2.51961500	-0.01945100
C	2.09340300	-3.74243900	1.15646000
C	3.37002700	-4.07124300	1.62709900
H	4.24842700	-3.73128600	1.09295100
C	3.53441800	-4.82986900	2.78563100
H	4.53432100	-5.07182000	3.12939600
C	2.42423800	-5.27223000	3.50199900
H	2.55338000	-5.85714100	4.40579100
C	1.14669700	-4.94954300	3.04874800
H	0.27078700	-5.27702900	3.59760000

C	0.98945400	-4.19397700	1.88882400
H	-0.01044300	-3.94484200	1.55020400
C	1.82088200	-3.75804300	-1.40614800
C	1.22803700	-3.18572900	-2.54126500
H	0.81756900	-2.18260400	-2.47605100
C	1.15735900	-3.89004400	-3.73896000
H	0.69220200	-3.43165100	-4.60504600
C	1.67903000	-5.18194700	-3.82573400
H	1.62448100	-5.73206000	-4.75868400
C	2.26545700	-5.75929200	-2.70383500
H	2.67005900	-6.76413500	-2.75781100
C	2.33351400	-5.05308100	-1.50081500
H	2.78000100	-5.52322800	-0.63296900
C	4.10238200	-1.89222900	-0.73900100
H	4.37282300	-2.86713800	-1.12418100
C	6.43999200	-1.10681400	-1.33597800
H	6.66612100	-0.24606600	-1.97438800
C	6.54116500	-2.32413500	-2.25991100
C	7.00908300	-3.56632900	-1.82230200
H	7.33740100	-3.68644000	-0.79652800
C	7.07403600	-4.65239200	-2.69577100
H	7.44107800	-5.60768100	-2.33659500
C	6.67688400	-4.51192100	-4.02289800
H	6.73097200	-5.35506200	-4.70233600
C	6.21322600	-3.27600800	-4.47231500
H	5.90354000	-3.15313900	-5.50424500

C	6.14743200	-2.19531900	-3.59739300
H	5.78084400	-1.23806800	-3.95502300
C	7.51546700	-1.11798600	-0.24448100
C	7.24720200	-1.48921400	1.07511500
H	6.23543200	-1.74763900	1.36500900
C	8.26397600	-1.51604800	2.03079700
H	8.03338200	-1.80317500	3.05090500
C	9.56597700	-1.16793900	1.68160400
H	10.35488200	-1.18367300	2.42513200
C	9.84536500	-0.79123000	0.36775500
H	10.85462200	-0.51336600	0.08426600
C	8.82882600	-0.76825600	-0.58279000
H	9.05700700	-0.47998600	-1.60440500
C	5.02550200	-0.87472200	-0.79117600
C	4.64348100	0.42592700	-0.32613000
C	5.53159700	1.53449300	-0.31083300
H	6.55651400	1.40320600	-0.63064100
C	5.13390200	2.77162700	0.13161800
H	5.85046400	3.58399600	0.14858100
C	3.80960700	2.99600900	0.58073900
C	2.92743300	1.93785600	0.57203600
H	1.91117600	2.09332400	0.91200300
C	3.30827900	0.63852100	0.14074200
C	3.36150100	4.36851100	1.08057000
H	2.39609000	4.20646200	1.57287000
C	3.09469900	5.39105100	-0.03198000

C	2.64595200	6.67245500	0.32193700
H	2.54101800	6.92895000	1.37093300
C	2.34431200	7.62230300	-0.64834300
H	1.99757100	8.60562500	-0.34952000
C	2.48575200	7.31140800	-2.00157100
H	2.24953000	8.04918200	-2.76003200
C	2.92507000	6.04335100	-2.36628800
H	3.03051200	5.78481200	-3.41409900
C	3.22447800	5.09052800	-1.38972600
H	3.55221500	4.10505300	-1.69585900
C	4.30854800	4.88672000	2.16835800
C	5.39318400	5.72288200	1.88372600
H	5.55192400	6.07092200	0.86933000
C	6.26263600	6.13313100	2.89361900
H	7.09755200	6.78184900	2.65188500
C	6.05816500	5.71994500	4.20852200
H	6.73159000	6.04329000	4.99431200
C	4.97586100	4.89333600	4.50606000
H	4.80142500	4.57120600	5.52685300
C	4.11245100	4.48159400	3.49343700
H	3.27350800	3.83603000	3.73393800

Np# (meso)

Energy: -4004.983128 au

Sum of electronic and thermal Energies: -4003.439890 au

Geometry:

N	1.09216000	-0.65782400	0.95487500
C	5.15604100	-0.57034400	-0.34885900
C	4.52154200	0.64162500	0.07978300
C	6.61785000	-0.55100600	-0.80964800
H	7.14760600	0.12106400	-0.12722700
C	3.68558600	-3.89196800	2.06831200
H	4.33935400	-3.03517300	1.96266400
C	3.06583800	-1.80286100	0.06051700
C	3.90183100	-4.78742800	3.11874100
H	4.72063400	-4.61364700	3.80851300
C	7.45127100	-2.81415500	-1.70392600
H	7.06882500	-2.56151500	-2.68569800
C	2.50855200	-3.78719400	-1.38306800
C	0.72432200	-0.48922500	2.29343000
H	1.44265000	-0.34972400	3.08306000
C	3.15529900	0.60264600	0.50555400
C	6.79683700	0.04021900	-2.21157600
C	7.31734800	-1.90450200	-0.65145700
C	4.42155600	-1.73102900	-0.35606200
H	4.89129400	-2.64642700	-0.69181600
C	2.64152900	-4.08942600	1.16199800
C	2.33397800	-3.14271600	-0.00166600
H	1.27075800	-2.91177800	0.07160000
C	3.39704200	-4.83763600	-1.62623500
H	3.97836900	-5.25282800	-0.81098000
C	2.46410400	4.27635700	1.39220900

H	1.40435100	4.11447200	1.16893900
C	2.45413700	-0.64473000	0.49581900
C	2.87037000	5.52474400	0.59890000
C	2.54127800	4.46022300	2.90909500
C	5.81642900	-0.06414100	-3.20155600
H	4.87454400	-0.55104600	-2.97732200
C	3.67921300	4.13074400	3.65241700
H	4.54147200	3.70010100	3.15683700
C	3.07616200	-5.89501000	3.28146900
H	3.24520800	-6.59024700	4.09625100
C	1.81356200	-5.20835600	1.34032000
H	0.99798000	-5.38364400	0.64595700
C	8.08184300	-4.04331100	-1.50815500
H	8.17469000	-4.73497900	-2.33829400
C	1.43699700	4.99289400	3.58498700
H	0.54186900	5.24878800	3.02689800
C	7.99803400	0.68632600	-2.52861400
H	8.77258100	0.77337400	-1.77254400
C	8.47490100	-3.47754000	0.79765900
H	8.87780000	-3.72453000	1.77385400
C	8.59533500	-4.38045700	-0.25836200
H	9.08966400	-5.33376600	-0.10868100
C	2.02628200	-6.10206900	2.38478400
H	1.37283500	-6.96019000	2.49924500
C	3.59251800	6.58255100	1.15563700
H	3.87800200	6.54896200	2.20003000

C	1.75522000	-3.28202500	-2.45158400
H	1.06280200	-2.46562100	-2.26911200
C	2.61204200	4.87221400	5.69202600
H	2.63915900	5.02780800	6.76474900
C	2.84849900	6.71408000	-1.52293500
H	2.55114700	6.75776500	-2.56492300
C	2.50359100	5.60878000	-0.75163000
H	1.94897400	4.79300000	-1.20478600
C	3.71600500	4.33744400	5.03132000
H	4.60800800	4.07408800	5.58937600
C	3.53473100	-5.36984800	-2.90907500
H	4.22856400	-6.18611700	-3.07885400
C	1.46945200	5.19763100	4.96263900
H	0.60000100	5.60647600	5.46567800
C	7.84198300	-2.25331300	0.59935000
H	7.75347800	-1.55601300	1.42707500
C	8.21611000	1.21286800	-3.79875100
H	9.15250700	1.71251200	-4.02176400
C	0.01340600	-0.82493400	0.12447400
C	3.57406000	7.76309300	-0.95638000
H	3.84420400	8.62620300	-1.55438100
C	3.94401100	7.69196700	0.38323100
H	4.50492000	8.50246900	0.83589400
C	6.03281500	0.45974500	-4.47665900
H	5.25856500	0.36932300	-5.23062100
C	7.23152600	1.10002400	-4.78027300

H	7.39694500	1.51128300	-5.76974600
C	2.78267700	-4.86158500	-3.96527900
H	2.88686100	-5.27878100	-4.96083500
C	1.89013300	-3.81482900	-3.73096000
H	1.29510200	-3.41511900	-4.54516300
N	-1.03619300	-0.75381500	1.00465800
C	-5.11394800	-1.14338400	-0.19970100
C	-4.41635300	-2.18524800	0.37176800
H	-4.91620900	-3.13983700	0.49270100
C	-6.56619900	-1.42650100	-0.61007600
H	-6.82218100	-2.34051000	-0.06336300
C	-3.52323000	4.79252200	1.12802100
H	-4.23768800	3.98185200	1.05479900
C	-3.07168500	0.22916500	0.02321900
C	-3.67232400	5.74506600	2.13869900
H	-4.50447900	5.66476500	2.82968800
C	-8.55126900	0.19514400	-0.92313100
H	-8.58162400	-0.05007800	-1.97769200
C	-2.47654900	4.46889900	-2.30334500
C	-0.62502800	-0.54627000	2.32444500
H	-1.31618800	-0.47582300	3.14655400
C	-3.06567600	-2.09511900	0.78736400
C	-6.73569000	-1.80820900	-2.08410200
C	-7.58579700	-0.39139200	-0.10086500
C	-4.44090200	0.10766000	-0.38477000
C	-2.46258000	4.87094600	0.22248700

C	-2.24901700	3.86400600	-0.91309900
H	-1.18879800	3.59183500	-0.87442200
C	-3.35795500	5.52954500	-2.52983300
H	-3.87955400	5.98297000	-1.69485800
C	-2.37195900	-3.30483700	1.41564900
H	-1.30141000	-3.16038900	1.25570900
C	-2.40980400	-0.89393500	0.60433500
C	-2.72696100	-4.60544900	0.68293400
C	-2.55721300	-3.39456200	2.93335300
C	-5.93590400	-1.30393100	-3.11360000
H	-5.13466700	-0.61160800	-2.89131000
C	-3.68430200	-2.90099900	3.59656800
H	-4.46763400	-2.40609600	3.03509600
C	-2.76039300	6.78886400	2.26291600
H	-2.87678500	7.52790000	3.04781000
C	-1.54737300	5.92419100	0.36205100
H	-0.71579100	6.00585900	-0.33073400
C	-9.47990100	1.09922500	-0.40377200
H	-10.21738700	1.54475900	-1.06255300
C	-1.55698800	-4.01242800	3.69540500
H	-0.66857400	-4.39322500	3.20215100
C	-7.74062100	-2.72620100	-2.41870900
H	-8.36585100	-3.13961100	-1.63323200
C	-8.50979200	0.84098000	1.78122700
H	-8.48720400	1.08428900	2.83797600
C	-9.46376100	1.42697000	0.94861700

H	-10.18584700	2.12804200	1.35179400
C	-1.69157800	6.87352200	1.36916600
H	-0.96996300	7.67841600	1.45588700
C	-3.55756600	-5.58625400	1.23011900
H	-3.96570600	-5.45354700	2.22510500
C	-1.79935300	3.91928800	-3.39910500
H	-1.11333800	3.09272500	-3.24153000
C	-2.81321700	-3.64663400	5.72612700
H	-2.91095900	-3.74099500	6.80183900
C	-2.49265800	-5.97369100	-1.31189900
H	-2.06838100	-6.11837000	-2.29926000
C	-2.19731400	-4.81673900	-0.59731100
H	-1.55187700	-4.06382000	-1.03855700
C	-3.81280800	-3.02685900	4.98115400
H	-4.69562400	-2.63568900	5.47521600
C	-3.56356200	6.02305600	-3.81877900
H	-4.25137500	6.84723100	-3.97369400
C	-1.68113800	-4.13859200	5.07598600
H	-0.89055500	-4.61570600	5.64466600
C	-7.58383500	-0.05729900	1.26008600
H	-6.84249900	-0.50069200	1.91664600
C	-7.95270300	-3.11692500	-3.73788200
H	-8.73548600	-3.83114200	-3.96877700
C	-3.32852500	-6.94431600	-0.75844300
H	-3.55969300	-7.84681900	-1.31312100
C	-3.85863300	-6.74609300	0.51326400

H	-4.50520300	-7.49619100	0.95575900
C	-6.14484300	-1.69373000	-4.43723900
H	-5.50925600	-1.29074000	-5.21808400
C	-7.15430900	-2.59776700	-4.75633900
H	-7.31242500	-2.90212200	-5.78488600
C	-2.88522600	5.46773400	-4.90081100
H	-3.04128600	5.85501800	-5.90144000
C	-1.99850400	4.41269000	-4.68560200
H	-1.46043400	3.97517800	-5.51946900
C	5.17820000	1.89984700	0.08375700
C	4.53995700	3.04862600	0.48728700
C	3.19145600	3.01890400	0.91236600
C	2.52742900	1.81064400	0.90492900
H	6.20072300	1.97039300	-0.26218800
H	5.07050900	3.99321100	0.45569100
H	1.49091000	1.78016600	1.21620800
C	-2.40119900	1.46606700	-0.16813100
C	-3.01903800	2.55607100	-0.73833300
C	-4.36715400	2.42500300	-1.15184500
C	-5.05075400	1.24761900	-0.97555800
H	-1.36467600	1.54013300	0.13365700
H	-4.86635900	3.26240400	-1.62520600
H	-6.07945900	1.19022500	-1.29907500

IPr*

Energy: -2773.221419 au

Sum of electronic and thermal Energies: -2772.118909 au

Geometry:

C	-0.05225700	0.00617700	-0.45082500
N	0.31571500	0.98851500	0.43507100
C	0.20418000	0.58414900	1.76645600
H	0.45333600	1.22511700	2.59407100
C	-0.23706200	-0.69274300	1.73708300
H	-0.43163300	-1.39016500	2.53341700
N	-0.39768600	-1.02205400	0.38733500
C	0.82999100	2.26707300	0.01094000
C	-0.05681900	3.25330600	-0.45816700
C	0.47579200	4.46512500	-0.89892800
H	-0.19867200	5.22336000	-1.28012800
C	1.84750000	4.72241400	-0.88157500
C	2.69757500	3.72950200	-0.39994700
H	3.76782500	3.90385600	-0.39741100
C	2.21547900	2.49897700	0.05473500
C	-1.56960800	3.02078500	-0.45399100
H	-1.71778400	1.95356000	-0.63171300
C	-2.21091500	3.32478800	0.90370800
C	-1.65425800	4.20990900	1.83092100
H	-0.71309300	4.69944600	1.61039700
C	-2.29224600	4.46882200	3.04584700
H	-1.84181900	5.15914000	3.75101100
C	-3.49731400	3.84411300	3.35420400
H	-3.99134100	4.04242000	4.29887200

C	-4.06204700	2.95734200	2.43659200
H	-4.99920900	2.46179800	2.66628600
C	-3.42440200	2.70311700	1.22600400
H	-3.87423300	2.01770000	0.51532700
C	-2.26761200	3.73168300	-1.61764300
C	-2.18875200	3.16170000	-2.89478800
H	-1.65735600	2.22441400	-3.02430000
C	-2.78248600	3.78000100	-3.99125600
H	-2.71295000	3.32022700	-4.97118200
C	-3.47035400	4.98328700	-3.83058900
H	-3.93545500	5.46538300	-4.68334600
C	-3.56149700	5.55508400	-2.56433000
H	-4.10063300	6.48607600	-2.42570500
C	-2.96683100	4.93189100	-1.46581900
H	-3.05766000	5.38109700	-0.48363100
C	2.39440600	6.02748000	-1.40824700
H	1.73326800	6.86356800	-1.16719300
H	2.49351200	5.99520900	-2.49865600
H	3.38229600	6.24252000	-0.99521800
C	3.18578300	1.44525700	0.59507300
H	2.69785700	0.47654900	0.46774300
C	4.47259500	1.37121300	-0.23726700
C	4.41321100	0.79282500	-1.51246500
H	3.46690000	0.41083800	-1.88202800
C	5.54761500	0.70648200	-2.31384700
H	5.47872300	0.24853700	-3.29439300

C	6.76861600	1.20246600	-1.85486700
H	7.65432700	1.13536300	-2.47673000
C	6.84021000	1.77787700	-0.58944300
H	7.78474000	2.16200900	-0.21951000
C	5.70143200	1.85807800	0.21479900
H	5.77710200	2.29492400	1.20346900
C	3.45158700	1.58401300	2.09860200
C	3.39519700	2.80648100	2.77400100
H	3.12711700	3.70793300	2.23641500
C	3.67047600	2.88264200	4.14088600
H	3.61946600	3.84187000	4.64467900
C	4.00393400	1.73571800	4.85599300
H	4.21410700	1.79423400	5.91812700
C	4.05862100	0.50900100	4.19354300
H	4.30934500	-0.39419200	4.73904100
C	3.78477400	0.43681500	2.83081400
H	3.82466200	-0.52422200	2.32899600
C	-0.81937700	-2.32210600	-0.06959300
C	0.13906800	-3.21472100	-0.58801700
C	-0.28761600	-4.47342700	-1.01263300
H	0.44122500	-5.15465700	-1.43758500
C	-1.61910600	-4.87564700	-0.91845300
C	-2.54310100	-3.96637900	-0.40930100
H	-3.58445100	-4.25861300	-0.34878900
C	-2.17664300	-2.68478100	0.00855300
C	1.61691500	-2.83828300	-0.71184000

H	1.66728000	-1.75235200	-0.62119200
C	2.13582400	-3.14774000	-2.12182000
C	1.79691700	-2.27256500	-3.16246700
H	1.19592300	-1.39591800	-2.93980700
C	2.21602400	-2.52107700	-4.46675000
H	1.94679000	-1.83047700	-5.25875600
C	2.98074300	-3.65201600	-4.75531700
H	3.30851000	-3.84618100	-5.77058000
C	3.32385000	-4.52672600	-3.72748500
H	3.92261900	-5.40607700	-3.93920400
C	2.90662800	-4.27452900	-2.41961200
H	3.19664800	-4.95456500	-1.62673200
C	2.50584200	-3.39811100	0.40422400
C	3.83046000	-2.94216600	0.49052500
H	4.19678600	-2.22601500	-0.23789100
C	4.68627100	-3.40112900	1.48645700
H	5.70629700	-3.03481000	1.52832200
C	4.23453500	-4.32832800	2.42746400
H	4.89987300	-4.68869200	3.20414000
C	2.92189900	-4.78325600	2.35805800
H	2.55595000	-5.50114600	3.08421600
C	2.06547200	-4.32184400	1.35490700
H	1.04664800	-4.68721400	1.32062700
C	-2.04341700	-6.26020000	-1.34531900
H	-3.09011600	-6.27790600	-1.65696100
H	-1.93486500	-6.97397800	-0.52129300

H	-1.43390300	-6.62435800	-2.17560200
C	-3.26133900	-1.70986300	0.48387300
H	-2.74946800	-0.85213800	0.92555000
C	-4.10237600	-2.32293000	1.60982900
C	-3.71371000	-2.13194300	2.94030300
H	-2.84792500	-1.51587400	3.15855000
C	-4.42716600	-2.70683000	3.99068900
H	-4.10773900	-2.53926400	5.01350900
C	-5.55157400	-3.48618200	3.72763100
H	-6.11051700	-3.93368100	4.54176600
C	-5.95896600	-3.67375700	2.40788800
H	-6.84099700	-4.26656100	2.19106100
C	-5.24430100	-3.09389400	1.36143900
H	-5.59383400	-3.22494600	0.34380300
C	-4.10781800	-1.13834600	-0.66604000
C	-5.23099600	-0.35357500	-0.36440400
H	-5.51826900	-0.20812500	0.67107500
C	-5.99298200	0.23118000	-1.37152200
H	-6.85663000	0.83330400	-1.11092000
C	-5.64722500	0.04572900	-2.70998200
H	-6.23839900	0.50130100	-3.49625900
C	-4.53162200	-0.72396900	-3.02293800
H	-4.24565100	-0.87152900	-4.05866000
C	-3.76777700	-1.30833500	-2.01079400
H	-2.89782700	-1.89393900	-2.27848800

IPr

Energy: -1160.332840 au

Sum of electronic and thermal Energies: -1159.735807 au

Geometry:

C	0.00000000	-0.00000100	0.35069300
N	-1.06505100	-0.00000100	-0.51385900
N	1.06505100	-0.00000100	-0.51385800
C	-0.67657800	-0.00000300	-1.85386300
H	-1.38337900	-0.00000300	-2.66653700
C	0.67658000	-0.00000200	-1.85386200
H	1.38338200	-0.00000200	-2.66653600
C	-2.44274600	0.00000000	-0.08806400
C	-3.09383300	1.23138300	0.10997300
C	-4.43154800	1.20343100	0.51614900
H	-4.95828200	2.13622500	0.68252900
C	-5.09664900	0.00000100	0.71661500
H	-6.13403000	0.00000100	1.03321500
C	-4.43154900	-1.20343000	0.51615000
H	-4.95828300	-2.13622400	0.68253000
C	-3.09383400	-1.23138300	0.10997300
C	-2.38491200	-2.56876700	-0.06946400
H	-1.39300500	-2.36849900	-0.47692600
C	-2.18451300	-3.27009300	1.28749600
H	-1.62172700	-2.63416300	1.97416500
H	-1.63212200	-4.20553600	1.15581500
H	-3.14370000	-3.51126200	1.75564700

C	-3.11394800	-3.48446300	-1.06944000
H	-2.54240100	-4.40425400	-1.22524700
H	-3.23926100	-2.99570300	-2.03942800
H	-4.10626000	-3.77137800	-0.70984000
C	-2.38491000	2.56876600	-0.06946500
H	-1.39300500	2.36849800	-0.47692800
C	-3.11394700	3.48446300	-1.06944000
H	-4.10625800	3.77138000	-0.70983900
H	-3.23926100	2.99570400	-2.03942800
H	-2.54239900	4.40425300	-1.22524800
C	-2.18451000	3.27009200	1.28749500
H	-1.63211800	4.20553400	1.15581400
H	-1.62172300	2.63416100	1.97416400
H	-3.14369600	3.51126200	1.75564700
C	2.44274600	0.00000000	-0.08806100
C	3.09383200	1.23138300	0.10997600
C	4.43154700	1.20343200	0.51615400
H	4.95828000	2.13622600	0.68253300
C	5.09664900	0.00000200	0.71661900
H	6.13402900	0.00000300	1.03322100
C	4.43154900	-1.20342900	0.51615400
H	4.95828300	-2.13622200	0.68253400
C	3.09383400	-1.23138200	0.10997600
C	2.38491300	-2.56876600	-0.06946200
H	1.39300700	-2.36849900	-0.47692500
C	3.11395100	-3.48446200	-1.06943600

H	3.23926500	-2.99570300	-2.03942500
H	2.54240400	-4.40425400	-1.22524400
H	4.10626300	-3.77137700	-0.70983500
C	2.18451300	-3.27009300	1.28749800
H	3.14369900	-3.51126100	1.75565000
H	1.63212200	-4.20553500	1.15581700
H	1.62172500	-2.63416200	1.97416700
C	2.38490900	2.56876600	-0.06946300
H	1.39300400	2.36849700	-0.47692600
C	2.18450800	3.27009300	1.28749700
H	3.14369300	3.51126300	1.75564900
H	1.62172100	2.63416100	1.97416500
H	1.63211500	4.20553400	1.15581500
C	3.11394600	3.48446300	-1.06943800
H	4.10625700	3.77138100	-0.70983600
H	2.54239800	4.40425400	-1.22524600
H	3.23926100	2.99570400	-2.03942600

IMes

Energy: -924.404843 au

Sum of electronic and thermal Energies: -923.986664 au

Geometry:

C	0.00000000	0.00000000	0.27771500
N	0.00000100	1.06472400	-0.58481200
C	0.00000000	0.67628600	-1.92619000
H	0.00000500	1.38319700	-2.73898300

C	0.00000000	2.43849400	-0.15485700
C	1.22346900	3.09011900	0.05062100
C	1.19819400	4.42711400	0.45461900
H	2.14058900	4.93992700	0.62196900
C	0.00000000	5.11441800	0.65640100
C	-1.19819300	4.42712400	0.45458500
H	-2.14058900	4.93994500	0.62190900
C	-1.22346800	3.09012900	0.05058500
C	2.53436800	2.36591300	-0.13007600
H	2.64118600	1.96787700	-1.14317400
H	2.60511200	1.51712100	0.55540300
H	3.37428900	3.03578700	0.06157500
C	0.00000200	6.56908500	1.06149600
H	0.88438600	6.81822800	1.65254000
H	-0.88442100	6.81824800	1.65247400
H	0.00004300	7.22128500	0.18108600
C	-2.53437000	2.36593700	-0.13015100
H	-2.64116500	1.96791300	-1.14325600
H	-2.60514000	1.51713900	0.55531700
H	-3.37428900	3.03581700	0.06148500
N	-0.00000100	-1.06472400	-0.58481200
C	0.00000000	-0.67628600	-1.92619000
H	-0.00000500	-1.38319700	-2.73898300
C	0.00000000	-2.43849400	-0.15485700
C	-1.22346900	-3.09011900	0.05062100
C	-1.19819400	-4.42711400	0.45461900

H	-2.14058900	-4.93992700	0.62196900
C	0.00000000	-5.11441800	0.65640100
C	1.19819300	-4.42712400	0.45458500
H	2.14058900	-4.93994500	0.62190900
C	1.22346800	-3.09012900	0.05058500
C	-2.53436800	-2.36591300	-0.13007600
H	-2.64118600	-1.96787700	-1.14317400
H	-2.60511200	-1.51712100	0.55540300
H	-3.37428900	-3.03578700	0.06157500
C	-0.00000200	-6.56908500	1.06149600
H	-0.88438600	-6.81822800	1.65254000
H	0.88442100	-6.81824800	1.65247400
H	-0.00004300	-7.22128500	0.18108600
C	2.53437000	-2.36593700	-0.13015100
H	2.64116500	-1.96791300	-1.14325600
H	2.60514000	-1.51713900	0.55531700
H	3.37428900	-3.03581700	0.06148500
